# Supplementary material for: Effect of herbal extract granules combined with otilonium bromide on irritable bowel syndrome with diarrhoea: a study protocol for a randomised controlled trial
Source: BMJ Open. 2017 Dec 1;7(11):e018362. doi: 10.1136/bmjopen-2017-018362 (PMC5719308; doi:10.1136/bmjopen-2017-018362)
Supplement: Author's manuscript [file bmjopen-2017-018362.draft_revisions.pdf]

BMJ Open is committed to open peer review. As part of this commitment we make the peer review history of every article we publish publicly available.

When an article is published we post the peer reviewers' comments and the authors' responses online. We also post the versions of the paper that were used during peer review. These are the versions that the peer review comments apply to.

The versions of the paper that follow are the versions that were submitted during the peer review process. They are not the versions of record or the final published versions. They should not be cited or distributed as the published version of this manuscript.

BMJ Open is an open access journal and the full, final, typeset and author-corrected version of record of the manuscript is available on our site with no access controls, subscription charges or pay-per-view fees (http://bmjopen.bmj.com).

If you have any questions on BMJ Open's open peer review process please email editorial.bmjopen@bmj.com

BMJ Open

Effect of herbal extract granules combined with otilonium bromide on irritable bowel syndrome with diarrhea: study protocol for a randomized controlled trial

Journal:	*BMJ Open*
Manuscript ID	bmjopen-2017-018362
Article Type:	Protocol
Date Submitted by the Author:	23-Jun-2017

Complete List of Authors:	Kim, Joong Il; Catholic Kwandong University International Saint Mary's Hospital, Institute for Integrative Medicine;<br>Kim, Pumsoo; Catholic Kwandong University International Saint Mary's Hospital, Institute for Integrative Medicine<br>Lee, Jin-Hyun; Wonkwang University College of Korean Medicine, Department of Korean Rehabilitation Medicine<br>Kim, Yoo-Jin; Catholic Kwandong University International Saint Mary's Hospital, Institute for Integrative Medicine<br>Yang, Na-rae; Catholic Kwandong University International Saint Mary's Hospital, Institute for Integrative Medicine<br>Baeg, Myong Ki; Catholic Kwandong University International Saint Mary's Hospital, Institute for Integrative Medicine<br>Choi, Ja Sung; Catholic Kwandong University International Saint Mary's Hospital, Institute for Integrative Medicine<br>Kim, Hye-Jung; Catholic Kwandong University International Saint Mary's Hospital, Institute for Integrative Medicine<br>Kim, Ja-Young; Catholic Kwandong University International Saint Mary's Hospital, Institute for Integrative Medicine<br>Sunwoo, Yun-Young; Catholic Kwandong University International Saint Mary's Hospital, Institute for Integrative Medicine<br>Lee, Jeong-Han; Wonkwang University College of Korean Medicine, Department of Korean Rehabilitation Medicine<br>Ha, Hyekyung; Korea Institute of Oriental Medicine, K-herb Research Center<br>Park, Tae-Yong; Catholic Kwandong University International Saint Mary's Hospital, Institute for Integrative Medicine

**Primary Subject Heading**:	Complementary medicine
Secondary Subject Heading:	Gastroenterology and hepatology
Keywords:	Samryungbaekchulsan, otilonium bromide, irritable bowel syndrome, randomized controlled study, clinical trial protocol

SCHOLARONE™
Manuscripts

For peer review only

Effect of herbal extract granules combined with otilonium bromide on irritable bowel syndrome with diarrhea: study protocol for a randomized controlled trial

Joong-Il Kim[1*], Pumsoo Kim[1*], Jin-Hyun Lee[2], Yoo-Jin Kim[1], Na-rae Yang[1], Myong Ki Baeg[1], Ja Sung Choi[1], Hye-Jung Kim[1], Ja-Young Kim[1], Yun-Young Sunwoo[1], Jeong-Han Lee[2], Hyekyung Ha[3], Tae-Yong Park[1]

1 Institute for Integrative Medicine, Catholic Kwandong University International St. Mary's Hospital, Incheon, 22711, South Korea

2 Department of Korean Rehabilitation Medicine, Wonkwang University College of Korean Medicine, Iksan, 54383, South Korea

3 K-herb Research Center, Korea Institute of Oriental Medicine, Daejeon, 34054, South Korea

Corresponding author

Tae-Yong Park

Address: Institute for Integrative Medicine, Catholic Kwandong University International, St. Mary's Hospital, Simgokro 100gil 25, Seo-gu, Incheon, 22711, Republic of Korea

E-mail: parktae9822@gmail.com

Telephone: +82-32-290-3319

*** These authors contributed equally to the study**

Funding: This work was supported by a "Construction of Scientific Evidences for Herbal Medicine Formulas (K15250)" grant from the Korea Institute of Oriental Medicine (KIOM) (Telephone: +82-42-868-9513)

Word Count: 4423

For peer review only

Key words: Samryungbaekchulsan, otilonium bromide, irritable bowel syndrome, randomized controlled study, clinical trial protocol

For peer review only

For peer review only - http://bmjopen.bmj.com/site/about/guidelines.xhtml

ABSTRACT

Introduction

Irritable bowel syndrome (IBS) is a collection of symptoms that occur together, generally including pain or discomfort in the abdomen and changes in bowel movement patterns. It is known as a functional gastrointestinal (GI) disorder. Due to the limitations of conventional treatments, alternative IBS treatments are used by many patients worldwide. Samryungbaekchulsan (SRS), an herbal formula, has long been used for alleviating diarrhea-predominant IBS (D-IBS) in traditional Korean medicine. Otilonium bromide (OB) is an antimuscarinic compound used to relieve spasmodic pain in the gut, especially in IBS. Although herbal formulas and western drugs have been administered simultaneously in many diseases including in D-IBS in Korea, only few studies have investigated the effects of combination therapy made up of an herbal formula and a western drug on any diseases including D-IBS.

Methods and Analysis:

This trial is a randomized, double-blinded, placebo-controlled, double-dummy, four-arm, parallel study. After a 2-week preparation period, 80 patients with D-IBS will be randomly assigned to one of the four treatment groups consisting of SRS (water extract granules, 5 g/pack, 3 times a day) with OB (tablet form, 1 capsule 3 times a day) or their placebos, with treatment lasting for 8 weeks. Post-treatment follow-up will be conducted at 4 week after the end of treatment.. The primary outcome is the Subject’s Global Assessment of Relief. The secondary outcomes are the severity of symptoms related to D-IBS using a 10-point scale and the change in symptoms.

Discussion:

For peer review only

For peer review only - http://bmjopen.bmj.com/site/about/guidelines.xhtml

The combination of OB and SRS is expected to improve multiple D-IBS symptoms at the same time—abdominal pain and discomfort (major efficacy of OB), and diarrhea (major efficacy of SRS). This trial is designed to examine the efficacy and safety of SRS and OB combination medication on D-IBS, and to provide clinical evidence for a new therapeutic regimen to treat D-IBS.

Ethics and dissemination:

This trial has full ethical approval of the ethics committee of Kwandong University International St. Mary's Hospital (IS15MISV0033), and Korean Ministry of Food and Drug Safety (30769). The results of the study will be disseminated through a peer-reviewed journal and/or conference presentations.

Trial protocol version: IS15MISV0033 Version 4.0 (July 25, 2016)

Trial registration number: KCT0001621

For peer review only

INTRODUCTION

Irritable bowel syndrome (IBS), a chronic gastro-intestinal (GI) disease and functional bowel disorder, is characterized by abnormal bowel habits and abdominal pain without structural, morphological, and histological abnormalities. It affects between 3–20% of the American population [1, 2]. Based on their predominant bowel habits, patients with IBS can be divided into diarrhea-predominant IBS (D-IBS), constipation-predominant IBS, or mixed IBS [3]. Recently, smooth-muscle relaxants, bulking agents, and anti-diarrheal agents have been commonly used in the treatment of IBS [4]. However, these methods are often not effective, and many IBS patients also use alternative treatments such as herbal medicine, acupuncture, psychological treatment, and lifestyle correction [2, 5, 6].

Samryungbaekchulsan (SRS; Shenlingbaizhu-san in Traditional Chinese Medicine; Jinryobyakujutsu-san in Kampo Medicine), which was introduced in the famous classical compendium of herbal formulas "*Formulary of Peaceful Benevolent Dispensary*", consists of 10 herbs (Atractylodes rhizoma Alba, Poria sclerotium, Dioscoreae rhizoma, Glycyrrhizae radix et rhizoma, Coicis semen, Nelumbinis semen, Platycodonis radix, Dolichoris Semen, Amomi fructus, Ginseng radix). In traditional Korean and Chinese medicine, this herbal formula has been long used to treat GI disease, and various studies have been published on the treatment of IBS with SRS [7-10]. SRS has been shown to have anti-acetylcholine and anti-barium chloride effects, and to suppress gastric secretion and small intestine motility [11]. However, there have been no clinical trials studying the efficacy of SRS on IBS treatment when co-administered with a conventional drug.

Otilonium Bromide (OB) is widely used for the prevention and reduction of symptoms in patients with IBS [12]. OB has been shown to inhibit L- and T-type calcium channels, muscarinic receptors, and tachykininergic responses in human cultured smooth muscle cell and rat colon strips [10]. According to a recent randomized controlled trial (RCT), OB (40

For peer review only - http://bmjopen.bmj.com/site/about/guidelines.xhtml

and 80 mg) is more effective in treating IBS than placebo, not only in the reduction of bloating and abdominal pain, but also in protection from relapse due to its long-lasting effect. [13].

Recently, several studies have been conducted to investigate the therapeutic effect of the co-administration of herbal formulas and conventional drugs on IBS [14, 15]. However, although both SRS and OB have been widely used in the treatment of IBS, there have been no studies on the efficacy and safety of their combined treatment for IBS. Therefore, there is a need to evaluate the efficacy and safety of SRS, OB, and co-administration of both drugs.

In this trial, we will evaluate the safety and efficacy of SRS, OB, and combination therapy on D-IBS, by identifying Subject's Global Assessment of Relief and the severity of symptoms related to D-IBS. The safety of SRS and OB will also be investigated via laboratory tests and the subject's responses.

METHODS AND ANALYSIS

Objectives

The purpose of this study is to identify efficacy and safety of the co-administration of SRS and OB to patients with D-IBS.

Necessity of the trial

Recently, co-administration of herbal medicines and western drugs has been utilized in various medical therapies. This is true particularly in Korea, where very high percentage of patients have used such combined administrations (76%) [16]. However, there are only a few studies on the interaction between western and herbal medicines. Despite the need for combined treatment in patients with various diseases, there are no standard clinical guidelines or scientific evidence on co-administration due to a lack of research and data.

For peer review only - http://bmjopen.bmj.com/site/about/guidelines.xhtml

For peer review only

D-IBS is a disease that is difficult to cure and where many patients required treatment with both western and herbal drugs in order to show an improvement in their symptoms. Therefore, it is very important to proceed with the clinical study on the efficacy and safety of the combined administration of SRS and OB in D-IBS patients.

Basis of drug selection

According to guidelines for IBS treatment, the OB recommendation level is high (i.e., the level of evidence is moderate, and many experts recommend using it), and it is the most reasonable treatment option [17]. SRS is the herbal formula medicine used the most often by clinical Korean and Chinese medical doctors for abdominal pain, abdominal discomfort, and chronic diarrhea, which are typical symptoms of D-IBS [7-10]. Based on these studies and clinical applications, OB and SRS were selected for this trial.

Hypothesis

We hypothesize the following: (1) that co-administration of SRS and OB will have a more positive effect on D-IBS symptoms than either SR or OB alone; (2) that after 8 weeks of taking SRS, OB, or co-administration of both drugs, the Subject's Global Assessment of Relief and severity of D-IBS symptoms will be improved more after treatment with placebo; (3) that co-administration of SRS and OB will be safe for patients with D-IBS.

Design

This study is a placebo-controlled, double-blind trial. Eighty patients will be enrolled and randomly assigned to four separate arms of the trial. The trial will be conducted at Catholic Kwandong University International St. Mary's Hospital, Incheon, South Korea.

The study subjects will be required to participate in a 2-week preparation period (weeks -2

For peer review only - http://bmjopen.bmj.com/site/about/guidelines.xhtml

to 0) and an 8-week drug administration period (weeks 0 to 8). Visiting of the patients will occur four times, on weeks 0, 4, 8, and 12. The primary outcome measurement will be investigated at weeks 2, 4, 6, 8, and 12, and the secondary outcome measurement will be investigated at weeks 0, 2, 4, 6, 8, and 12. Participants will be assessed for efficacy and safety of the treatment through visits (0, 4, 8, 12 weeks) and telephone questionnaires (2, 6 weeks). In addition, lab tests will be conducted at 0, 4, and 8 weeks. According to the intervention protocols, CRC (Clinical Research Coordinator) will encourage patients and participants to complete follow-up in every visits.

During the period of drug administration, SRS or its placebo will be given as 1 pack of water extract granules, 3 times a day, 30 min before each meal. OB or its placebo will be given as 1 tablet, 3 times a day, 30 min before each meal.

All participants will be divided into 4 groups: (1) the SRS and OB group; (2) SRS and placebo-OB group; (3) placebo-SRS and OB group; (4) placebo-SRS and placebo-OB group. The entire flow chart of the study is shown in Figure 1.

The entire protocol of this trial has been approved by the institutional review board (IRB) and ethics committee of Kwandong University International St. Mary's Hospital. The IRB permission number is IS15MISV0033, and CRIS (Clinical Research Information Service, www.cris.nih.go.kr) protocol identification number is KCT0001621. In addition, this study received the first approval given by the Korean Ministry of Food and Drug Safety (KMFDS) for a study of co-medication of a conventional drug and a Korean traditional herbal medicine (approval number: 30769).

After participants will be provided with sufficient information about the study, only those who agree to the study protocol and sign the consent form, will be enrolled. The study will be implemented according to the Good Clinical Practice and the revised version of the Declaration of Helsinki.

For peer review only

Sample size calculation

This trial is a pilot study of a new therapeutic regimen of SRS and OB combined treatment. In a former similar study using co-administration of western and herbal medicine (placebo-controlled, double-blind, and randomly 4-arm), each arm consisted of at least 15 patients and a total sample size of more than 60 participants [14]. In other similar pilot studies, clinical trials have been conducted on a total of 60–64 participants, consisting of 12–30 patients per group [18, 19]. Based on these previous studies, minimal clinical significance of the trial can be obtained by 16 participants who complete the trial. Since drop-out occurrence is expected to be 20%, each trial group will be made up of at least 20 participants. Therefore, the total sample size must be more than 80 participants.

Inclusion criteria

Patients included in the study should match the following criteria: (1) 18–75 years old; (2) suffer from the Rome III criteria of IBS (recurrent abdominal pain or discomfort that began at least 6 months before the clinical trial and rates more than 3 on a 10 point scale, and at least 3 days/month in last 3 months associated with two or more of the following: improved with defecation, onset associated with a change in frequency of stools, and onset associated with a change in form of stool) [1, 20]; (3) in accordance with the Rome III criteria of IBS, D-IBS type defined as loose / mushy or watery stools—Bristol Stool Form (BSF) Scale 6-7 $\geq$ 2 times / week [20, 21]; (4) urine pregnancy test is negative (childbearing age of women, within 7 days before clinical trial) and will use contraception during the administration period; (5) patients who can read the symptom questionnaire and understand its meaning; (6) patients who agree with the clinical plan and voluntarily sign the IRB-approved documents.

For peer review only - http://bmjopen.bmj.com/site/about/guidelines.xhtml

Exclusion criteria

Patients who meet the following conditions will be excluded from the study: (1) chronic liver disease (cirrhosis, chronic hepatitis - B and C); (2) chronic renal failure or renal impairment (serum creatinine ≥ 2 times the upper limit of the normal); (3) liver dysfunction (AST / ALT ≥ 3 times the upper limit of the normal); (4) diabetes (HbA1c > 8%, or not controlled by diet or medication), hypertension (≥ 160/100 mmHg), thyroid dysfunction (exceptions in the following cases: if the disease is controlled during drug administration with a stable dose for 12 weeks before the screening, and the drug dose remains constant during the trial period), clinically significant hematologic, cardiac, pulmonary, neurological disorders or other severe systemic disorders; (5) abnormal findings on colonoscopy or colonography within the last 5 years; (6) history of surgery that affects gastrointestinal motility (i.e., gastrectomy, colonic resection, hysterectomy, except for appendectomy); (7) GI disease as follows, within 6 months before the clinical trial: inflammation or ulcer of esophagus, stomach, or duodenum; gastroesophageal reflux disease (at the discretion of the examiner, subjects may enroll if the disease is chronic and not acute); gastrointestinal bleeding; gastrointestinal stenosis or closure; infectious diarrhea; inflammatory bowel disease (i.e., Crohn's disease, diverticulitis, ulcerative colitis, infectious enteritis, ischemic colitis); pancreatic insufficiency; biliary abscess; (8) mental illness or an addiction to drugs or alcohol; (9) severe systemic organ diseases such as cancer, autoimmune disease, stroke (although patients in recurrence for more than 5 years from cancer unrelated to the GI tract can participate in trial); (10) pregnant and lactating women, and women who are not willing to use contraception during the trial; (11) a history of taking the following drugs within 2 weeks before the trial: antibiotics, anti-inflammatory drugs, analgesics, antidepressants, anti-anxiety agents, anti-inflammatory agents, anti-ulcer agents, anti-gastric secretion inhibitors, laxatives,

For peer review only

antispasmodics, antacid agents, gastrointestinal stimulants, prostaglandin agents, and corticosteroids; (12) use of medications that do not match the intent of this trial, or have a clinical interaction with SRS or OB; (13) lactose intolerance (not controlled by food); (14) glaucoma; (15) patients who have participated in other clinical studies within 30 days prior to clinical screening, and have received other clinical trial medications (including placebo); (16) hypersensitivity to clinical trial medicines; (17) other reasons considered inappropriate for participation in clinical trials.

Recruitment, Randomization, Blinding and Unblinding

Advertisements of the study will be announced in the Catholic Kwandong University International St. Mary's Hospital using banner ads and posters. We also plan to post the advertisements on the homepage of the hospital, in the monthly hospital magazine, at the internet café, and on the subway at least five times. All these advertisements will be conducted with the approval of the IRB.

After recruitment, patients will randomized to one of the four trial arms. Randomization will be performed using a list of block randomizations made by statisticians, independent of this clinical trial. A researcher who is not involved in process of drug prescription and evaluation will execute the randomization process.

During the treatment period, the subjects and all researchers (investigators, clinical research coordinators, management pharmacists) will be blinded, except the researchers who conducted the randomization procedure. Allocation concealment will be performed during the process of medication administration. A management pharmacist who is not affect the results of the trial and analysis, will refer to the randomization list and administer the medication. The patients will take the drugs contained in the opaque envelope, which is labeled with the assignment number.

For peer review only - http://bmjopen.bmj.com/site/about/guidelines.xhtml

Unblinding will be performed only in the following situations: (1) If the trial is terminated and there is a need for statistical analysis; (2) If a serious medical emergency occurs and information about the medication is needed; (3) If the chief investigator determines that unblinding is necessary.

The entire process of the study will be directed by the authorized clinical research organization (CRO), Dream CIS Corporation, Seoul, Korea.

Intervention

The herbal formula SRS is commonly used by Korean traditional medical doctors for treatment of diarrhea and related diseases [7-10]. SRS used in this study (Samryungbaekchulsan granule®, Hankook shin yak, Co., Ltd., Nonsan, Korea) is extracted with water and mixed with starch and lactose in accordance with the Korean Good Manufacturing Practice (K-GMP). Production of SRS is regulated and allowed by the KMFDS. SRS contains 10 herbs, listed in Table 1. Placebo-SRS is made from cornstarch and has the same taste, shape, and color as SRS. SRS and placebo-SRS will be sealed in identical aluminum bags with the same labelling. These herbal drug packages will be administrated by an independent pharmacist in an isolated room. After dissolving the granules in boiled water, the subjects will take them 30 min before each meal.

OB (Menoctyl Tab®, Dong Hwa Pharm Co., LTD., Seoul, Korea) is a tablet containing otilonium bromide 40 mg. Placebo-OB is a tablet made of Ludipress with same taste, shape, and color as OB. Patients will be instructed to swallow one tablet of OB or placebo-OB, 30 min before each meal for the administration period. All of the drugs used in the trial (SRS, OB, placebo-SRS, placebo-OB) have been approved by the KMFDS. Patients will be asked at the end of the study whether the drugs that they have been taking are either real or placebo, to

For peer review only - http://bmjopen.bmj.com/site/about/guidelines.xhtml

measure the success of blinding.Compliance will be confirmed by counting returned SRS packets or OB tablets. Every patient will be instructed to write down any adverse events during the administration period, and these records will be checked at the follow-up. At the time of patients visit (0, 4, 8 week), we will check vital signs, physical examination, electrocardiogram, and perform laboratory tests to evaluate the safety of the patients. All side effects will be reported to the investigator.

Table. 1 Ingredients of the herbal formula Samryungbaekchulsan (SRS)

Scientific name	Part of use	Gram/day
Atractylodis Rhizoma Alba	Root	3.0
Poria Sclerotium	Dried core	4.0
Rhizoma Dioscoreae	Root	3.0
Glycyrrhizae Radix et Rhizoma	Root	1.5
Semen Coicis	Seed	8.0
Semen Nelumbinis	Fruit	4.0
Platycodonis Radix	Root	2.5
Dolichoris Semen	Seed	4.0
Amomi Fructus	Fruit	2.0
Ginseng Radix	Root	3.0

Rescue therapy and concomitant medications

Participants are prohibited from taking any medication that could affect D-IBS during the entire course of the trial. However, if serious side effects or worsening of the symptoms occur, investigators will provide quick and appropriate treatment (including drug administration), based on their medical judgement. In this case, the direct investigators will record the

For peer review only - http://bmjopen.bmj.com/site/about/guidelines.xhtml
For peer review only

treatment or drug administration, and report to the IRB within 24 hours to determine whether the trial should be continued or discontinued.

Outcome Measurements

Primary outcome

The primary outcome will be measured via a questionnaire that confirms the subjective symptom improvement. Participants will respond a total of five times during and after the administration period (2, 6 weeks: through telephone, and 4, 8, 12 weeks: through direct visits) to the following question: Subject's Global Assessment of Relief; "How much do you think the symptoms of D-IBS have improved compared to before the clinical trial?" According to the answers of patients to this survey, the improvement is given a score of 0, 1, 2, 3, or 4.

Secondary outcomes

At 2, 4, 6, 8, and 12 weeks from the start of the treatment period, patients will be assessed for severity of D-IBS symptoms (abdominal pain, abdominal discomfort, satisfaction of defecation, frequency of abdominal pain, quality of life) by using a Likert scale ranging from 0–10, [10, 14, 22]. Patients will also be instructed to record the number of defecations per day, BSF, and the degree of force used in bowel movements at 0, 2, 4, 6, 8, and 12 weeks [14, 19, 21].

Safety assessments

The investigator will record all adverse events that occur during the trial along with any concomitant medications in the case reports. When an adverse event occurs, the investigator will record the symptoms and signs of the adverse reaction, the duration (start and end date),

For peer review only - http://bmjopen.bmj.com/site/about/guidelines.xhtml

For peer review only

severity, course, outcome, significance, causality with the trial drug, and any action taken in relation to the adverse event. In the case of concomitant medicines, composition, dosage, duration of administration, and reason for medication will be recorded in detail. Symptoms that existed before the start of the clinical trial will not be recorded as adverse events.

Laboratory tests

The purpose of laboratory tests in this study is to evaluate the safety and pharmacokinetic profiles of SRS and OB. Because of the small number of patients assigned to this study and the different baseline values for each individual patient, we will not perform a detailed statistical analysis. However, if significant abnormal results are founded in the laboratory tests, the clinical laboratory results will be described and the connection to the drug will be considered.

Blood tests will be conducted at 0, 2, 4, and 8 weeks, and urine tests will be performed at 0 and 4 weeks of the clinical trial. In the case of a participant who drops out or the early termination of the clinical trial, blood and urine tests will be performed on the last day of the trial.

Using the test results (comprehensive verifications of vital signs, laboratory tests, electrocardiograms, and physical examinations) for each individual patient, the investigator will determine adverse reaction of the drugs, and statistical tests will be conducted as needed on clinically significant parameters.

Test of human derivatives

IBS is known to be closely related to stress [6]. Therefore, we plan to quantify factors associated with stress or that have been reported to worsen IBS symptoms as follows: 1) cortisol; 2) corticotropin-releasing hormone (CRH); 3) serotonin; 4) cytokines group I (10

For peer review only - http://bmjopen.bmj.com/site/about/guidelines.xhtml

species); 5) cytokines group II (14 species); 6) growth factors (3 species) [23, 24]. Serum will be separated from blood specimens collected at 0, 4, 8 weeks, stored frozen, and transferred to the Korea Institute of Oriental Medicine (KIOM) for examination.

Early termination or dropout

The criteria for early termination or dropout are as follows: 1) administration of other drugs that are expected to affect the safety and efficacy of the clinical trial drugs; 2) request of participants to discontinue the clinical trial, or withdrawal of the trial agreement; 3) occurrence of significant adverse drug reaction or event that precludes continuing in the trial, according to the judgment of the clinical investigator; 4) new discovery of violations of significant clinical trial protocol during clinical trial; 5) Less than 70% trial drug compliance; (6) any other reason that the clinical trial should be discontinued at the discretion of the clinical trial manager or investigator.

Data collection, access and management

The data of this trial will be managed according to the Standard work instructions of Catholic Kwandong University International, St. Mary's Hospital. Other contents not specified in the trial protocol shall follow the standards of The International Conference on Harmonization of Technical Requirements for Registration of Pharmaceuticals for Human Use (ICH)-Guideline for Good Clinical Practice (GCP) and guide line Korean Good Clinical Practice (KGCP).

The source document is recorded immediately when the data of case collected. After the source document input is completed, it will be recorded in the case record form (CRF). All the documents will be kept safe, so that it can be verified by the relevant government agencies and IRB. Only research colleagues and others who have delegated the approval of

For peer review only - http://bmjopen.bmj.com/site/about/guidelines.xhtml

the principle investigator have access to all data obtained from this trial.

Statistical analysis

The statistical analysis will be conducted by blinded professional statisticians. To evaluate the efficacy of the study, both the *intent-to-treat* (ITT) analysis (main analytical method) and the *per-protocol* (PP) analysis (secondary analytical method) will be performed simultaneously. The ITT analysis will be conducted on all patients who were followed up at least once after the randomization and will be able to evaluated for effectiveness, PP analysis will be performed on patients who have completed for 8 weeks treatment course meeting all of the inclusion and exclusion criteria.

To analyze for continuous variables of demographic information and pre-treatment characteristics between groups, a one-way analysis of variance (ANOVA) and Kruskal-Wallis test will be conducted. An ANOVA and Kruskal-Wallis test will be also used to test for symptom improvement compared to baseline (0 week), and the variables for analysis that include the following: (1) subjective symptom improvement (primary outcomes); (2) severity of symptoms related to D-IBS (abdominal pain, abdominal discomfort, satisfaction of defecation, frequency of abdominal pain, quality of life). All values will be shown as mean ± standard error of the mean (SEM). Comparisons of outcome assessments between the four groups will be performed using the paired t-test or Wilcoxon signed-rank test.

If the Subject's Global Assessment of Relief on the Likert scale is "0" or "1" for more than 2 weeks between weeks 8 and 12, then the symptoms will be considered to have improved sufficiently. Based on this, we will calculate the symptom relief rate by dividing the number of participants who exhibited sufficient symptom relief by the total number of participants in the trial, and present descriptive statistics. The CATMOD procedure will be used to evaluate the relief rate improvement of symptom, compared with baseline. Comparisons between the

For peer review only - http://bmjopen.bmj.com/site/about/guidelines.xhtml

four groups will be conducted using the chi-square test or Fisher's extract test. These statistical analyses will also be performed at 2, 4, 6, 8, and 10 weeks from the baseline. The Cochran-Mantel-Haenszel (CMH) test will be used to confirm the difference between the intervention groups, at each time-point.

A statistical analysis will also be conducted to evaluate safety. The incidence of adverse events and abnormality of experimental results will be analyzed statistically according to the groups, and non-parametric methods will be applied as necessary using a paired t-test for continuous data, McNemar's test for categorical data, Fisher's exact test for adverse events, and a generalized estimating equation for clinically significant changes between groups.

All statistical analysis will be conducted using SAS software, version 9.1.3 (SAS institute, Cary, NC). A P-value <0.05 will be considered statistically significant.

Quality control and Data monitoring

To ensure the results and quality of clinical trials, assessments and monitoring will be performed by the CRO (DreamCIS Corporation, Seoul, Korea), which is independent from the sponsor and competing interests. During the trial, the CRO will monitor regularly whether the study is proceeding in accordance with the protocol through the use of all related documentation, including trial master files, case report forms, informed consent form, and adverse event reports.

For peer review only - http://bmjopen.bmj.com/site/about/guidelines.xhtml

DISCUSSION

Herbal medicine has been recognized as an effective treatment method based on accumulated clinical experience for thousands of years, and has more recently gained attention as an alternative therapy for treating various diseases which are difficult to treat or have unknown causes such as IBS [15, 25]. SRS has frequently been used for abdominal pain and diarrhea in Korean and Chinese traditional medicine. According to experimental research, SRS has an anti-cholinergic effect, increasing the recovery of intestinal digestion and absorption, and inhibiting intestinal movement, associated with the prevention of diarrhea and abdominal pain [11, 26]. In addition, clinical studies have shown that the use of SRS in D-IBS patients is effective in improving diarrhea [27, 28]. However, although there is a growing interest in the use of herbal medicine such as SRS in IBS, and related studies are continuously being published, there are only few well-designed RCTs. To overcome the limitations of these previous studies, this trial was designed as a double-blind, double-dummy, four-arm, parallel RCT. The efficacy of SRS will be evaluated via the Subject's Global Assessment of Relief and the improvement of symptoms associated with bowel movements and abdominal pain. The results of these assessments are expected to provide distinct clinical evidence for SRS in D-IBS treatment.

OB is one of the representative medicines for D-IBS treatment [12]. It is a spasmolytic agent, which exerts its activity primarily on the distal GI tract through the inhibition of Ca^{++} flux and direct activation of contractile proteins in the smooth muscle [29]. OB has also been shown to reduce hyper-motility and visceral sensation modulation, which are thought to help improve D-IBS symptoms [30]. RCTs using OB as an intervention for the treatment of D-IBS have been conducted. In these studies, OB showed a stronger improvement of symptoms related to D-IBS—defecation frequency, regular intestinal habits, reductions in frequency of diarrhea, abdominal pain and discomfort, severity of abdominal bloating—and protection

For peer review only - http://bmjopen.bmj.com/site/about/guidelines.xhtml

from symptom relapse, than placebo [13, 31, 32].

IBS is a disease with a high rate of disease progression and frequent recurrence. For this reason, IBS patients often require various treatment methods [2]. Among these methods, the combination of herbal and conventional drugs is widely used, and are more effective in improving IBS symptoms than conventional drug alone [31]. However, there are several limitations to the co-administration of herbal and conventional drugs in IBS. Specifically, there is a high risk of bias in related studies, and there are insufficient clinical guidelines on the interaction of herbal and conventional drugs in D-IBS, including SRS and OB. Therefore, it is necessary to perform well-designed RCTs studying the co-administration of herbal and conventional drugs.

This RCT has several characteristic features. First, this trial will be the first study to investigate the efficacy of SRS and OB combination therapy for D-IBS. The results of previous studies suggest that the combination of OB and SRS will improve both major D-IBS symptoms at the same time: abdominal pain and discomfort (major efficacy of OB), and diarrhea (main efficacy of SRS). This study will assess this hypothesis, and provide a basis for SRS and OB combination therapy in D-IBS. Second, the participants in this trial will be grouped into four arms. Through this sorting, it will be possible to compare the efficacy of combined treatment with the efficacy of each individual treatment effect. Third, this trial will evaluate the safety of the subjects using several assessments: a periodic assessment of the subjective symptom, physical measurements, and laboratory testing. The results of the safety analysis may provide evidence for the combination treatment of herbal and conventional drug on D-IBS. Fourth, this trial was the first clinical study related to the combination medication of western and traditional herbal medicine in Korea to receive approval of the KMFDS. Through the systematic management of the trial under the auspices of the KMFDS, a higher quality of trial will be secured, and the results of this study will be more helpful in providing

For peer review only - http://bmjopen.bmj.com/site/about/guidelines.xhtml

information for use in clinical applications later.

Although our study is a pilot study with a small sample size, it has a creative and systematic research design, and will be able to be used as basic clinical evidence in the field of integrated herbal and conventional drugs for the management of D-IBS.

Trial status

The research plan and design began in April, 2015.

The study received the IRB's final approval after two deliberations. The first deliberation submission date was July 01, 2015; the result of first was a request for revision and supplementation, which was received on July 13, 2015. We filed an application for re-review on August 06, 2015, supplemented with the relevant information, and received IRB's final approval on August 10, 2015. We also requested that the KMFDS review the trial plan on September 14, 2015 and received approval for the clinical research on December 21, 2015.

The first participant was randomized on 29 March, 2016. Recruitment of the study is ongoing.

Ethics

Research ethics approval

This trial has been approved by IRB and ethics committee of Kwandong University International St. Mary's Hospital (approval number IS15MISV0033), and KMFD (approval number 30769).

Protocol amendments

The investigator who wishes to change the protocol of a clinical trial plan should first discuss it with the trial director. Thereafter, the examiner should obtain prior approval from

For peer review only - http://bmjopen.bmj.com/site/about/guidelines.xhtml

the IRB for changes to the protocol in advance (except to prevent immediate injury to the patient, in which case it will be reported to the IRB later). However, when a dangerous situation occurs to the patient and immediate treatment is needed, the investigator may report the protocol change to the IRB at a later time.

Consent

The patients who are recruited through the announcement will be explained the details of the trial in an easy-to-understand manner. The patients and investigators should have a mutual enough question and answer time, before signature to the clinical trial consent. Through these processes, if the patients agree to participate in the trial, the consent form will be written.

Confidentiality

All the information of patients will be anonymized with initials or symbols, and all related investigators should keep confidentiality of the trial results. The trial director should keep the signed consent form, and prepare the list, when to confirm the patient's identity.

post-trial care

If the patients receive unexpected accidents or injuries, appropriate compensation will be performed by Federal Insurance Company Korea (12th Floor Ferrum Tower 66 Suha-Dong Jung-gu Seoul, 100210, South Korea), according to the patient's compensation rules of the trial. Also in the case of an emergency, the patients will receive an appropriate medical care with the Catholic Kwandong University International, St. Mary's Hospital

Dissemination

For peer review only - http://bmjopen.bmj.com/site/about/guidelines.xhtml
For peer review only

The results of the trial will be disseminated through a peer-reviewed journal and/or conference presentations.

For peer review only

For peer review only - http://bmjopen.bmj.com/site/about/guidelines.xhtml

REFERENCES

1 Drossman DA. Introduction. The Rome Foundation and Rome III. Neurogastroenterol Motil. 2007;19:783-6.

2 Grundmann O, Yoon SL. Irritable bowel syndrome: epidemiology, diagnosis and treatment: an update for health-care practitioners. J Gastroenterol Hepatol. 2010;25:691-9.

3 Longstreth GF, Thompson WG, Chey WD, *et al.* Functional bowel disorders. Gastroenterology. 2006;130:1480-91.

4 Jailwala J, Imperiale TF, Kroenke K. Pharmacologic treatment of the irritable bowel syndrome: a systematic review of randomized, controlled trials. Ann Intern Med. 2000;133:136-47.

5 Liu JP, Yang M, Liu YX, *et al.* Grimsgaard S. Herbal medicines for treatment of irritable bowel syndrome. Cochrane Database Syst. Rev. 2006;1:CD004116.

6 Palsson OS, Drossman DA. Psychiatric and psychological dysfunction in irritable bowel syndrome and the role of psychological treatments. Gastroenterol Clin North Am. 2005;34:281-303.

7 Duan WK. Observation of sixty-five cases on irritable bowel syndrome treated by Trimebutine and Shenlingbaizhu-san granule [Chinese]. Journal of Practical Traditional Chinese Medicine. 2010;26:320.

8 Chen YJ. Clinical observation of irritable bowel syndrome treated by shenlingbaizhu-san granule and trimebutine [Chinese]. Journal of Practical Traditional Chinese Medicine. 2011;27:318-9.

9 Li Q, Jiang JL. Clinical observation of 80 cases on diarrhea - predominant irritable bowel syndrome treated by Shenlingbaizhu-san granule [Chinese]. Guiding Journal of

For peer review only - http://bmjopen.bmj.com/site/about/guidelines.xhtml

Traditional Chinese Medicine and Pharmacy. 2013;19:41-2.

10 Martinez-Cutillas M, Gil V, Gallego D, *et al.* Jimenez M. Mechanisms of action of otilonium bromide (OB) in human cultured smooth muscle cells and rat colonic strips. Neurogastroenterol Motil. 2013;25:e803-12.

11 Song DS, Ryu BH, Park DW, *et al.* Experimental studies on the efficacy of Samryungbaekchulsan. J Korean Med. 1977;14(1):179-95.

12 Boeckxstaens G, Clave P, Corazziari ES, *et al.* Irritable bowel syndrome: focus on otilonium bromide. Expert Rev Gastroenterol Hepatol. 2014;8:131-7.

13 Chmielewska-Wilkoń D, Reggiardo G, Egan CG. Otilonium bromide in irritable bowel syndrome: a dose-ranging randomized double-blind placebo-controlled trial. World J Gastroenterol. 2014;20:12283-91.

14 Li CT, Wu MP, Wang LH, *et al.* Clinical observation of diarrhea - predominant irritable bowel syndrome treated by Shenlingbaizhu-san and Paroxetine [Chinese]. Chinese Journal of Traditional Medical Science and Technology. 2010;17(2):154-5.

15 Leung WK, Wu JC, Liang SM, *et al.* Treatment of diarrhea predominant irritable bowel syndrome with traditional Chinese herbal medicine: a randomized placebo-controlled trial. Am J Gastroenterol. 2006;101:1574-80.

16 Park JK, Choi SY, Koh KW, *et al.* Combined utilization with herbal products and prescribed drugs: a result from health examinee-based national survey. Korean J Health Policy Admin. 2006;16(1):1-16.

17 Kwon JG, Park KS, Park JH, *et al.* The Korean Society of Neurogastroenterology and Motility. Guidelines for the treatment of irritable bowel syndrome. Korean J Gastroenterol. 2011;57(2):82-99.

18 Cha BK, Jung SM, Choi CH, *et al.* The effect of a multispecies probiotic mixture on the symptoms and fecal microbiota in diarrhea-dominant irritable bowel syndrome: a

For peer review only

For peer review only - http://bmjopen.bmj.com/site/about/guidelines.xhtml

randomized, double-blind, placebo-controlled trial. J Clin Gastroenterol. 2012;46(3):220-7.

19 Kim HJ, Vazquez Roque MI, Camilleri M, *et al.* A randomized controlled trial of a probiotic combination VSL #3 and placebo in irritable bowel syndrome with bloating. Neurogastroenterol Motil. 2005 ;17:687-96.

20 Longstreth GF, Thompson WG, Chey WD, *et al.* Functional bowel disorders. Gastroenterology. 2006;130:1480-91.

21 Lewis HJ, Heaton KW. Stool form scale as a useful guide to intestinal transit time. Scand J Gastroenterol. 1997:32:920-4.

22 Chambers CT, Giesbrecht K, Craig KD, *et al.* Comparison of faces scales for the measurement of pediatric pain: children's and parents' ratings. Pain. 1999; 83:25-35.

23 Sugaya N, Izawa S., Saito K, *et al.* Effect of prolonged stress on the adrenal hormones of individuals with irritable bowel syndrome. Biopsychosoc Med. 2015;9:4.

24 Fadgyas-Stanculete M., Buga AM, Popa-Wagner A, *et al.* The relationship between irritable bowel syndrome and psychiatric disorders: from molecular changes to clinical manifestations. J Mol Psychiatry. 2014;2(1):4.

25 Jailwala J, Imperiale TF, Kroenke K. Pharmacologic treatment of the irritable bowel syndrome: a systematic review of randomized, controlled trials. Ann Intern Med. 2000;133:136-47.

26 Li YL. Clinical and experimental study on the treatment of children diarrhea by granule of children-diarrhea fast-stopping. Zhong Xi Yi Jie He Za Zhi. 1991;11:79-82.

27 Zhong YL, Wu YY. Forty cases of diarrhea - predominant irritable bowel syndrome treated by Shenlingbaizhu-san granule and Montmorillonite powder [Chinese]. Jiangxi Journal of Traditional Chinese Medicine. 2013;44:45-6.

For peer review only

For peer review only - http://bmjopen.bmj.com/site/about/guidelines.xhtml

28 Xiong B. Thirty cases of irritable bowel syndrome treated by Shenlingbaizhu-san granule and Probiotics [Chinese]. Jiangxi Journal of Traditional Chinese Medicine. 2011;42(5):22-3.

29 Giachetti A. Pharmacological studies on otilonium bromide. Ital J Gastroenterol. 1991;23:56-9.

30 Evangelista S. Otilonium bromide: a selective spasmolytic for the gastrointestinal tract. J Int Med Res. 1999;27:207-22.

31 Clave P, Acalovschi M, Triantafillidis JK, *et al.* OBIS Study Investigators. Randomised clinical trial: otilonium bromide improves frequency of abdominal pain, severity of distention and time to relapse in patients with irritable bowel syndrome. Aliment Pharmacol Ther. 2011;34:432-42.

32 Battaglia G, Morselli-Labate AM, Camarri E, *et al.* Otilonium bromide in irritable bowel syndrome: a double-blind, placebo-controlled, 15-week study. Aliment Pharmacol Ther. 1998;12:1003-10.

For peer review only - http://bmjopen.bmj.com/site/about/guidelines.xhtml

Authors' Contributions

Joong-Il Kim, Pumsoo Kim, Jin-Hyun Lee, and Jung-Han Lee contributed to making the protocol design and writing the manuscript. Hyekyung Ha is responsible for monitoring the process of the trial. Na-rae Yang, Yoo-Jin Kim, Myong Ki Baeg, Ja Sung Choi, Hye-Jung Kim and Ja-Young Kim provide advice on the process of the trial. Tae-Yong Park is responsible for writing the manuscript and also managing and supervising the clinical research. All authors approved of the final manuscript.

Funding Statement

This work was supported by a "Construction of Scientific Evidences for Herbal Medicine Formulas" grant from the Korea Institute of Oriental Medicine (KIOM) grant number K15250.

Competing interests

The authors have no competing interests to declare.

For peer review only

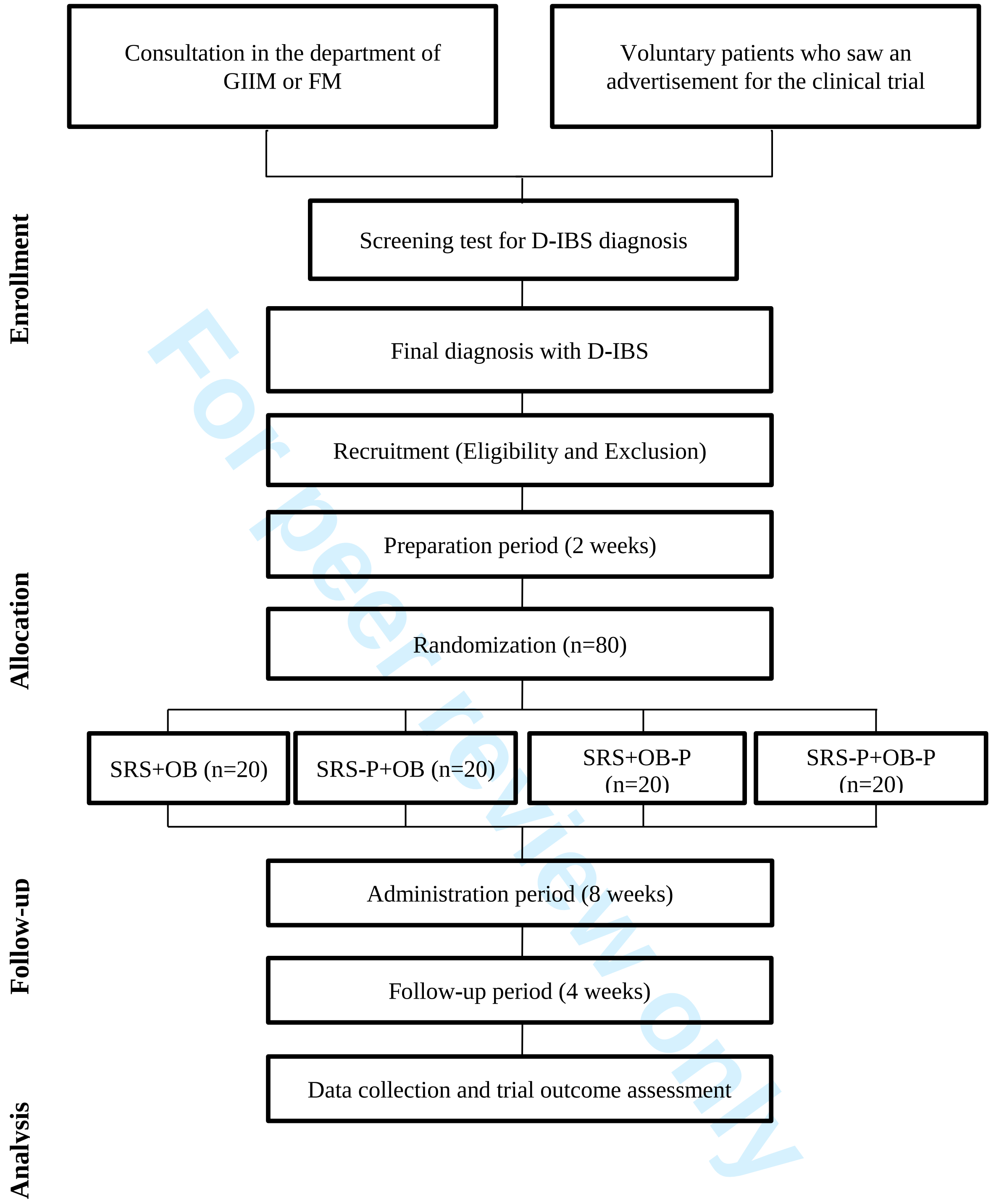

Figure 1. Flow chart of Trial. GIIM: Gastrointestinal internal medicine, FM: Family medicine, D-IBS: diarrhea-irritable bowel syndrome, P: placebo, OB: otilonium bromide, SRS: Samryungbaekchulsan

SPIRIT

STANDARD PROTOCOL ITEMS: RECOMMENDATIONS FOR INTERVENTIONAL TRIALS

SPIRIT 2013 Checklist: Recommended items to address in a clinical trial protocol and related documents*

Section/item	Item No	Description	
**Administrative information**			
Title	1	Descriptive title identifying the study design, population, interventions, and, if applicable, trial acronym	P1
Trial registration	2a	Trial identifier and registry name. If not yet registered, name of intended registry	P4
	2b	All items from the World Health Organization Trial Registration Data Set	Attachment
Protocol version	3	Date and version identifier	P4
Funding	4	Sources and types of financial, material, and other support	P1, P28
Roles and responsibilities	5a	Names, affiliations, and roles of protocol contributors	P1, P28
	5b	Name and contact information for the trial sponsor	P1
	5c	Role of study sponsor and funders, if any, in study design; collection, management, analysis, and interpretation of data; writing of the report; and the decision to submit the report for publication, including whether they will have ultimate authority over any of these activities	N/A
	5d	Composition, roles, and responsibilities of the coordinating centre, steering committee, endpoint adjudication committee, data management team, and other individuals or groups overseeing the trial, if applicable (see Item 21a for data monitoring committee)	P17-19
**Introduction**			
Background and rationale	6a	Description of research question and justification for undertaking the trial, including summary of relevant studies (published and unpublished) examining benefits and harms for each intervention	P5-6
	6b	Explanation for choice of comparators	P6
Objectives	7	Specific objectives or hypotheses	P7
Trial design	8	Description of trial design including type of trial (eg, parallel group, crossover, factorial, single group), allocation ratio, and framework (eg, superiority, equivalence, noninferiority, exploratory)	P7-8

For peer review only

Methods: Participants, interventions, and outcomes

Study setting	9	Description of study settings (eg, community clinic, academic hospital) and list of countries where data will be collected. Reference to where list of study sites can be obtained	P7
Eligibility criteria	10	Inclusion and exclusion criteria for participants. If applicable, eligibility criteria for study centres and individuals who will perform the interventions (eg, surgeons, psychotherapists)	P10-12
Interventions	11a	Interventions for each group with sufficient detail to allow replication, including how and when they will be administered	P13-14
	11b	Criteria for discontinuing or modifying allocated interventions for a given trial participant (eg, drug dose change in response to harms, participant request, or improving/worsening disease)	P14, P15-16
	11c	Strategies to improve adherence to intervention protocols, and any procedures for monitoring adherence (eg, drug tablet return, laboratory tests)	P13
	11d	Relevant concomitant care and interventions that are permitted or prohibited during the trial	P17
Outcomes	12	Primary, secondary, and other outcomes, including the specific measurement variable (eg, systolic blood pressure), analysis metric (eg, change from baseline, final value, time to event), method of aggregation (eg, median, proportion), and time point for each outcome. Explanation of the clinical relevance of chosen efficacy and harm outcomes is strongly recommended	P15-16
Participant timeline	13	Time schedule of enrolment, interventions (including any run-ins and washouts), assessments, and visits for participants. A schematic diagram is highly recommended (see Figure)	P9, Figure1
Sample size	14	Estimated number of participants needed to achieve study objectives and how it was determined, including clinical and statistical assumptions supporting any sample size calculations	P10
Recruitment	15	Strategies for achieving adequate participant enrolment to reach target sample size	P12

Methods: Assignment of interventions (for controlled trials)

Allocation:

Sequence generation	16a	Method of generating the allocation sequence (eg, computer-generated random numbers), and list of any factors for stratification. To reduce predictability of a random sequence, details of any planned restriction (eg, blocking) should be provided in a separate document that is unavailable to those who enrol participants or assign interventions	P12

For peer review only

Allocation concealment mechanism	16b	Mechanism of implementing the allocation sequence (eg, central telephone; sequentially numbered, opaque, sealed envelopes), describing any steps to conceal the sequence until interventions are assigned	P12
Implementation	16c	Who will generate the allocation sequence, who will enrol participants, and who will assign participants to interventions	P12
Blinding (masking)	17a	Who will be blinded after assignment to interventions (eg, trial participants, care providers, outcome assessors, data analysts), and how	P12
	17b	If blinded, circumstances under which unblinding is permissible, and procedure for revealing a participant's allocated intervention during the trial	P12
**Methods: Data collection, management, and analysis**			
Data collection methods	18a	Plans for assessment and collection of outcome, baseline, and other trial data, including any related processes to promote data quality (eg, duplicate measurements, training of assessors) and a description of study instruments (eg, questionnaires, laboratory tests) along with their reliability and validity, if known. Reference to where data collection forms can be found, if not in the protocol	P17-19
	18b	Plans to promote participant retention and complete follow-up, including list of any outcome data to be collected for participants who discontinue or deviate from intervention protocols	P8
Data management	19	Plans for data entry, coding, security, and storage, including any related processes to promote data quality (eg, double data entry; range checks for data values). Reference to where details of data management procedures can be found, if not in the protocol	P17
Statistical methods	20a	Statistical methods for analysing primary and secondary outcomes. Reference to where other details of the statistical analysis plan can be found, if not in the protocol	P17-19
	20b	Methods for any additional analyses (eg, subgroup and adjusted analyses)	P17
	20c	Definition of analysis population relating to protocol non-adherence (eg, as randomised analysis), and any statistical methods to handle missing data (eg, multiple imputation)	P17
**Methods: Monitoring**			
Data monitoring	21a	Composition of data monitoring committee (DMC); summary of its role and reporting structure; statement of whether it is independent from the sponsor and competing interests; and reference to where further details about its charter can be found, if not in the protocol. Alternatively, an explanation of why a DMC is not needed	P19

For peer review only

	21b	Description of any interim analyses and stopping guidelines, including who will have access to these interim results and make the final decision to terminate the trial	P17,19
Harms	22	Plans for collecting, assessing, reporting, and managing solicited and spontaneously reported adverse events and other unintended effects of trial interventions or trial conduct	P15-16
Auditing	23	Frequency and procedures for auditing trial conduct, if any, and whether the process will be independent from investigators and the sponsor	P19
**Ethics and dissemination**			
Research ethics approval	24	Plans for seeking research ethics committee/institutional review board (REC/IRB) approval	P1, P22
Protocol amendments	25	Plans for communicating important protocol modifications (eg, changes to eligibility criteria, outcomes, analyses) to relevant parties (eg, investigators, REC/IRBs, trial participants, trial registries, journals, regulators)	P22-23
Consent or assent	26a	Who will obtain informed consent or assent from potential trial participants or authorised surrogates, and how (see Item 32)	P23
	26b	Additional consent provisions for collection and use of participant data and biological specimens in ancillary studies, if applicable	N/A.
Confidentiality	27	How personal information about potential and enrolled participants will be collected, shared, and maintained in order to protect confidentiality before, during, and after the trial	P23
Declaration of interests	28	Financial and other competing interests for principal investigators for the overall trial and each study site	P29
Access to data	29	Statement of who will have access to the final trial dataset, and disclosure of contractual agreements that limit such access for investigators	P17
Ancillary and post-trial care	30	Provisions, if any, for ancillary and post-trial care, and for compensation to those who suffer harm from trial participation	P23
Dissemination policy	31a	Plans for investigators and sponsor to communicate trial results to participants, healthcare professionals, the public, and other relevant groups (eg, via publication, reporting in results databases, or other data sharing arrangements), including any publication restrictions	P4, P23-24
	31b	Authorship eligibility guidelines and any intended use of professional writers	N/A
	31c	Plans, if any, for granting public access to the full protocol, participant-level dataset, and statistical code	N/A

For peer review only

Appendices

Informed consent materials	32	Model consent form and other related documentation given to participants and authorised surrogates	Attatchment
Biological specimens	33	Plans for collection, laboratory evaluation, and storage of biological specimens for genetic or molecular analysis in the current trial and for future use in ancillary studies, if applicable	P16

*It is strongly recommended that this checklist be read in conjunction with the SPIRIT 2013 Explanation & Elaboration for important clarification on the items. Amendments to the protocol should be tracked and dated. The SPIRIT checklist is copyrighted by the SPIRIT Group under the Creative Commons "Attribution-NonCommercial-NoDerivs 3.0 Unported" license.

For peer review only

BMJ Open

Effect of herbal extract granules combined with octylonium bromide on irritable bowel syndrome with diarrhea: a pilot study protocol for a randomized controlled trial

Journal:	*BMJ Open*
Manuscript ID	bmjopen-2017-018362.R1
Article Type:	Protocol
Date Submitted by the Author:	14-Sep-2017
Complete List of Authors:	Kim, Joong Il; Catholic Kwandong University International Saint Mary's Hospital, Institute for Integrative Medicine;<br>Kim, Pumsoo; Catholic Kwandong University International Saint Mary's Hospital, Institute for Integrative Medicine<br>Lee, Jin-Hyun; Wonkwang University College of Korean Medicine, Department of Korean Rehabilitation Medicine<br>Kim, Yoo-Jin; Catholic Kwandong University International Saint Mary's Hospital, Institute for Integrative Medicine<br>Yang, Na-rae; Catholic Kwandong University International Saint Mary's Hospital, Institute for Integrative Medicine<br>Baeg, Myong Ki; Catholic Kwandong University International Saint Mary's Hospital, Institute for Integrative Medicine<br>Choi, Ja Sung; Catholic Kwandong University International Saint Mary's Hospital, Institute for Integrative Medicine<br>Kim, Hye-Jung; Catholic Kwandong University International Saint Mary's Hospital, Institute for Integrative Medicine<br>Kim, Ja-Young; Catholic Kwandong University International Saint Mary's Hospital, Institute for Integrative Medicine<br>Sunwoo, Yun-Young; Catholic Kwandong University International Saint Mary's Hospital, Institute for Integrative Medicine<br>Lee, Jeong-Han; Wonkwang University College of Korean Medicine, Department of Korean Rehabilitation Medicine<br>Ha, Hyekyung; Korea Institute of Oriental Medicine, K-herb Research Center<br>Park, Tae-Yong; Catholic Kwandong University International Saint Mary's Hospital, Institute for Integrative Medicine
**Primary Subject Heading**:	Complementary medicine
Secondary Subject Heading:	Gastroenterology and hepatology
Keywords:	Samryungbaekchulsan, octylonium bromide, irritable bowel syndrome, randomized controlled study, clinical trial protocol

SCHOLARONE™ Manuscripts

For peer review only

Effect of herbal extract granules combined with octylonium bromide on irritable bowel syndrome with diarrhea: a pilot study protocol for a randomized controlled trial

Joong Il Kim[1*], Pumsoo Kim[1*], Jin-Hyun Lee[2], Yoo-Jin Kim[1], Na-rae Yang[1], Myong Ki Baeg[1], Ja Sung Choi[1], Hye-Jung Kim[1], Ja-Young Kim[1], Yun-Young Sunwoo[1], Jeong-Han Lee[2], Hyekyung Ha[3], Tae-Yong Park[1]

[1] *Institute for Integrative Medicine, Catholic Kwandong University International St. Mary's Hospital, Incheon, 22711, South Korea*

[2] *Department of Korean Rehabilitation Medicine, Wonkwang University College of Korean Medicine, Iksan, 54383, South Korea*

[3] *K-herb Research Center, Korea Institute of Oriental Medicine, Daejeon, 34054, South Korea*

Corresponding author

Tae-Yong Park

Address: Institute for Integrative Medicine, Catholic Kwandong University International, St. Mary's Hospital, Simgok-ro 100gil 25, Seo-gu, Incheon, 22711, Republic of Korea

E-mail: parktae9822@gmail.com

Telephone: +82-32-290-3319

*** These authors contributed equally to the study**

Funding: This work was supported by a "Construction of Scientific Evidences for Herbal Medicine Formulas (K15250)" grant from the Korea Institute of Oriental Medicine (KIOM). (Telephone: +82-42-868-9513)

Word Count: 5829

For peer review only - http://bmjopen.bmj.com/site/about/guidelines.xhtml

Keywords: Samryungbaekchulsan, octylonium bromide, irritable bowel syndrome, randomized controlled study, clinical trial protocol

For peer review only

For peer review only - http://bmjopen.bmj.com/site/about/guidelines.xhtml

ABSTRACT

Introduction

Irritable bowel syndrome (IBS), known as a functional and organic gastrointestinal (GI) disorder, is a collection of symptoms that occur together and generally include pain or discomfort in the abdomen and changes in bowel movement patterns. Due to the limitations of conventional treatments, alternative IBS treatments are used by many patients worldwide. Samryungbaekchulsan (SRS), a herbal formula, has long been used for alleviating diarrhea-predominant IBS (D-IBS) in traditional Korean medicine. Octylonium bromide (OB) is an antimuscarinic compound used to relieve spasmodic pain in the gut, especially in IBS. Although herbal formulas and western drugs are commonly co-administered for various diseases in Korea, few clinical studies have been conducted regarding the synergic effects of these treatments for any disease, including D-IBS.

Methods and Analysis

This trial is a randomized, double-blinded, placebo-controlled, double-dummy, four-arm, parallel study. After a 2-week preparation period, 80 patients with D-IBS will be randomly assigned to one of four treatment groups consisting of SRS (water extract granules, 5 g/pack, 3 times a day) with OB (tablet form, 1 capsule 3 times a day) or their placebos, with treatment lasting for 8 weeks. Post-treatment follow-up will be conducted 4 weeks after the end of treatment. The primary outcome is the finding obtained using the Subject's Global Assessment of Relief method. The secondary outcomes are the severity of symptoms related to D-IBS, determined using a 10-point scale, and the change in symptoms.

Ethics and dissemination

This trial has full ethical approval of the ethics committee of Catholic Kwandong University International St. Mary's Hospital (IS15MISV0033) and the Korean Ministry of Food and Drug Safety (30769). The results of the study will be disseminated through a peer-reviewed journal and/or conference presentations.

For peer review only - http://bmjopen.bmj.com/site/about/guidelines.xhtml

Trial protocol version: IS15MISV0033 Version 4.0 (July 25, 2016)

Trial registration number: KCT0001621 (Approval date: Aug 10, 2015)

STRENGTHS AND LIMITATIONS OF THIS STUDY

■ In Korea, herbal and western medicine have been co-administered without sufficient evidence for various diseases; however, this study will provide basic evidence for such co-administration and will contribute to the development of integrative medicine.

■This randomized controlled trial is the first Korean clinical trial approved by the Korean Ministry of Food and Drug Safety (KMFDS) to investigate the efficacy and safety of Samryungbaekchulsan (SRS; herbal formula) and octylonium bromide (OB; western drug) in diarrhea-predominant irritable bowel syndrome (D-IBS).

■The primary outcome is the improvement in the patient's symptoms according to the Subject's Global Assessment of Relief and the secondary outcome is the severity of D-IBS symptoms.

■This study is conducted with a small sample size (four arms, 20 participants per group) because it is a pilot study and further clinical trials with powerful sample sizes will be required to more adequately determine the effectiveness of SRS and/or OB for D-IBS.

For peer review only

For peer review only - http://bmjopen.bmj.com/site/about/guidelines.xhtml

INTRODUCTION

Irritable bowel syndrome (IBS), a chronic gastrointestinal (GI) disease and functional bowel disorder, is characterized by abnormal bowel habits and abdominal pain without structural, morphological, and histological abnormalities. It affects approximately 20% of the population worldwide, according to population-based studies[1-6]. In addition, recent evidence suggests that IBS is not only a functional disease, but also an organic disease with a complex of symptoms, including infection, immune activation, serotonin dysregulation, bacterial overgrowth, central dysregulation, brain-gut interaction, and genetics[4-6]. Based on their predominant bowel habits, patients with IBS can be divided into diarrhea-predominant IBS (D-IBS), constipation-predominant IBS, or mixed IBS[6 7]. Recently, smooth-muscle relaxants, bulking agents, and anti-diarrheal agents have been used in the treatment of IBS[8]. However, these methods are often not effective and many IBS patients also use complementary treatments, such as herbal medicine, acupuncture, psychological treatment, and lifestyle correction[3 9 10].

Samryungbaekchulsan (SRS; Shenlingbaizhu-san in Traditional Chinese Medicine; Jinryobyakujutsu-san in Kampo Medicine), which was introduced in the famous classical compendium of herbal formulas “Formulary of Peaceful Benevolent Dispensary,” consists of 10 herbs (Atractylodes rhizoma Alba, Poria sclerotium, Dioscoreae rhizoma, Glycyrrhizae radix et rhizoma, Coicis semen, Nelumbinis semen, Platycodonis radix, Dolichoris semen, Amomi fructus, and Ginseng radix). In traditional Korean and Chinese medicine, this herbal formula has been used to treat GI disease, and various studies have been published regarding the treatment of IBS with SRS[11-13]. SRS has been shown to have anti-acetylcholine and anti-barium chloride effects and to suppress gastric secretion and small intestine motility[14]. However, no clinical trials have evaluated the efficacy of SRS for IBS when co-administered with a conventional drug.

For peer review only
For peer review only - http://bmjopen.bmj.com/site/about/guidelines.xhtml

Octylonium bromide (OB) is widely used for the prevention and reduction of symptoms in patients with IBS[15]. OB has been shown to inhibit L- and T-type calcium channels, muscarinic receptors, and tachykininergic responses in human cultured smooth muscle cells and rat colon strips[16]. According to a recent randomized controlled trial (RCT), OB (40 and 80 mg) is more effective for treating IBS than placebo, not only for the reduction of bloating and abdominal pain, but also for protection from relapse owing to its long-lasting effect[17 18].

According to the guidelines of the Korean Society of Gastroenterology, antispasmodic agents are recommended for the treatment of D-IBS (Grade 1B),[19] and SRS is the preferred drug for diarrhea treatment in Korean medicine[20]. Recently, several studies have been conducted to evaluate the therapeutic effect of the co-administration of herbal formulas and conventional drugs for IBS in China[21 22]. However, there has been no such clinical study in Korea, even though both herbal medicine and conventional drugs are widely used for IBS treatment in the clinical field. Therefore, there is a need to evaluate the efficacy and safety of SRS, OB, and the co-administration of both drugs.

In this study, we expect that the combined administration of OB and SRS will result in synergistic effects that improve D-IBS symptoms, including abdominal pain, discomfort (the main effect of OB), and diarrhea-like stool patterns (the main effect of SRS).

For peer review only - http://bmjopen.bmj.com/site/about/guidelines.xhtml

METHODS AND ANALYSIS

Objectives

The purpose of this study is to evaluate the efficacy and safety of the co-administration of SRS and OB to patients with D-IBS.

Necessity of the trial

Recently, herbal medicines and western drugs have been co-administered for various medical therapies. This is particularly true in Korea, where approximately 76% of patients have used the combination of herbal medicines and western drugs[23]. However, only a few studies have evaluated the interaction between western and herbal medicines. Despite the need for combined treatment in patients with various diseases, there are no standard clinical guidelines or scientific evidence regarding co-administration because of a lack of research and data.

D-IBS is a disease that is difficult to cure and many patients require treatment with both western and herbal drugs to experience an improvement in their symptoms. Therefore, it is very important to conduct a clinical study regarding the efficacy and safety of the combined administration of SRS and OB in patients with D-IBS.

Basis of drug selection

According to guidelines for IBS treatment, the OB recommendation level is high (i.e., the level of evidence is moderate and many experts recommend using it) and it is the most reasonable treatment option[21]. SRS is the herbal formula used most often by clinical Korean and Chinese medical doctors for abdominal pain, abdominal discomfort, and chronic diarrhea, which are typical symptoms of D-IBS[11-13 19]. Based on these studies and clinical applications, OB and SRS were selected for this trial.

For peer review only - http://bmjopen.bmj.com/site/about/guidelines.xhtml

Hypothesis

We hypothesize the following: (1) that co-administration of SRS and OB will have a more positive effect on D-IBS symptoms than either SRS or OB alone; (2) that after 8 weeks of treatment with SRS, OB, or both drugs, the Subject's Global Assessment of Relief and severity of D-IBS symptoms will be more improved than after treatment with placebo; and (3) that co-administration of SRS and OB will be safe for patients with D-IBS.

Design

This study is a placebo-controlled, double-blind trial. Eighty patients will be enrolled and randomly assigned to four separate arms of the trial. The trial will be conducted at Catholic Kwandong University International St. Mary's Hospital, Incheon, South Korea.

The study subjects will be required to participate in a 2-week preparation period (weeks -2 to 0) and an 8-week drug administration period (weeks 0 to 8). The patients will visit four times, on weeks 0, 4, 8, and 12. The primary outcome measurement will be evaluated at weeks 2, 4, 6, 8, and 12, and the secondary outcome measurement will be evaluated at weeks 0, 2, 4, 6, 8, and 12. Participants will be assessed for efficacy and safety of the treatment through visits (0, 4, 8, 12 weeks) and telephone questionnaires (2, 6 weeks). In addition, lab tests will be conducted at 0, 4, and 8 weeks. According to the intervention protocols, the clinical research coordinator (CRC) will encourage patients and participants to complete follow-up at every visit.

During the drug administration period, SRS or its placebo will be given as 1 pack of water extract granules 3 times a day, 30 min before each meal. OB or its placebo will be given as 1 tablet 3 times a day, 30 min before each meal.

Participants will be divided into 4 groups: (1) SRS and OB group; (2) SRS and placebo-

For peer review only - http://bmjopen.bmj.com/site/about/guidelines.xhtml

OB group; (3) placebo-SRS and OB group; and (4) placebo-SRS and placebo-OB group. The entire flow chart of the study is shown in Figure 1.

The protocol of this trial has been approved by the institutional review board (IRB) and ethics committee of Catholic Kwandong University International St. Mary's Hospital. The IRB permission number is IS15MISV0033 and CRIS (Clinical Research Information Service, www.cris.nih.go.kr) protocol identification number is KCT0001621. In addition, this study received the first approval given by the Korean Ministry of Food and Drug Safety (KMFDS) for a study of the co-administration of a conventional drug and a Korean traditional herbal medicine (approval number: 30769).

Potential participants will be provided with sufficient information about the study and only those who agree to the study protocol and sign the consent form will be enrolled. The study will be implemented according to Good Clinical Practice and the revised version of the Declaration of Helsinki.

Sample size calculation

This trial is a pilot study for a new therapeutic regimen of SRS and OB combination treatment. In a similar study conducted previously by using co-administration of a probiotics mixture and an herbal medicine (placebo-controlled, double-blind, and randomly 4-arm), each arm consisted of at least 15 patients and the total sample size was more than 60 participants[24]. In other similar pilot studies, clinical trials have been conducted with a total of 60–64 participants, consisting of 12–30 patients per group[25-27]. Based on these previous studies, the minimal clinical significance of the trial can be obtained when only 16 participants complete the trial. Because the drop-out rate is expected to be 20%, each trial group will be made up of at least 20 participants. Therefore, the total sample size must be more than 80 participants.

For peer review only - http://bmjopen.bmj.com/site/about/guidelines.xhtml

Inclusion criteria

The following patients would be included: (1) patients aged 18–75 years; (2) patients satisfying the Rome III criteria of IBS (recurrent abdominal pain or discomfort that began at least 6 months before the clinical trial, with a rating of more than 3 on a 10-point scale, frequency of at least 3 days/month in the last 3 months, and associated with two or more of the following: improvement associated with defecation, onset associated with a change in frequency of stools, and onset associated with a change in form of stool)[2 7]; (3) patients satisfying the Rome III criteria of IBS, whereby D-IBS type is defined as loose/mushy or watery stools—Bristol Stool Form (BSF) Scale 6-7 ≥ 2 times/week[2 27]; (4) patients with a negative urine pregnancy test (in women of a childbearing age, within 7 days before the clinical trial) and who consent to using contraception during the administration period; (5) patients with the ability to read the symptom questionnaire and understand it; and (6) patients who agree with the clinical plan and voluntarily sign the IRB-approved documents.

Exclusion criteria

The exclusion criteria are as follows: (1) chronic liver disease (cirrhosis, chronic hepatitis B or C); (2) chronic renal failure or renal impairment (serum creatinine ≥ 2 times the upper limit of normal); (3) liver dysfunction (AST/ALT ≥ 3 times the upper limit of normal); (4) diabetes (HbA1c > 8% or not controlled by diet or medication), hypertension (≥ 160/100 mmHg), thyroid dysfunction (exceptions in the following cases: if the disease is controlled by drug administration with a stable dose for 12 weeks before the screening, and the drug dose remains constant during the trial period), clinically significant hematologic, cardiac, pulmonary, neurological disorders, or other severe systemic disorders; (5) abnormal findings on colonoscopy or colonography within the last 5 years; (6) history of surgery that affects

For peer review only - http://bmjopen.bmj.com/site/about/guidelines.xhtml

gastrointestinal motility (i.e., gastrectomy, colonic resection, hysterectomy, except for appendectomy); (7) GI disease characterized by the following symptoms within 6 months before the clinical trial: inflammation of or ulcer in the esophagus, stomach, or duodenum; gastroesophageal reflux disease (at the discretion of the examiner, subjects may enroll if the disease is chronic and not acute); gastrointestinal bleeding; gastrointestinal stenosis or closure; infectious diarrhea; inflammatory bowel disease (i.e., Crohn's disease, diverticulitis, ulcerative colitis, infectious enteritis, ischemic colitis); pancreatic insufficiency; biliary abscess; (8) mental illness or an addiction to drugs or alcohol; (9) severe systemic organ diseases such as cancer, autoimmune disease, stroke (although patients in remission for more than 5 years from a cancer unrelated to the GI tract can participate in the trial); (10) pregnancy, breastfeeding, and unwillingness to use contraception during the trial; (11) a history of taking the following drugs within 2 weeks before the trial: antibiotics, analgesics, antidepressants, anti-anxiety agents, anti-inflammatory agents, anti-ulcer agents, anti-gastric secretion inhibitors, laxatives, antispasmodics, antacid agents, gastrointestinal stimulants, prostaglandin agents, and corticosteroids; (12) use of medications that do not match the intent of this trial or have a clinical interaction with SRS or OB; (13) lactose intolerance (not controlled by food); (14) glaucoma; (15) participation in other clinical studies within 30 days prior to clinical screening and have received other clinical trial medications (including placebo); (16) hypersensitivity to clinical trial medicines; and (17) other reasons considered inappropriate for participation in clinical trials.

Recruitment, randomization, blinding, and unblinding

Advertisements for the study will be posted at the Catholic Kwandong University International St. Mary’s Hospital using banner ads and posters. We also plan to post the advertisements on the hospital homepage, in the monthly hospital magazine, at the internet

For peer review only - http://bmjopen.bmj.com/site/about/guidelines.xhtml

café, and on the subway at least five times. All these advertisements will be done with the approval of the IRB.

After recruitment, patients will be randomized to one of the four trial arms. Randomization will be performed using a list of block randomizations made by statisticians, independent of this clinical trial. A researcher who is not involved in process of drug prescription and evaluation will execute the randomization process.

During the treatment period, the patients and all researchers (investigators and clinical research coordinators) will be blinded, except the researchers who conducted the randomization procedure. In addition, the researchers will not know the kind of medication that is being administered to the patient or any other information that could lead to bias.

Allocation concealment will be performed during the process of medication administration. The management pharmacist, who will not be involved in analyzing the results of the trial, will use the randomization table to label the medication with the assignment number and provide the medication. The patients will take the drugs contained in the opaque envelope, which will be labeled with the assignment number.

Unblinding will be performed only in the following situations: (1) if the trial is terminated and there is a need for statistical analysis; (2) if a serious medical emergency occurs and information about the medication is needed; and (3) if the chief investigator determines that unblinding is necessary.

The entire process of the study will be directed by the authorized clinical research organization (CRO), Dream CIS Corporation, Seoul, Korea.

Intervention

The herbal formula SRS is commonly used by Korean traditional medical doctors for treatment of diarrhea and related diseases[11-13] [16]. SRS used in this study

For peer review only - http://bmjopen.bmj.com/site/about/guidelines.xhtml

(Samryungbaekchulsan granule®, Hankook shin yak, Co., Ltd., Nonsan, Korea) is extracted with water and mixed with starch and lactose in accordance with Korean Good Manufacturing Practice (K-GMP). Production of SRS is regulated and allowed by the KMFDS. SRS contains 10 herbs, listed in Table 1. Placebo-SRS is made from cornstarch and has the same taste, shape, color, and similar scent as SRS. SRS and placebo-SRS will be sealed in identical aluminum bags with the same labeling. These herbal drug packages will be distributed by an independent pharmacist in an isolated room. According to the recommended dosing methods of KMFDS, after SRS or placebo-SRS is dissolved in boiled water, the subjects will take them 30 min before each meal (5 g/pack, 3 times a day).

OB (Menoctyl Tab®, Dong Hwa Pharm Co., LTD., Seoul, Korea) is a tablet containing octylonium bromide 40 mg. Placebo-OB is a tablet made of Ludipress with the same taste, shape, and color as OB. Patients will be instructed to swallow one tablet of OB or placebo-OB 30 min before each meal over the administration period. The drugs used in the trial (SRS, OB, placebo-SRS, placebo-OB) have been approved by the KMFDS. Patients will be asked at the end of the study whether the drugs that they have been taking are either real or placebo, to measure the success of blinding. Compliance will be confirmed by counting returned SRS packets or OB tablets. Every patient will be instructed to write down any adverse events during the administration period and these records will be evaluated at the follow-up. At the patient visits (at 0, 4, and 8 weeks), we will perform a check of vital signs, physical examination, electrocardiogram, and laboratory tests to evaluate safety for the patients. All side effects will be reported to the investigator.

For peer review only

For peer review only - http://bmjopen.bmj.com/site/about/guidelines.xhtml

Table. 1 Ingredients in the herbal formula Samryungbaekchulsan (SRS)

Scientific name	Part used	Grams/day
Atractylodis Rhizoma Alba	Root	3.0
Poria Sclerotium	Dried core	4.0
Rhizoma Dioscoreae	Root	3.0
Glycyrrhizae Radix et Rhizoma	Root	1.5
Semen Coicis	Seed	8.0
Semen Nelumbinis	Fruit	4.0
Platycodonis Radix	Root	2.5
Dolichoris Semen	Seed	4.0
Amomi Fructus	Fruit	2.0
Ginseng Radix	Root	3.0

Rescue therapy and concomitant medications

Participants are prohibited from taking any medication that could affect D-IBS during the entire course of the trial. However, if serious side effects occur or symptoms worsen, investigators will provide quick and appropriate treatment (including drug administration), based on their medical judgement. In this case, the direct investigators will record the treatment or drug administration and report to the IRB within 24 h to determine whether the trial should be continued or discontinued.

Outcome Measurements

Primary outcome

The primary outcome will be measured via a questionnaire that confirms subjective

For peer review only

symptom improvement. Participants will respond a total of five times during and after the administration period (at 2 and 6 weeks via telephone, and at 4, 8, and 12 weeks during direct visits) to the following question: Subject's Global Assessment of Relief; "How much do you think the symptoms of D-IBS have improved compared to before the clinical trial?" According to the answers given by the patients to this survey, the improvement is given a score of 0, 1, 2, 3, or 4.

Secondary outcomes

At 2, 4, 6, 8, and 12 weeks from the start of the treatment period, patients will be assessed for severity of D-IBS symptoms (abdominal pain, abdominal discomfort, satisfaction of defecation, frequency of abdominal pain, quality of life) using a Likert scale ranging from 0 to 10[28]. Patients will also be instructed to record the number of defecations per day, BSF, and the degree of force used in bowel movements at 0, 2, 4, 6, 8, and 12 weeks[29].

Safety assessments

The investigator will record all adverse events that occur during the trial along with any concomitant medications in the case reports. When an adverse event occurs, the investigator will record the symptoms and signs of the adverse reaction, the duration (start and end date), severity, course, outcome, significance, causality by the trial drug, and any action taken in relation to the adverse event. In the case of concomitant medicines, composition, dosage, duration of administration, and reason for medication will be recorded in detail. Symptoms that existed before the start of the clinical trial will not be recorded as adverse events.

Laboratory tests

The purpose of laboratory tests in this study is to evaluate the safety and pharmacokinetic

For peer review only - http://bmjopen.bmj.com/site/about/guidelines.xhtml

profiles of SRS and OB. Because of the small number of patients assigned to this study and the different baseline values for each individual patient, we will not perform a detailed statistical analysis. However, if significant abnormal results are found in the laboratory tests, the clinical laboratory results will be described and the connection to the drug will be considered.

Blood tests will be conducted at 0, 2, 4, and 8 weeks and urine tests will be performed at 0 and 4 weeks during the clinical trial. In the case of a participant who drops out or the early termination of the clinical trial, blood and urine tests will be performed on the last day of the trial.

Using the test results (comprehensive verification of vital signs, laboratory tests, electrocardiograms, and physical examinations) for each individual patient, the investigator will determine adverse reactions caused by the drugs and statistical tests will be conducted as needed on clinically significant parameters.

Test of human derivatives

IBS is known to be closely related to stress[10]. Therefore, we plan to quantify factors associated with stress or that have been reported to worsen IBS symptoms as follows: 1) cortisol; 2) corticotropin-releasing hormone (CRH); 3) serotonin; 4) group I cytokines (10 species); 5) group II cytokines (14 species); and 6) growth factors (3 species)[30 31]. Serum will be separated from blood specimens collected at 0, 4, 8 weeks, stored frozen, and transferred to the Korea Institute of Oriental Medicine (KIOM) for examination.

Early termination or dropout

The criteria for early termination or dropout are as follows: 1) administration of other drugs that are expected to affect the safety and efficacy of the clinical trial drugs; 2) request

For peer review only - http://bmjopen.bmj.com/site/about/guidelines.xhtml

of participants to discontinue the clinical trial, or withdrawal of the trial agreement; 3) occurrence of significant adverse drug reactions or events that preclude continuing the trial, according to the judgment of the clinical investigator; 4) new discovery of violations of significant clinical trial protocol during clinical trial; 5) less than 70% trial drug compliance; and 6) any other reason that the clinical trial should be discontinued at the discretion of the clinical trial manager or investigator.

Data collection, access, and management

The data of this trial will be managed according to the standard work instructions of Catholic Kwandong University International, St. Mary's Hospital. Other contents not specified in the trial protocol shall follow the standards of The International Conference on Harmonization of Technical Requirements for Registration of Pharmaceuticals for Human Use (ICH) Guideline for Good Clinical Practice (GCP) and Korean Good Clinical Practice (KGCP) guideline.

The source document is recorded immediately when the data are collected. After the source document input is completed, it will be recorded in the case record form (CRF). All the documents will be kept safe, so that the data can be verified by the relevant government agencies and the IRB. Only research colleagues and others who have been approved by the principle investigator will have access to all data obtained from this trial.

Statistical analysis

The statistical analysis will be conducted by blinded professional statisticians. To evaluate the efficacy of the study, both an intent-to-treat (ITT) analysis (primary analytical method) and a per-protocol (PP) analysis (secondary analytical method) will be performed. The ITT analysis will be conducted for all patients who were followed up at least once after

For peer review only - http://bmjopen.bmj.com/site/about/guidelines.xhtml

randomization and will be able to be evaluated for effectiveness. The PP analysis will be performed for patients who complete the 8-week treatment course and meet the inclusion and exclusion criteria.

To analyze the continuous variables in the demographic information and pre-treatment characteristics between groups, a one-way analysis of variance (ANOVA) and Kruskal-Wallis test will be conducted. An ANOVA and Kruskal-Wallis test will also be used to test for symptom improvement compared to baseline (0 week) and the variables for analysis that include the following: (1) subjective symptom improvement (primary outcomes); and (2) severity of symptoms related to D-IBS (abdominal pain, abdominal discomfort, satisfaction of defecation, frequency of abdominal pain, quality of life). All values will be shown as mean ± standard error of the mean (SEM). Comparisons of outcome assessments between the four groups will be performed using the paired t-test or Wilcoxon signed-rank test.

If the Subject's Global Assessment of Relief on the Likert scale is "0" or "1" for more than 2 weeks between weeks 8 and 12, then the symptoms will be considered to have improved sufficiently. Based on this, we will calculate the symptom relief rate by dividing the number of participants who exhibited sufficient symptom relief by the total number of participants in the trial and present descriptive statistics. The CATMOD procedure will be used to evaluate the relief rate improvement of symptoms compared with baseline. Comparisons between the four groups will be conducted using the chi-square test or Fisher's exact test. These statistical analyses will also be performed at 2, 4, 6, 8, and 10 weeks from baseline. The Cochran-Mantel-Haenszel (CMH) test will be used to confirm the difference between the intervention groups at each time-point.

A statistical analysis will also be conducted to evaluate safety. The incidence of adverse events and abnormality of experimental results will be analyzed statistically according to the groups and non-parametric methods will be applied as necessary using a paired t-test for

For peer review only - http://bmjopen.bmj.com/site/about/guidelines.xhtml

continuous data, McNemar's test for categorical data, Fisher's exact test for adverse events, and a generalized estimating equation for clinically significant changes between groups.

All statistical analysis will be conducted using SAS software, version 9.1.3 (SAS institute, Cary, NC). A P-value < 0.05 will be considered statistically significant.

Quality control and data monitoring

To ensure the results and quality of the clinical trial, assessments and monitoring will be performed by the CRO, DreamCIS Corporation (Seoul, Korea), which is independent from the sponsor and competing interests. During the trial, the CRO will regularly monitor whether the study is proceeding in accordance with the protocol through the use of all related documentation, including trial master files, case report forms, informed consent forms, and adverse event reports.

DISCUSSION

Herbal medicine is a recognized effective treatment method based on clinical experience accumulated over thousands of years and more recently gained attention as an alternative therapy for treating various diseases that are difficult to treat or have unknown causes, such as IBS[8 22]. SRS is frequently used for abdominal pain and diarrhea in Korean and Chinese traditional medicine. According to experimental research, SRS has an anti-cholinergic effect that improves intestinal digestion and absorption and inhibits intestinal movement, resulting in the prevention of diarrhea and abdominal pain[14]. In addition, clinical studies have shown that the use of SRS in patients with D-IBS is effective for improving diarrhea[32 33]. Some studies have reported that co-administration with SRS and trimebutine maleate or SRS and paroxetine is more effective than trimebutine maleate alone for the improvement in IBS symptoms[11 12 21]. Other studies have reported that SRS exerted more beneficial effects on IBS

For peer review only - http://bmjopen.bmj.com/site/about/guidelines.xhtml

patients compared with Smecta or probiotics[13 33].

However, although there is a growing interest in the use of herbal medicines such as SRS in IBS and related studies are continuously being published, there have been only a few well-designed RCTs. To overcome the limitations of the previous studies, this trial was designed as a double-blind, double-dummy, four-arm, parallel-group RCT. The efficacy of SRS will be evaluated via the Subject's Global Assessment of Relief and the improvement in symptoms associated with bowel movements and abdominal pain. The results of these assessments are expected to provide distinct clinical evidence for the use of SRS in D-IBS treatment.

OB is a representative conventional medicine used for D-IBS treatment[15]. It is a spasmolytic agent that exerts its activity primarily in the distal GI tract through the inhibition of Ca++ flux and direct activation of contractile proteins in the smooth muscle. OB has also been shown to reduce hyper-motility and modulate visceral sensation, the effect of which is to improve D-IBS symptoms[34 35]. RCTs using OB for the treatment of D-IBS have been conducted. In these studies, treatment with OB resulted in a greater improvement in symptoms related to D-IBS (defecation frequency, regular intestinal habits, reductions in frequency of diarrhea, abdominal pain and discomfort, severity of abdominal bloating) and better protection from symptom relapse compared to placebo[17 18 36].

IBS is a disease with a high rate of progression and frequent recurrence. For this reason, patients with IBS often require a variety of treatment methods[3]. Among these methods, the combination of herbal and conventional drugs is widely used and is more effective for improving IBS symptoms than conventional drugs alone[3 37]. However, there are several limitations to the co-administration of herbal and conventional drugs in IBS. Specifically, there is a high risk of bias in related studies and there are insufficient clinical guidelines regarding the interaction of herbal and conventional drugs in D-IBS, including SRS and OB. Therefore, it is necessary to perform well-designed RCTs to evaluate the co-administration of

For peer review only - http://bmjopen.bmj.com/site/about/guidelines.xhtml

herbal and conventional drugs.

This RCT has several characteristic features. First, this trial will be the first to investigate the efficacy of SRS and OB combination therapy for D-IBS. The results of previous studies suggested that the combination of OB and SRS will improve two major D-IBS symptoms at the same time: abdominal pain and discomfort (major effect of OB) and diarrhea (main effect of SRS). This study will assess this hypothesis and provide a basis for the use of SRS and OB combination therapy for D-IBS. Second, the participants in this trial will be grouped into four arms, making it possible to compare the efficacy of combined treatment with the efficacy of each individual treatment. Third, this trial will evaluate the safety of the subjects using several assessments, including a periodic assessment of subjective symptoms, physical measurements, and laboratory testing. The results of the safety analysis may provide evidence for the safety of treatment with the combination of herbal and conventional drugs in D-IBS. Fourth, this trial was the first clinical study related to combination treatment with western and traditional herbal medicine in Korea to receive approval from the KMFDS. Through the systematic management of the trial under the auspices of the KMFDS, a high quality trial will be performed and the results of this study will provide information for use in further clinical applications.

Although our study is a pilot study with a small sample size, it has a creative and systematic research design and the results will be useful basic clinical evidence in the field of integrated herbal and conventional drugs for the management of D-IBS.

Trial status

The research plan and design began in April 2015.

The study received the IRB's final approval after two deliberations. The first submission date was July 01, 2015; the result was a request for revision and supplementation, which was

For peer review only - http://bmjopen.bmj.com/site/about/guidelines.xhtml

received on July 13, 2015. We filed an application for re-review on August 06, 2015, supplemented with the relevant information, and received the IRB's final approval on August 10, 2015. We also requested that the KMFDS review the trial plan on September 14, 2015 and received approval for the clinical research on December 21, 2015.

The first participant was randomized on March 29, 2016. Recruitment for the study is ongoing. The primary completion date for this trial is anticipated to be December 31, 2017, and the study completion date is expected to be February 28, 2018.

Ethics

Research ethics approval

This trial was approved by the IRB and ethics committee of Catholic Kwandong University International St. Mary's Hospital (approval number IS15MISV0033) and the KMFDS (approval number 30769).

Protocol amendments

An investigator who wishes to change the protocol of the clinical trial plan should first discuss it with the trial director. Thereafter, the examiner should obtain prior approval from the IRB for changes to the protocol (except to prevent immediate injury to the patient, in which case it will be reported to the IRB later). However, when a dangerous situation occurs and immediate treatment is needed, the investigator may report the protocol change to the IRB at a later time.

Consent

Patients recruited through the announcement will receive a full explanation of the details of the trial in an easy-to-understand manner. The patients and investigators will have a mutual

For peer review only - http://bmjopen.bmj.com/site/about/guidelines.xhtml

question and answer time before the patient is asked to sign the clinical trial consent. Through these processes, if the patient agrees to participate in the trial, a signed consent form will be obtained.

Confidentiality

All patient information will be anonymized with initials or symbols and all related investigators will keep the trial results confidential. The trial director will keep the signed consent forms and prepare a list that will be used to confirm the patient identities.

Post-trial care

If the patients experience unexpected accidents or injuries, appropriate compensation will be made by the Federal Insurance Company Korea (12th Floor Ferrum Tower 66 Suha-Dong Jung-gu Seoul, 100210, South Korea), according to the patient compensation rules of the trial. Additionally, in the case of an emergency, the patients will receive appropriate medical care at the Catholic Kwandong University International, St. Mary's Hospital.

Dissemination

The results of the trial will be disseminated through a peer-reviewed journal and/or conference presentations.

Contributors

Joong Il Kim, Pumsoo Kim, Jin-Hyun Lee, and Jung-Han Lee contributed to the protocol design and writing of the manuscript. Hyekyung Ha is responsible for monitoring the process of the trial. Na-rae Yang, Yoo-Jin Kim, Myong Ki Baeg, Ja Sung Choi, Hye-Jung Kim, and Ja-Young Kim provide advice on the process of the trial. Tae-Yong Park is responsible for

For peer review only - http://bmjopen.bmj.com/site/about/guidelines.xhtml

writing the manuscript and also managing and supervising the clinical research. All authors approved the final manuscript.

Funding

This work was supported by a "Construction of Scientific Evidences for Herbal Medicine Formulas (K15250)" grant from the Korea Institute of Oriental Medicine (KIOM). (Telephone: +82-42-868-9513)

Competing interests

The authors have no competing interests to declare.

Ethics approval

This trial has been approved by the IRB and ethics committee of Catholic Kwandong University International St. Mary's Hospital (approval number IS15MISV0033).

Provenance and peer review

Not commissioned; externally peer reviewed.

Open Access

This is an Open Access article distributed in accordance with the Creative Commons Attribution Non-Commercial (CC BY-NC 4.0) license, which permits others to distribute, remix, adapt, build upon this work non-commercially, and license their derivative works on different terms, provided the original work is properly cited and the use is non-commercial. See: http://creativecommons.org/licenses/by-nc/4.0/

For peer review only - http://bmjopen.bmj.com/site/about/guidelines.xhtml

© Article author(s) (or their employer(s) unless otherwise stated in the text of the article) 2017. All rights reserved. No commercial use is permitted unless otherwise expressly granted.

For peer review only

REFERENCES

1. Maxion-Bergemann S, Thielecke F, Abel F, et al. Costs of irritable bowel syndrome in the UK and US. *Pharmacoeconomics* 2006;24(1):21-37.

2. Drossman DA. Introduction. The Rome Foundation and Rome III. *Neurogastroenterol Motil* 2007;19(10):783-6. doi: 10.1111/j.1365-2982.2007.01001.x

3. Grundmann O, Yoon SL. Irritable bowel syndrome: epidemiology, diagnosis and treatment: an update for health-care practitioners. *J Gastroenterol Hepatol* 2010;25(4):691-9. doi: 10.1111/j.1440-1746.2009.06120.x

4. Soares RL. Irritable bowel syndrome: a clinical review. *World J Gastroenterol* 2014;20(34):12144-60. doi: 10.3748/wjg.v20.i34.12144

5. Saha L. Irritable bowel syndrome: pathogenesis, diagnosis, treatment, and evidence-based medicine. *World J Gastroenterol* 2014;20(22):6759-73. doi: 10.3748/wjg.v20.i22.6759

6. Chey WD, Kurlander J, Eswaran S. Irritable bowel syndrome: a clinical review. *JAMA* 2015;313(9):949-58. doi: 10.1001/jama.2015.0954

7. Longstreth GF, Thompson WG, Chey WD, et al. Functional bowel disorders. *Gastroenterology* 2006;130(5):1480-91. doi: 10.1053/j.gastro.2005.11.061

8. Jailwala J, Imperiale TF, Kroenke K. Pharmacologic treatment of the irritable bowel syndrome: a systematic review of randomized, controlled trials. *Ann Intern Med* 2000;133(2):136-47.

9. Liu JP, Yang M, Liu YX, et al. Herbal medicines for treatment of irritable bowel syndrome. *Cochrane Database Syst Rev* 2006(1):CD004116. doi: 10.1002/14651858.CD004116.pub2

10. Palsson OS, Drossman DA. Psychiatric and psychological dysfunction in irritable bowel syndrome and the role of psychological treatments. *Gastroenterol Clin North Am*

For peer review only - http://bmjopen.bmj.com/site/about/guidelines.xhtml

2005;34(2):281-303. doi: 10.1016/j.gtc.2005.02.004

11. Duan W. Observation of sixty-five cases on Irritable Bowel Syndrome treated by Trimebutine and Shenlingbaizhu-san granule [Chinese]. *Journal of Practical Traditional Chinese Medicine* 2010;26(5):320.

12. Chen Y. Clinical observation of irritable bowel syndrome treated by shenlingbaizhu-san granule and trimebutine [Chinese]. *Journal of Practical Traditional Chinese Medicine* 2011;27(5):318-9.

13. Li Z, Jiang J. Clinical observation of 80 cases on diarrhea - predominant irritable bowel syndrome treated by Shenlingbaizhu-san granule [Chinese]. *Guiding Journal of Traditional Chinese Medicine and Pharmacy* 2013;20(5):41-2.

14. Song DS, Ryu BH, Park DW, et al. Experimental studies on the efficacy of Samryungbaekchulsan. *J Korean Med* 1993;14(1):179-95.

15. Boeckxstaens G, Clave P, Corazziari ES, et al. Irritable bowel syndrome: focus on otilonium bromide. *Expert Rev Gastroenterol Hepatol* 2014;8(2):131-7. doi: 10.1586/17474124.2014.869477

16. Martinez-Cutillas M, Gil V, Gallego D, et al. Mechanisms of action of otilonium bromide (OB) in human cultured smooth muscle cells and rat colonic strips. *Neurogastroenterol Motil* 2013;25(12):e803-12. doi: 10.1111/nmo.12206

17. Chmielewska-Wilkon D, Reggiardo G, Egan CG. Otilonium bromide in irritable bowel syndrome: a dose-ranging randomized double-blind placebo-controlled trial. *World J Gastroenterol* 2014;20(34):12283-91. doi: 10.3748/wjg.v20.i34.12283

18. Clave P, Acalovschi M, Triantafillidis JK, et al. Randomised clinical trial: otilonium bromide improves frequency of abdominal pain, severity of distention and time to relapse in patients with irritable bowel syndrome. *Aliment Pharmacol Ther* 2011;34(4):432-42. doi: 10.1111/j.1365-2036.2011.04730.x

For peer review only - http://bmjopen.bmj.com/site/about/guidelines.xhtml

19. Kwon JG, Park KS, Park JH, et al. [Guidelines for the treatment of irritable bowel syndrome]. *Korean J Gastroenterol* 2011;57(2):82-99.

20. Shin B, Lee B. Frequency Analysis of Clinical Prescriptions in the Korean Medicine Hospital, Pusan National University based on Herb Weight Ratio(2) - Focusing on Prescriptions of Herbal Formula Study Textbook[Korean]. *Herbal Formula Science* 2015;23(1):77-89. doi: dx.doi.org/10.14374/HFS.2015.23.1.077

21. Li C, Zuo M, Wang L, et al. Clinical observation of diarrhea - predominant irritable bowel syndrome treated by Shenlingbaizhu-san and Paroxetine [Chinese]. *Chinese Journal of Traditional Medical Science and Technology* 2010;17(2):154-5.

22. Leung WK, Wu JC, Liang SM, et al. Treatment of diarrhea-predominant irritable bowel syndrome with traditional Chinese herbal medicine: a randomized placebo-controlled trial. *Am J Gastroenterol* 2006;101(7):1574-80. doi: 10.1111/j.1572-0241.2006.00576.x

23. Park JK, Choi SY, Koh KW, et al. Combined utilization with herbal products and prescribed drugs: A result from health examinee-based national survey. *Korean J Health Policy Adm* 2006;16(1):1-16.

24. Ko SJ, Han G, Kim SK, et al. Effect of korean herbal medicine combined with a probiotic mixture on diarrhea-dominant irritable bowel syndrome: a double-blind, randomized, placebo-controlled trial. *Evid Based Complement Alternat Med* 2013;2013:824605. doi: 10.1155/2013/824605

25. Whitehead AL, Julious SA, Cooper CL, et al. Estimating the sample size for a pilot randomised trial to minimise the overall trial sample size for the external pilot and main trial for a continuous outcome variable. *Stat Methods Med Res* 2016;25(3):1057-73. doi: 10.1177/0962280215588241

26. Cha BK, Jung MS, Choi CH, et al. The effect of a multispecies probiotic mixture on the

For peer review only - http://bmjopen.bmj.com/site/about/guidelines.xhtml

symptoms and fecal microbiota in diarrhea-dominant irritable bowel syndrome: a randomized, double-blind, placebo-controlled trial. *J Clin Gastroenterol* 2012;46(3):220-7. doi: 10.1097/MCG.0b013e31823712b1

27. Kim HJ, Vazquez Roque MI, Camilleri M, et al. A randomized controlled trial of a probiotic combination VSL# 3 and placebo in irritable bowel syndrome with bloating. *Neurogastroenterol Motil* 2005;17(5):687-96. doi: 10.1111/j.1365-2982.2005.00695.x

28. Drossman DA, Patrick DL, Whitehead WE, et al. Further validation of the IBS-QOL: a disease-specific quality-of-life questionnaire. *Am J Gastroenterol* 2000;95(4):999-1007. doi: 10.1111/j.1572-0241.2000.01941.x

29. Lewis SJ, Heaton KW. Stool form scale as a useful guide to intestinal transit time. *Scand J Gastroenterol* 1997;32(9):920-4. doi: 10.3109/00365529709011203

30. Sugaya N, Izawa S, Saito K, et al. Effect of prolonged stress on the adrenal hormones of individuals with irritable bowel syndrome. *Biopsychosoc Med* 2015;9(1):4. doi: 10.1186/s13030-015-0031-7

31. Fadgyas-Stanculete M, Buga AM, Popa-Wagner A, et al. The relationship between irritable bowel syndrome and psychiatric disorders: from molecular changes to clinical manifestations. *J Mol Psychiatry* 2014;2(1):4. doi: 10.1186/2049-9256-2-4

32. Zhong Y, Wu Y. Forty cases of diarrhea - predominant irritable bowel syndrome treated by Shenlingbaizhu-san granule and Montmorillonite powder [Chinese]. *Jiangxi Journal of Traditional Chinese Medicine* 2013;44(7):45-6.

33. Xiong B. Thirty cases of irritable bowel syndrome treated by Shenlingbaizhu-san granule and Probiotics [Chinese]. *Jiangxi Journal of Traditional Chinese Medicine* 2011;42(5):22-3.

34. Giachetti A. Pharmacological studies on otilonium bromide. *Ital J Gastroenterol* 1991;23(8 Suppl 1):56-9.

For peer review only - http://bmjopen.bmj.com/site/about/guidelines.xhtml

For peer review only

35. Evangelista S. Otilonium bromide: a selective spasmolytic for the gastrointestinal tract. *J Int Med Res* 1999;27(5):207-22. doi: 10.1177/030006059902700501

36. Battaglia G, Morselli-Labate AM, Camarri E, et al. Otilonium bromide in irritable bowel syndrome: a double-blind, placebo-controlled, 15-week study. *Aliment Pharmacol Ther* 1998;12(10):1003-10.

37. Shi J, Tong Y, Shen JG, et al. Effectiveness and safety of herbal medicines in the treatment of irritable bowel syndrome: a systematic review. *World J Gastroenterol* 2008;14(3):454-62.

Figure legend

Figure 1. Flow chart of Trial. GIIM: Gastrointestinal internal medicine, FM: Family medicine, D-IBS: diarrhea-irritable bowel syndrome, P: placebo, OB: octylonium bromide, SRS: Samryungbaekchulsan

For peer review only

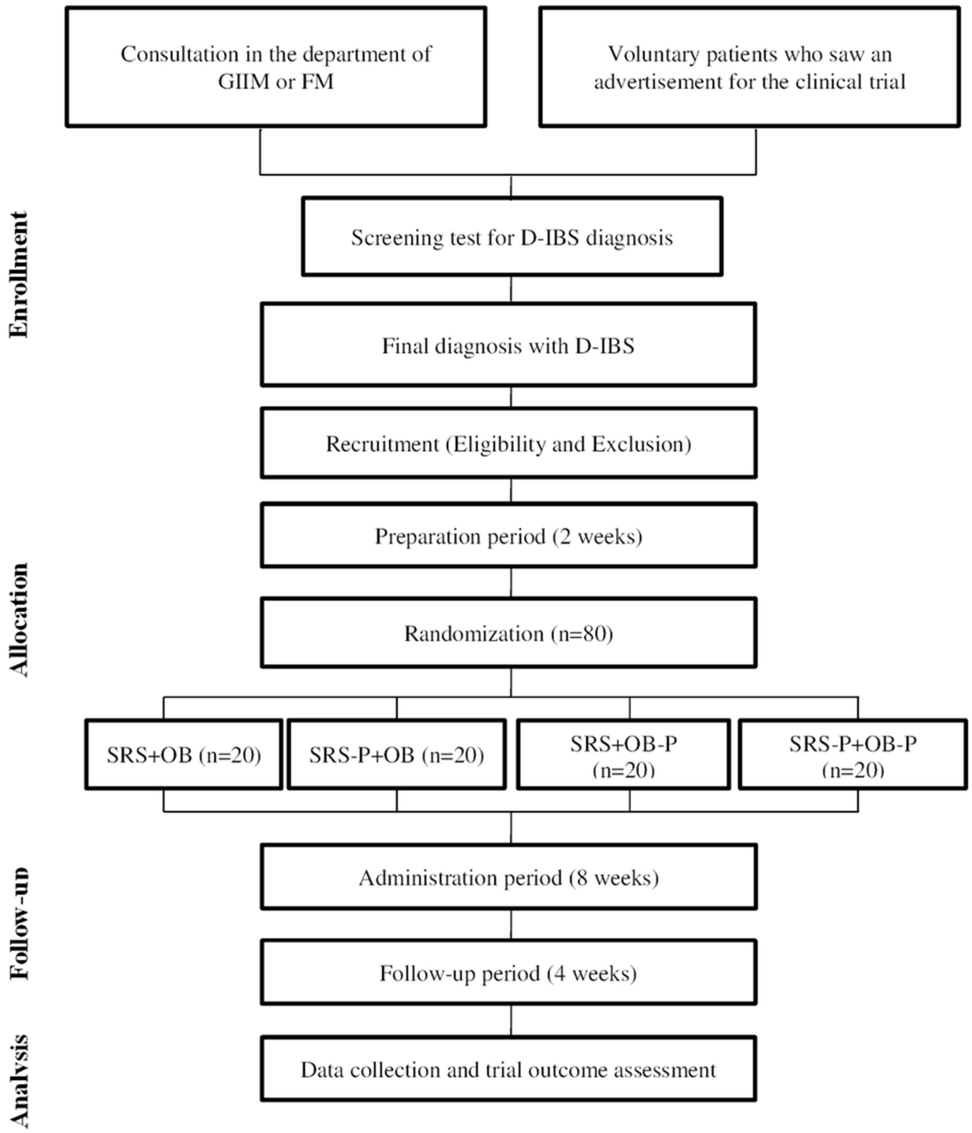

Figure 1. Flow chart of Trial. GIIM: Gastrointestinal internal medicine, FM: Family medicine, D-IBS: diarrhea-irritable bowel syndrome, P: placebo, OB: octylonium bromide, SRS: Samryungbaekchulsan

83x98mm (300 x 300 DPI)

SPIRIT

STANDARD PROTOCOL ITEMS: RECOMMENDATIONS FOR INTERVENTIONAL TRIALS

SPIRIT 2013 Checklist: Recommended items to address in a clinical trial protocol and related documents*

Section/item	Item No	Description	Addressed on page number
**Administrative information**			
Title	1	Descriptive title identifying the study design, population, interventions, and, if applicable, trial acronym	P1
Trial registration	2a	Trial identifier and registry name. If not yet registered, name of intended registry	P4
	2b	All items from the World Health Organization Trial Registration Data Set	Attachment
Protocol version	3	Date and version identifier	P4
Funding	4	Sources and types of financial, material, and other support	P1, P24
Roles and responsibilities	5a	Names, affiliations, and roles of protocol contributors	P1, P23
	5b	Name and contact information for the trial sponsor	P1, P24
	5c	Role of study sponsor and funders, if any, in study design; collection, management, analysis, and interpretation of data; writing of the report; and the decision to submit the report for publication, including whether they will have ultimate authority over any of these activities	N/A
	5d	Composition, roles, and responsibilities of the coordinating centre, steering committee, endpoint adjudication committee, data management team, and other individuals or groups overseeing the trial, if applicable (see Item 21a for data monitoring committee)	P8-9, P11-13, P17-19

For peer review only - http://bmjopen.bmj.com/site/about/guidelines.xhtml

**Introduction**			
Background and rationale	6a	Description of research question and justification for undertaking the trial, including summary of relevant studies (published and unpublished) examining benefits and harms for each intervention	P5-6
	6b	Explanation for choice of comparators	P6
Objectives	7	Specific objectives or hypotheses	P7-8
Trial design	8	Description of trial design including type of trial (eg, parallel group, crossover, factorial, single group), allocation ratio, and framework (eg, superiority, equivalence, noninferiority, exploratory)	P8-9
**Methods: Participants, interventions, and outcomes**			
Study setting	9	Description of study settings (eg, community clinic, academic hospital) and list of countries where data will be collected. Reference to where list of study sites can be obtained	P8
Eligibility criteria	10	Inclusion and exclusion criteria for participants. If applicable, eligibility criteria for study centres and individuals who will perform the interventions (eg, surgeons, psychotherapists)	P9-12
Interventions	11a	Interventions for each group with sufficient detail to allow replication, including how and when they will be administered	P12-14
	11b	Criteria for discontinuing or modifying allocated interventions for a given trial participant (eg, drug dose change in response to harms, participant request, or improving/worsening disease)	P14-17
	11c	Strategies to improve adherence to intervention protocols, and any procedures for monitoring adherence (eg, drug tablet return, laboratory tests)	P14-17,19
	11d	Relevant concomitant care and interventions that are permitted or prohibited during the trial	P14
Outcomes	12	Primary, secondary, and other outcomes, including the specific measurement variable (eg, systolic blood pressure), analysis metric (eg, change from baseline, final value, time to event), method of aggregation (eg, median, proportion), and time point for each outcome. Explanation of the clinical relevance of chosen efficacy and harm outcomes is strongly recommended	P14-16
Participant timeline	13	Time schedule of enrolment, interventions (including any run-ins and washouts), assessments, and visits for participants. A schematic diagram is highly recommended (see Figure)	P8-9, Figure1

For peer review only - http://bmjopen.bmj.com/site/about/guidelines.xhtml

Sample size	14	Estimated number of participants needed to achieve study objectives and how it was determined, including clinical and statistical assumptions supporting any sample size calculations	__P9__________
Recruitment	15	Strategies for achieving adequate participant enrolment to reach target sample size	__P11-12_______

Methods: Assignment of interventions (for controlled trials)

Allocation:			
Sequence generation	16a	Method of generating the allocation sequence (eg, computer-generated random numbers), and list of any factors for stratification. To reduce predictability of a random sequence, details of any planned restriction (eg, blocking) should be provided in a separate document that is unavailable to those who enrol participants or assign interventions	__P11-12_______
Allocation concealment mechanism	16b	Mechanism of implementing the allocation sequence (eg, central telephone; sequentially numbered, opaque, sealed envelopes), describing any steps to conceal the sequence until interventions are assigned	__P12__________
Implementation	16c	Who will generate the allocation sequence, who will enrol participants, and who will assign participants to interventions	__P11-12_______
Blinding (masking)	17a	Who will be blinded after assignment to interventions (eg, trial participants, care providers, outcome assessors, data analysts), and how	__P11-12_______
	17b	If blinded, circumstances under which unblinding is permissible, and procedure for revealing a participant's allocated intervention during the trial	__P11-12_______

Methods: Data collection, management, and analysis

Data collection methods	18a	Plans for assessment and collection of outcome, baseline, and other trial data, including any related processes to promote data quality (eg, duplicate measurements, training of assessors) and a description of study instruments (eg, questionnaires, laboratory tests) along with their reliability and validity, if known. Reference to where data collection forms can be found, if not in the protocol	__P17-19_______
	18b	Plans to promote participant retention and complete follow-up, including list of any outcome data to be collected for participants who discontinue or deviate from intervention protocols	__P8-9_________

For peer review only - http://bmjopen.bmj.com/site/about/guidelines.xhtml

Data management	19	Plans for data entry, coding, security, and storage, including any related processes to promote data quality (eg, double data entry; range checks for data values). Reference to where details of data management procedures can be found, if not in the protocol	P17,19
Statistical methods	20a	Statistical methods for analysing primary and secondary outcomes. Reference to where other details of the statistical analysis plan can be found, if not in the protocol	P17-19
	20b	Methods for any additional analyses (eg, subgroup and adjusted analyses)	P17-19
	20c	Definition of analysis population relating to protocol non-adherence (eg, as randomised analysis), and any statistical methods to handle missing data (eg, multiple imputation)	P17-19
**Methods: Monitoring**			
Data monitoring	21a	Composition of data monitoring committee (DMC); summary of its role and reporting structure; statement of whether it is independent from the sponsor and competing interests; and reference to where further details about its charter can be found, if not in the protocol. Alternatively, an explanation of why a DMC is not needed	P19
	21b	Description of any interim analyses and stopping guidelines, including who will have access to these interim results and make the final decision to terminate the trial	P17,19
Harms	22	Plans for collecting, assessing, reporting, and managing solicited and spontaneously reported adverse events and other unintended effects of trial interventions or trial conduct	P15-17
Auditing	23	Frequency and procedures for auditing trial conduct, if any, and whether the process will be independent from investigators and the sponsor	P19
**Ethics and dissemination**			
Research ethics approval	24	Plans for seeking research ethics committee/institutional review board (REC/IRB) approval	P3, P22
Protocol amendments	25	Plans for communicating important protocol modifications (eg, changes to eligibility criteria, outcomes, analyses) to relevant parties (eg, investigators, REC/IRBs, trial participants, trial registries, journals, regulators)	P22

For peer review only - http://bmjopen.bmj.com/site/about/guidelines.xhtml

Consent or assent	26a	Who will obtain informed consent or assent from potential trial participants or authorised surrogates, and how (see Item 32)	__P22-23_____
	26b	Additional consent provisions for collection and use of participant data and biological specimens in ancillary studies, if applicable	__N/A_________
Confidentiality	27	How personal information about potential and enrolled participants will be collected, shared, and maintained in order to protect confidentiality before, during, and after the trial	__P23_________
Declaration of interests	28	Financial and other competing interests for principal investigators for the overall trial and each study site	__P24_________
Access to data	29	Statement of who will have access to the final trial dataset, and disclosure of contractual agreements that limit such access for investigators	__P17_________
Ancillary and post-trial care	30	Provisions, if any, for ancillary and post-trial care, and for compensation to those who suffer harm from trial participation	__P23_________
Dissemination policy	31a	Plans for investigators and sponsor to communicate trial results to participants, healthcare professionals, the public, and other relevant groups (eg, via publication, reporting in results databases, or other data sharing arrangements), including any publication restrictions	__P3, 23_______
	31b	Authorship eligibility guidelines and any intended use of professional writers	__N/A_________
	31c	Plans, if any, for granting public access to the full protocol, participant-level dataset, and statistical code	__N/A_________
**Appendices**			
Informed consent materials	32	Model consent form and other related documentation given to participants and authorised surrogates	__Attachment__
Biological specimens	33	Plans for collection, laboratory evaluation, and storage of biological specimens for genetic or molecular analysis in the current trial and for future use in ancillary studies, if applicable	__P15-16_______

*It is strongly recommended that this checklist be read in conjunction with the SPIRIT 2013 Explanation & Elaboration for important clarification on the items. Amendments to the protocol should be tracked and dated. The SPIRIT checklist is copyrighted by the SPIRIT Group under the Creative Commons "Attribution-NonCommercial-NoDerivs 3.0 Unported" license.

For peer review only - http://bmjopen.bmj.com/site/about/guidelines.xhtml

BMJ Open

Effect of herbal extract granules combined with otilonium bromide on irritable bowel syndrome with diarrhea: a pilot study protocol for a randomized controlled trial

Journal:	*BMJ Open*
Manuscript ID	bmjopen-2017-018362.R2
Article Type:	Protocol
Date Submitted by the Author:	20-Oct-2017
Complete List of Authors:	Kim, Joong Il; Catholic Kwandong University International Saint Mary's Hospital, Institute for Integrative Medicine; Kim, Pumsoo; Catholic Kwandong University International Saint Mary's Hospital, Institute for Integrative Medicine Lee, Jin-Hyun; Wonkwang University College of Korean Medicine, Department of Korean Rehabilitation Medicine Kim, Yoo-Jin; Catholic Kwandong University International Saint Mary's Hospital, Institute for Integrative Medicine Yang, Na-rae; Catholic Kwandong University International Saint Mary's Hospital, Institute for Integrative Medicine Baeg, Myong Ki; Catholic Kwandong University International Saint Mary's Hospital, Institute for Integrative Medicine Choi, Ja Sung; Catholic Kwandong University International Saint Mary's Hospital, Institute for Integrative Medicine Kim, Hye-Jung; Catholic Kwandong University International Saint Mary's Hospital, Institute for Integrative Medicine Kim, Ja-Young; Catholic Kwandong University International Saint Mary's Hospital, Institute for Integrative Medicine Sunwoo, Yun-Young; Catholic Kwandong University International Saint Mary's Hospital, Institute for Integrative Medicine Lee, Jung-Han; Won-Kwang University, Department of Rehabilitation Medicine, College of Korean Medicine Ha, Hyekyung; Korea Institute of Oriental Medicine, K-herb Research Center Park, Tae-Yong; Catholic Kwandong University International Saint Mary's Hospital, Institute for Integrative Medicine
**Primary Subject Heading**:	Complementary medicine
Secondary Subject Heading:	Gastroenterology and hepatology
Keywords:	Samryungbaekchulsan, otilonium bromide, irritable bowel syndrome, randomized controlled study, clinical trial protocol

SCHOLARONE™
Manuscripts

For peer review only

Effect of herbal extract granules combined with otilonium bromide on irritable bowel syndrome with diarrhea: a pilot study protocol for a randomized controlled trial

Joong Il Kim[1*], Pumsoo Kim[1*], Jin-Hyun Lee[2], Yoo-Jin Kim[1], Na-rae Yang[1], Myong Ki Baeg[1], Ja Sung Choi[1], Hye-Jung Kim[1], Ja-Young Kim[1], Yun-Young Sunwoo[1], Jung-Han Lee[2], Hyekyung Ha[3], Tae-Yong Park[1]

[1] *Institute for Integrative Medicine, Catholic Kwandong University International St. Mary's Hospital, Incheon, 22711, South Korea*

[2] *Department of Rehabilitation Medicine, College of Korean Medicine, Wonkwang University, Iksan, 54383, South Korea*

[3] *K-herb Research Center, Korea Institute of Oriental Medicine, Daejeon, 34054, South Korea*

Corresponding author

Tae-Yong Park

Address: Institute for Integrative Medicine, Catholic Kwandong University International, St. Mary's Hospital, Simgok-ro 100gil 25, Seo-gu, Incheon, 22711, Republic of Korea

E-mail: parktae9822@gmail.com

Telephone: +82-32-290-3319

*** These authors contributed equally to the study**

Funding: This work was supported by a "Construction of Scientific Evidences for Herbal Medicine Formulas (K15250)" grant from the Korea Institute of Oriental Medicine (KIOM). (Telephone: +82-42-868-9513)

Word Count: 5898

For peer review only

For peer review only - http://bmjopen.bmj.com/site/about/guidelines.xhtml

Keywords: Samryungbaekchulsan, otilonium bromide, irritable bowel syndrome, randomized controlled study, clinical trial protocol

For peer review only

For peer review only - http://bmjopen.bmj.com/site/about/guidelines.xhtml

ABSTRACT

Introduction

Irritable bowel syndrome (IBS), known as a functional and organic gastrointestinal (GI) disorder, is a collection of symptoms that occur together and generally include pain or discomfort in the abdomen and changes in bowel movement patterns. Due to the limitations of conventional treatments, alternative IBS treatments are used by many patients worldwide. Samryungbaekchulsan (SRS), a herbal formula, has long been used for alleviating diarrhea-predominant IBS (D-IBS) in traditional Korean medicine. Otilonium bromide (OB) is an antimuscarinic compound used to relieve spasmodic pain in the gut, especially in IBS. Although herbal formulas and western drugs are commonly co-administered for various diseases in Korea, few clinical studies have been conducted regarding the synergic effects of these treatments for any disease, including D-IBS.

Methods and Analysis

This trial is a randomized, double-blinded, placebo-controlled, double-dummy, four-arm, parallel study. After a 2-week preparation period, 80 patients with D-IBS will be randomly assigned to one of four treatment groups consisting of SRS (water extract granules, 5 g/pack, 3 times a day) with OB (tablet form, 1 capsule 3 times a day) or their placebos, with treatment lasting for 8 weeks. Post-treatment follow-up will be conducted 4 weeks after the end of treatment. The primary outcome is the finding obtained using the Subject's Global Assessment of Relief method. The secondary outcomes are the severity of symptoms related to D-IBS, determined using a 10-point scale, and the change in symptoms.

Ethics and dissemination

This trial has full ethical approval of the ethics committee of Catholic Kwandong University International St. Mary's Hospital (IS15MISV0033) and the Korean Ministry of Food and Drug Safety (30769). The results of the study will be disseminated through a peer-reviewed journal and/or conference presentations.

For peer review only - http://bmjopen.bmj.com/site/about/guidelines.xhtml

Trial protocol version: IS15MISV0033 Version 4.0 (July 25, 2016)

Trial registration number: KCT0001621 (Approval date: Aug 10, 2015)

STRENGTHS AND LIMITATIONS OF THIS STUDY

■This randomized controlled trial is the first Korean clinical trial approved by the Korean Ministry of Food and Drug Safety (KMFDS) to investigate the efficacy and safety of Samryungbaekchulsan (SRS; herbal formula) and otilonium bromide (OB; western drug) in diarrhea-predominant irritable bowel syndrome (D-IBS).

■To evaluate the therapeutic effect and safety of the co-administration of SRS and OB, this study is proposed as a double-blind, double-dummy, four-arm, parallel-group randomized controlled trial.

■The primary outcome is the improvement in the patient's symptoms according to the Subject's Global Assessment of Relief and the secondary outcome is the severity of D-IBS symptoms.

■This study is conducted with a small sample size (four arms, 20 participants per group) because it is a pilot study and further clinical trials with powerful sample sizes will be required to more adequately determine the effectiveness of SRS and/or OB for D-IBS.

For peer review only - http://bmjopen.bmj.com/site/about/guidelines.xhtml

INTRODUCTION

Irritable bowel syndrome (IBS), a chronic gastrointestinal (GI) disease and functional bowel disorder, is characterized by abnormal bowel habits and abdominal pain without structural, morphological, and histological abnormalities. It affects approximately 20% of the population worldwide, according to population-based studies[1-6]. In addition, recent evidence suggests that IBS is not only a functional disease, but also an organic disease with a complex of symptoms, including infection, immune activation, serotonin dysregulation, bacterial overgrowth, central dysregulation, brain-gut interaction, and genetics[4-8]. Based on their predominant bowel habits, patients with IBS can be divided into diarrhea-predominant IBS (D-IBS), constipation-predominant IBS, or mixed IBS[2 6 9]. Recently, smooth-muscle relaxants, bulking agents, and anti-diarrheal agents have been used in the treatment of IBS[10]. However, these methods are often not effective and many IBS patients also use complementary treatments, such as herbal medicine, acupuncture, psychological treatment, and lifestyle correction[2 11 12].

Samryungbaekchulsan (SRS; Shenlingbaizhu-san in Traditional Chinese Medicine; Jinryobyakujutsu-san in Kampo Medicine), which was introduced in the famous classical compendium of herbal formulas “Formulary of Peaceful Benevolent Dispensary,” consists of 10 herbs (Atractylodes rhizoma Alba, Poria sclerotium, Dioscoreae rhizoma, Glycyrrhizae radix et rhizoma, Coicis semen, Nelumbinis semen, Platycodonis radix, Dolichoris semen, Amomi fructus, and Ginseng radix). In traditional Korean and Chinese medicine, this herbal formula has been used to treat GI disease, and various studies have been published regarding the treatment of IBS with SRS[13-18]. SRS has been shown to have anti-acetylcholine and anti-barium chloride effects and to suppress gastric secretion and small intestine motility[13]. However, no clinical trials have evaluated the efficacy of SRS for IBS when co-administered with a conventional drug.

For peer review only - http://bmjopen.bmj.com/site/about/guidelines.xhtml

Otilonium bromide (OB) is widely used for the prevention and reduction of symptoms in patients with IBS[7 8]. OB has been shown to inhibit L- and T-type calcium channels, muscarinic receptors, and tachykininergic responses in human cultured smooth muscle cells and rat colon strips[19-21]. According to a recent randomized controlled trial (RCT), OB (40 and 80 mg) is more effective for treating IBS than placebo, not only for the reduction of bloating and abdominal pain, but also for protection from relapse owing to its long-lasting effect[22 23].

According to the guidelines of the Korean Society of Gastroenterology, antispasmodic agents are recommended for the treatment of D-IBS (Grade 1B),[24] and SRS is the preferred drug for diarrhea treatment in Korean medicine[18]. Recently, several studies have been conducted to evaluate the therapeutic effect of the co-administration of herbal formulas and conventional drugs for IBS in China[25 26]. However, there has been no such clinical study in Korea, even though both herbal medicine and conventional drugs are widely used for IBS treatment in the clinical field. Therefore, there is a need to evaluate the efficacy and safety of SRS, OB, and the co-administration of both drugs.

In this study, we expect that the combined administration of OB and SRS will result in synergistic effects that improve D-IBS symptoms, including abdominal pain, discomfort (the main effect of OB), and diarrhea-like stool patterns (the main effect of SRS).

For peer review only - http://bmjopen.bmj.com/site/about/guidelines.xhtml

METHODS AND ANALYSIS

Objectives

The purpose of this study is to evaluate the efficacy and safety of the co-administration of SRS and OB to patients with D-IBS.

Necessity of the trial

Recently, herbal medicines and western drugs have been co-administered for various medical therapies. This is particularly true in Korea, where approximately 76% of patients have used the combination of herbal medicines and western drugs[14]. However, only a few studies have evaluated the interaction between western and herbal medicines. Despite the need for combined treatment in patients with various diseases, there are no standard clinical guidelines or scientific evidence regarding co-administration because of a lack of research and data.

D-IBS is a disease that is difficult to cure and many patients require treatment with both western and herbal drugs to experience an improvement in their symptoms. Therefore, it is very important to conduct a clinical study regarding the efficacy and safety of the combined administration of SRS and OB in patients with D-IBS.

Basis of drug selection

According to guidelines for IBS treatment, the OB recommendation level is high (i.e., the level of evidence is moderate and many experts recommend using it) and it is the most reasonable treatment option[25]. SRS is the herbal formula used most often by clinical Korean and Chinese medical doctors for abdominal pain, abdominal discomfort, and chronic diarrhea, which are typical symptoms of D-IBS[15-17 24]. Based on these studies and clinical applications, OB and SRS were selected for this trial.

For peer review only - http://bmjopen.bmj.com/site/about/guidelines.xhtml

Hypothesis

We hypothesize the following: (1) that co-administration of SRS and OB will have a more positive effect on D-IBS symptoms than either SRS or OB alone; (2) that after 8 weeks of treatment with SRS, OB, or both drugs, the Subject's Global Assessment of Relief and severity of D-IBS symptoms will be more improved than after treatment with placebo; and (3) that co-administration of SRS and OB will be safe for patients with D-IBS.

Design

This study is a placebo-controlled, double-blind trial. Eighty patients will be enrolled and randomly assigned to four separate arms of the trial. The trial will be conducted at Catholic Kwandong University International St. Mary's Hospital, Incheon, South Korea.

The study subjects will be required to participate in a 2-week preparation period (weeks -2 to 0) and an 8-week drug administration period (weeks 0 to 8). The patients will visit four times, on weeks 0, 4, 8, and 12. The primary outcome measurement will be evaluated at weeks 2, 4, 6, 8, and 12, and the secondary outcome measurement will be evaluated at weeks 0, 2, 4, 6, 8, and 12. Participants will be assessed for efficacy and safety of the treatment through visits (0, 4, 8, 12 weeks) and telephone questionnaires (2, 6 weeks). In addition, lab tests will be conducted at 0, 4, and 8 weeks. According to the intervention protocols, the clinical research coordinator (CRC) will encourage patients and participants to complete follow-up at every visit.

During the drug administration period, SRS or its placebo will be given as 1 pack of water extract granules 3 times a day, 30 min before each meal. OB or its placebo will be given as 1 tablet 3 times a day, 30 min before each meal.

Participants will be divided into 4 groups: (1) SRS and OB group; (2) SRS and placebo-

For peer review only - http://bmjopen.bmj.com/site/about/guidelines.xhtml

OB group; (3) placebo-SRS and OB group; and (4) placebo-SRS and placebo-OB group. The entire flow chart of the study is shown in Figure 1.

The protocol of this trial has been approved by the institutional review board (IRB) and ethics committee of Catholic Kwandong University International St. Mary's Hospital. The IRB permission number is IS15MISV0033 and CRIS (Clinical Research Information Service, www.cris.nih.go.kr) protocol identification number is KCT0001621. In addition, this study received the first approval given by the Korean Ministry of Food and Drug Safety (KMFDS) for a study of the co-administration of a conventional drug and a Korean traditional herbal medicine (approval number: 30769).

Potential participants will be provided with sufficient information about the study and only those who agree to the study protocol and sign the consent form will be enrolled. The study will be implemented according to Good Clinical Practice and the revised version of the Declaration of Helsinki.

Sample size calculation

This trial is a pilot study for a new therapeutic regimen of SRS and OB combination treatment. In a similar study conducted previously by using co-administration of a probiotics mixture and an herbal medicine (placebo-controlled, double-blind, and randomly 4-arm), each arm consisted of at least 15 patients and the total sample size was more than 60 participants[27]. In other similar pilot studies, clinical trials have been conducted with a total of 60–64 participants, consisting of 12–30 patients per group[28-30]. Based on these previous studies, the minimal clinical significance of the trial can be obtained when only 16 participants complete the trial. Because the drop-out rate is expected to be 20%, each trial group will be made up of at least 20 participants. Therefore, the total sample size must be more than 80 participants.

For peer review only - http://bmjopen.bmj.com/site/about/guidelines.xhtml

Inclusion criteria

The following patients would be included: (1) patients aged 18–75 years; (2) patients satisfying the Rome III criteria of IBS (recurrent abdominal pain or discomfort that began at least 6 months before the clinical trial, with a rating of more than 3 on a 10-point scale, frequency of at least 3 days/month in the last 3 months, and associated with two or more of the following: improvement associated with defecation, onset associated with a change in frequency of stools, and onset associated with a change in form of stool)[1 9]; (3) patients satisfying the Rome III criteria of IBS, whereby D-IBS type is defined as loose/mushy or watery stools—Bristol Stool Form (BSF) Scale 6-7 ≥ 2 times/week[1 30]; (4) patients with a negative urine pregnancy test (in women of a childbearing age, within 7 days before the clinical trial) and who consent to using contraception during the administration period; (5) patients with the ability to read the symptom questionnaire and understand it; and (6) patients who agree with the clinical plan and voluntarily sign the IRB-approved documents.

Exclusion criteria

The exclusion criteria are as follows: (1) chronic liver disease (cirrhosis, chronic hepatitis B or C); (2) chronic renal failure or renal impairment (serum creatinine ≥ 2 times the upper limit of normal); (3) liver dysfunction (AST/ALT ≥ 3 times the upper limit of normal); (4) diabetes (HbA1c > 8% or not controlled by diet or medication), hypertension (≥ 160/100 mmHg), thyroid dysfunction (exceptions in the following cases: if the disease is controlled by drug administration with a stable dose for 12 weeks before the screening, and the drug dose remains constant during the trial period), clinically significant hematologic, cardiac, pulmonary, neurological disorders, or other severe systemic disorders; (5) abnormal findings on colonoscopy or colonography within the last 5 years; (6) history of surgery that affects

For peer review only - http://bmjopen.bmj.com/site/about/guidelines.xhtml

gastrointestinal motility (i.e., gastrectomy, colonic resection, hysterectomy, except for appendectomy); (7) GI disease characterized by the following symptoms within 6 months before the clinical trial: inflammation of or ulcer in the esophagus, stomach, or duodenum; gastroesophageal reflux disease (at the discretion of the examiner, subjects may enroll if the disease is chronic and not acute); gastrointestinal bleeding; gastrointestinal stenosis or closure; infectious diarrhea; inflammatory bowel disease (i.e., Crohn's disease, diverticulitis, ulcerative colitis, infectious enteritis, ischemic colitis); pancreatic insufficiency; biliary abscess; (8) mental illness or an addiction to drugs or alcohol; (9) severe systemic organ diseases such as cancer, autoimmune disease, stroke (although patients in remission for more than 5 years from a cancer unrelated to the GI tract can participate in the trial); (10) pregnancy, breastfeeding, and unwillingness to use contraception during the trial; (11) a history of taking the following drugs within 2 weeks before the trial: antibiotics, analgesics, antidepressants, anti-anxiety agents, anti-inflammatory agents, anti-ulcer agents, anti-gastric secretion inhibitors, laxatives, antispasmodics, antacid agents, gastrointestinal stimulants, prostaglandin agents, and corticosteroids; (12) use of medications that do not match the intent of this trial or have a clinical interaction with SRS or OB; (13) lactose intolerance (not controlled by food); (14) glaucoma; (15) participation in other clinical studies within 30 days prior to clinical screening and have received other clinical trial medications (including placebo); (16) hypersensitivity to clinical trial medicines; and (17) other reasons considered inappropriate for participation in clinical trials.

Recruitment, randomization, blinding, and unblinding

Advertisements for the study will be posted at the Catholic Kwandong University International St. Mary's Hospital using banner ads and posters. We also plan to post the advertisements on the hospital homepage, in the monthly hospital magazine, at the internet

For peer review only - http://bmjopen.bmj.com/site/about/guidelines.xhtml

For peer review only

café, and on the subway at least five times. All these advertisements will be done with the approval of the IRB.

After recruitment, patients will be randomized to one of the four trial arms. Randomization will be performed using a list of block randomizations made by statisticians, independent of this clinical trial. A researcher who is not involved in process of drug prescription and evaluation will execute the randomization process.

During the treatment period, the patients and all researchers (investigators and clinical research coordinators) will be blinded, except the researchers who conducted the randomization procedure. In addition, the researchers will not know the kind of medication that is being administered to the patient or any other information that could lead to bias.

Allocation concealment will be performed during the process of medication administration. The management pharmacist, who will not be involved in analyzing the results of the trial, will use the randomization table to label the medication with the assignment number and provide the medication. The patients will take the drugs contained in the opaque envelope, which will be labeled with the assignment number.

Unblinding will be performed only in the following situations: (1) if the trial is terminated and there is a need for statistical analysis; (2) if a serious medical emergency occurs and information about the medication is needed; and (3) if the chief investigator determines that unblinding is necessary.

The entire process of the study will be directed by the authorized clinical research organization (CRO), Dream CIS Corporation, Seoul, Korea.

Intervention

The herbal formula SRS is commonly used by Korean traditional medical doctors for treatment of diarrhea and related diseases[13-18]. SRS used in this study (Samryungbaekchulsan

For peer review only - http://bmjopen.bmj.com/site/about/guidelines.xhtml

granule®, Hankook shin yak, Co., Ltd., Nonsan, Korea) is extracted with water and mixed with starch and lactose in accordance with Korean Good Manufacturing Practice (K-GMP). Production of SRS is regulated and allowed by the KMFDS. SRS contains 10 herbs, listed in Table 1. Placebo-SRS is made from cornstarch and has the same taste, shape, color, and similar scent as SRS. SRS and placebo-SRS will be sealed in identical aluminum bags with the same labeling. These herbal drug packages will be distributed by an independent pharmacist in an isolated room. According to the recommended dosing methods of KMFDS, after SRS or placebo-SRS is dissolved in boiled water, the subjects will take them 30 min before each meal (5 g/pack, 3 times a day).

OB (Menoctyl Tab®, Dong Hwa Pharm Co., LTD., Seoul, Korea) is a tablet containing otilonium bromide 40 mg. Placebo-OB is a tablet made of Ludipress with the same taste, shape, and color as OB. Patients will be instructed to swallow one tablet of OB or placebo-OB 30 min before each meal over the administration period. The drugs used in the trial (SRS, OB, placebo-SRS, placebo-OB) have been approved by the KMFDS. Patients will be asked at the end of the study whether the drugs that they have been taking are either real or placebo, to measure the success of blinding. Compliance will be confirmed by counting returned SRS packets or OB tablets. Every patient will be instructed to write down any adverse events during the administration period and these records will be evaluated at the follow-up. At the patient visits (at 0, 4, and 8 weeks), we will perform a check of vital signs, physical examination, electrocardiogram, and laboratory tests to evaluate safety for the patients. All side effects will be reported to the investigator.

For peer review only - http://bmjopen.bmj.com/site/about/guidelines.xhtml

Table. 1 Ingredients in the herbal formula Samryungbaekchulsan (SRS)

Scientific name	Part used	Grams/day
Atractylodis Rhizoma Alba	Root	3.0
Poria Sclerotium	Dried core	4.0
Rhizoma Dioscoreae	Root	3.0
Glycyrrhizae Radix et Rhizoma	Root	1.5
Semen Coicis	Seed	8.0
Semen Nelumbinis	Fruit	4.0
Platycodonis Radix	Root	2.5
Dolichoris Semen	Seed	4.0
Amomi Fructus	Fruit	2.0
Ginseng Radix	Root	3.0

Rescue therapy and concomitant medications

Participants are prohibited from taking any medication that could affect D-IBS during the entire course of the trial. However, if serious side effects occur or symptoms worsen, investigators will provide quick and appropriate treatment (including drug administration), based on their medical judgement. In this case, the direct investigators will record the treatment or drug administration and report to the IRB within 24 h to determine whether the trial should be continued or discontinued.

Outcome Measurements

Primary outcome

The primary outcome will be measured via a questionnaire that confirms subjective

For peer review only - http://bmjopen.bmj.com/site/about/guidelines.xhtml

symptom improvement. Participants will respond a total of five times during and after the administration period (at 2 and 6 weeks via telephone, and at 4, 8, and 12 weeks during direct visits) to the following question: Subject's Global Assessment of Relief; "How much do you think the symptoms of D-IBS have improved compared to before the clinical trial?" According to the answers given by the patients to this survey, the improvement is given a score of 0, 1, 2, 3, or 4.

Secondary outcomes

At 2, 4, 6, 8, and 12 weeks from the start of the treatment period, patients will be assessed for severity of D-IBS symptoms (abdominal pain, abdominal discomfort, satisfaction of defecation, frequency of abdominal pain, quality of life) using a Likert scale ranging from 0 to 10[31]. Patients will also be instructed to record the number of defecations per day, BSF, and the degree of force used in bowel movements at 0, 2, 4, 6, 8, and 12 weeks[32].

Safety assessments

The investigator will record all adverse events that occur during the trial along with any concomitant medications in the case reports. When an adverse event occurs, the investigator will record the symptoms and signs of the adverse reaction, the duration (start and end date), severity, course, outcome, significance, causality by the trial drug, and any action taken in relation to the adverse event. In the case of concomitant medicines, composition, dosage, duration of administration, and reason for medication will be recorded in detail. Symptoms that existed before the start of the clinical trial will not be recorded as adverse events.

Laboratory tests

The purpose of laboratory tests in this study is to evaluate the safety and pharmacokinetic

For peer review only - http://bmjopen.bmj.com/site/about/guidelines.xhtml

profiles of SRS and OB. Because of the small number of patients assigned to this study and the different baseline values for each individual patient, we will not perform a detailed statistical analysis. However, if significant abnormal results are found in the laboratory tests, the clinical laboratory results will be described and the connection to the drug will be considered.

Blood tests will be conducted at 0, 2, 4, and 8 weeks and urine tests will be performed at 0 and 4 weeks during the clinical trial. In the case of a participant who drops out or the early termination of the clinical trial, blood and urine tests will be performed on the last day of the trial.

Using the test results (comprehensive verification of vital signs, laboratory tests, electrocardiograms, and physical examinations) for each individual patient, the investigator will determine adverse reactions caused by the drugs and statistical tests will be conducted as needed on clinically significant parameters.

Test of human derivatives

IBS is known to be closely related to stress[12]. Therefore, we plan to quantify factors associated with stress or that have been reported to worsen IBS symptoms as follows: 1) cortisol; 2) corticotropin-releasing hormone (CRH); 3) serotonin; 4) group I cytokines (10 species); 5) group II cytokines (14 species); and 6) growth factors (3 species)[33 34]. Serum will be separated from blood specimens collected at 0, 4, 8 weeks, stored frozen, and transferred to the Korea Institute of Oriental Medicine (KIOM) for examination.

Early termination or dropout

The criteria for early termination or dropout are as follows: 1) administration of other drugs that are expected to affect the safety and efficacy of the clinical trial drugs; 2) request

For peer review only - http://bmjopen.bmj.com/site/about/guidelines.xhtml

of participants to discontinue the clinical trial, or withdrawal of the trial agreement; 3) occurrence of significant adverse drug reactions or events that preclude continuing the trial, according to the judgment of the clinical investigator; 4) new discovery of violations of significant clinical trial protocol during clinical trial; 5) less than 70% trial drug compliance; and 6) any other reason that the clinical trial should be discontinued at the discretion of the clinical trial manager or investigator.

Data collection, access, and management

The data of this trial will be managed according to the standard work instructions of Catholic Kwandong University International, St. Mary's Hospital. Other contents not specified in the trial protocol shall follow the standards of The International Conference on Harmonization of Technical Requirements for Registration of Pharmaceuticals for Human Use (ICH) Guideline for Good Clinical Practice (GCP) and Korean Good Clinical Practice (KGCP) guideline.

The source document is recorded immediately when the data are collected. After the source document input is completed, it will be recorded in the case record form (CRF). All the documents will be kept safe, so that the data can be verified by the relevant government agencies and the IRB. Only research colleagues and others who have been approved by the principle investigator will have access to all data obtained from this trial.

Statistical analysis

The statistical analysis will be conducted by blinded professional statisticians. To evaluate the efficacy of the study, both an intent-to-treat (ITT) analysis (primary analytical method) and a per-protocol (PP) analysis (secondary analytical method) will be performed. The ITT analysis will be conducted for all patients who were followed up at least once after

For peer review only - http://bmjopen.bmj.com/site/about/guidelines.xhtml

randomization and will be able to be evaluated for effectiveness. The PP analysis will be performed for patients who complete the 8-week treatment course and meet the inclusion and exclusion criteria.

To analyze the continuous variables in the demographic information and pre-treatment characteristics between groups, a one-way analysis of variance (ANOVA) and Kruskal-Wallis test will be conducted. An ANOVA and Kruskal-Wallis test will also be used to test for symptom improvement compared to baseline (0 week) and the variables for analysis that include the following: (1) subjective symptom improvement (primary outcomes); and (2) severity of symptoms related to D-IBS (abdominal pain, abdominal discomfort, satisfaction of defecation, frequency of abdominal pain, quality of life). All values will be shown as mean ± standard error of the mean (SEM). Comparisons of outcome assessments between the four groups will be performed using the paired t-test or Wilcoxon signed-rank test.

If the Subject's Global Assessment of Relief on the Likert scale is "0" or "1" for more than 2 weeks between weeks 8 and 12, then the symptoms will be considered to have improved sufficiently. Based on this, we will calculate the symptom relief rate by dividing the number of participants who exhibited sufficient symptom relief by the total number of participants in the trial and present descriptive statistics. The CATMOD procedure will be used to evaluate the relief rate improvement of symptoms compared with baseline. Comparisons between the four groups will be conducted using the chi-square test or Fisher's exact test. These statistical analyses will also be performed at 2, 4, 6, 8, and 10 weeks from baseline. The Cochran-Mantel-Haenszel (CMH) test will be used to confirm the difference between the intervention groups at each time-point.

A statistical analysis will also be conducted to evaluate safety. The incidence of adverse events and abnormality of experimental results will be analyzed statistically according to the groups and non-parametric methods will be applied as necessary using a paired t-test for

For peer review only - http://bmjopen.bmj.com/site/about/guidelines.xhtml

continuous data, McNemar's test for categorical data, Fisher's exact test for adverse events, and a generalized estimating equation for clinically significant changes between groups.

All statistical analysis will be conducted using SAS software, version 9.1.3 (SAS institute, Cary, NC). A P-value < 0.05 will be considered statistically significant.

Quality control and data monitoring

To ensure the results and quality of the clinical trial, assessments and monitoring will be performed by the CRO, DreamCIS Corporation (Seoul, Korea), which is independent from the sponsor and competing interests. During the trial, the CRO will regularly monitor whether the study is proceeding in accordance with the protocol through the use of all related documentation, including trial master files, case report forms, informed consent forms, and adverse event reports.

DISCUSSION

Herbal medicine is a recognized effective treatment method based on clinical experience accumulated over thousands of years and more recently gained attention as an alternative therapy for treating various diseases that are difficult to treat or have unknown causes, such as IBS[5 7 10 26 35]. SRS is frequently used for abdominal pain and diarrhea in Korean and Chinese traditional medicine. According to experimental research, SRS has an anti-cholinergic effect that improves intestinal digestion and absorption and inhibits intestinal movement, resulting in the prevention of diarrhea and abdominal pain[13]. In addition, clinical studies have shown that the use of SRS in patients with D-IBS is effective for improving diarrhea[36 37]. Some studies have reported that co-administration with SRS and trimebutine maleate or SRS and paroxetine is more effective than trimebutine maleate alone for the improvement in IBS symptoms[15 16 25]. Other studies have reported that SRS exerted more

For peer review only - http://bmjopen.bmj.com/site/about/guidelines.xhtml

beneficial effects on IBS patients compared with Smecta or probiotics[17 37].

However, although there is a growing interest in the use of herbal medicines such as SRS in IBS and related studies are continuously being published, there have been only a few well-designed RCTs[22 23 26]. To overcome the limitations of the previous studies, this trial was designed as a double-blind, double-dummy, four-arm, parallel-group RCT. The efficacy of SRS will be evaluated via the Subject's Global Assessment of Relief and the improvement in symptoms associated with bowel movements and abdominal pain. The results of these assessments are expected to provide distinct clinical evidence for the use of SRS in D-IBS treatment.

OB is a representative conventional medicine used for D-IBS treatment[8 21 23 38 39]. It is a spasmolytic agent that exerts its activity primarily in the distal GI tract through the inhibition of Ca++ flux and direct activation of contractile proteins in the smooth muscle[20 21]. OB has also been shown to reduce hyper-motility and modulate visceral sensation, the effect of which is to improve D-IBS symptoms[39 40]. RCTs using OB for the treatment of D-IBS have been conducted. In these studies, treatment with OB resulted in a greater improvement in symptoms related to D-IBS (defecation frequency, regular intestinal habits, reductions in frequency of diarrhea, abdominal pain and discomfort, severity of abdominal bloating) and better protection from symptom relapse compared to placebo[22 23 38].

IBS is a disease with a high rate of progression and frequent recurrence. For this reason, patients with IBS often require a variety of treatment methods[2 6 10]. Among these methods, the combination of herbal and conventional drugs is widely used and is more effective for improving IBS symptoms than conventional drugs alone[2 35]. However, there are several limitations to the co-administration of herbal and conventional drugs in IBS. Specifically, there is a high risk of bias in related studies and there are insufficient clinical guidelines regarding the interaction of herbal and conventional drugs in D-IBS, including SRS and OB.

For peer review only - http://bmjopen.bmj.com/site/about/guidelines.xhtml

Therefore, it is necessary to perform well-designed RCTs to evaluate the co-administration of herbal and conventional drugs.

Because only a few studies have evaluated the combination of herbal and conventional drugs, our proposed study will significantly contribute to overcoming the limitations of previous studies and provide basic medical evidence for the co-administration of SRS and OB for D-IBS treatment. Therefore, this RCT has several characteristic features. First, this trial will be the first to investigate the efficacy of SRS and OB combination therapy for D-IBS. The results of previous studies suggested that the combination of OB and SRS will improve two major D-IBS symptoms at the same time: abdominal pain and discomfort (major effect of OB) and diarrhea (main effect of SRS). This study will assess this hypothesis and provide a basis for the use of SRS and OB combination therapy for D-IBS. Second, the participants in this trial will be grouped into four arms, making it possible to compare the efficacy of combined treatment with the efficacy of each individual treatment. Third, this trial will evaluate the safety of the subjects using several assessments, including a periodic assessment of subjective symptoms, physical measurements, and laboratory testing. The results of the safety analysis may provide evidence for the safety of treatment with the combination of herbal and conventional drugs in D-IBS. Fourth, this trial was the first clinical study related to combination treatment with western and traditional herbal medicine in Korea to receive approval from the KMFDS. Through the systematic management of the trial under the auspices of the KMFDS, a high quality trial will be performed and the results of this study will provide information for use in further clinical applications.

Although our study is a pilot study with a small sample size, it has a creative and systematic research design and the results will be useful basic clinical evidence in the field of integrated herbal and conventional drugs for the management of D-IBS.

For peer review only - http://bmjopen.bmj.com/site/about/guidelines.xhtml

Trial status

The research plan and design began in April 2015.

The study received the IRB's final approval after two deliberations. The first submission date was July 01, 2015; the result was a request for revision and supplementation, which was received on July 13, 2015. We filed an application for re-review on August 06, 2015, supplemented with the relevant information, and received the IRB's final approval on August 10, 2015. We also requested that the KMFDS review the trial plan on September 14, 2015 and received approval for the clinical research on December 21, 2015.

The first participant was randomized on March 29, 2016. Recruitment for the study is ongoing. The primary completion date for this trial is anticipated to be December 31, 2017, and the study completion date is expected to be February 28, 2018.

Ethics

Research ethics approval

This trial was approved by the IRB and ethics committee of Catholic Kwandong University International St. Mary's Hospital (approval number IS15MISV0033) and the KMFDS (approval number 30769).

Protocol amendments

An investigator who wishes to change the protocol of the clinical trial plan should first discuss it with the trial director. Thereafter, the examiner should obtain prior approval from the IRB for changes to the protocol (except to prevent immediate injury to the patient, in which case it will be reported to the IRB later). However, when a dangerous situation occurs and immediate treatment is needed, the investigator may report the protocol change to the IRB at a later time.

For peer review only - http://bmjopen.bmj.com/site/about/guidelines.xhtml

Consent

Patients recruited through the announcement will receive a full explanation of the details of the trial in an easy-to-understand manner. The patients and investigators will have a mutual question and answer time before the patient is asked to sign the clinical trial consent. Through these processes, if the patient agrees to participate in the trial, a signed consent form will be obtained.

Confidentiality

All patient information will be anonymized with initials or symbols and all related investigators will keep the trial results confidential. The trial director will keep the signed consent forms and prepare a list that will be used to confirm the patient identities.

Post-trial care

If the patients experience unexpected accidents or injuries, appropriate compensation will be made by the Federal Insurance Company Korea (12th Floor Ferrum Tower 66 Suha-Dong Jung-gu Seoul, 100210, South Korea), according to the patient compensation rules of the trial. Additionally, in the case of an emergency, the patients will receive appropriate medical care at the Catholic Kwandong University International, St. Mary's Hospital.

Dissemination

The results of the trial will be disseminated through a peer-reviewed journal and/or conference presentations.

Contributors

For peer review only - http://bmjopen.bmj.com/site/about/guidelines.xhtml

For peer review only

Joong Il Kim, Pumsoo Kim, Jin-Hyun Lee, and Jung-Han Lee contributed to the protocol design and writing of the manuscript. Hyekyung Ha is responsible for monitoring the process of the trial. Na-rae Yang, Yoo-Jin Kim, Myong Ki Baeg, Ja Sung Choi, Hye-Jung Kim, Ja-Young Kim and Yun-Young Sunwoo provide advice on the process of the trial. Tae-Yong Park is responsible for writing the manuscript and also managing and supervising the clinical research. All authors approved the final manuscript.

Funding

This work was supported by a "Construction of Scientific Evidences for Herbal Medicine Formulas (K15250)" grant from the Korea Institute of Oriental Medicine (KIOM).

(Telephone: +82-42-868-9513)

Competing interests

The authors have no competing interests to declare.

Ethics approval

This trial has been approved by the IRB and ethics committee of Catholic Kwandong University International St. Mary's Hospital (approval number IS15MISV0033).

Provenance and peer review

Not commissioned; externally peer reviewed.

Open Access

This is an Open Access article distributed in accordance with the Creative Commons Attribution Non-Commercial (CC BY-NC 4.0) license, which permits others to distribute,

For peer review only - http://bmjopen.bmj.com/site/about/guidelines.xhtml

remix, adapt, build upon this work non-commercially, and license their derivative works on different terms, provided the original work is properly cited and the use is non-commercial. See: http://creativecommons.org/licenses/by-nc/4.0/

© Article author(s) (or their employer(s) unless otherwise stated in the text of the article) 2017. All rights reserved. No commercial use is permitted unless otherwise expressly granted.

REFERENCES

1. Drossman DA. Introduction. The Rome Foundation and Rome III. *Neurogastroenterol Motil* 2007;19(10):783-6. doi: 10.1111/j.1365-2982.2007.01001.x

2. Grundmann O, Yoon SL. Irritable bowel syndrome: epidemiology, diagnosis and treatment: an update for health-care practitioners. *J Gastroenterol Hepatol* 2010;25(4):691-9. doi: 10.1111/j.1440-1746.2009.06120.x

3. Maxion-Bergemann S, Thielecke F, Abel F, et al. Costs of irritable bowel syndrome in the UK and US. *Pharmacoeconomics* 2006;24(1):21-37.

4. Soares RL. Irritable bowel syndrome: a clinical review. *World J Gastroenterol* 2014;20(34):12144-60. doi: 10.3748/wjg.v20.i34.12144

5. Saha L. Irritable bowel syndrome: pathogenesis, diagnosis, treatment, and evidence-based medicine. *World J Gastroenterol* 2014;20(22):6759-73. doi: 10.3748/wjg.v20.i22.6759

6. Chey WD, Kurlander J, Eswaran S. Irritable bowel syndrome: a clinical review. *JAMA* 2015;313(9):949-58. doi: 10.1001/jama.2015.0954

7. Boeckxstaens G, Corazziari ES, Mearin F, et al. IBS and the role of otilonium bromide. *Int J Colorectal Dis* 2013;28(3):295-304. doi: 10.1007/s00384-012-1598-0

8. Boeckxstaens G, Clave P, Corazziari ES, et al. Irritable bowel syndrome: focus on

For peer review only - http://bmjopen.bmj.com/site/about/guidelines.xhtml

otilonium bromide. *Expert Rev Gastroenterol Hepatol* 2014;8(2):131-7. doi: 10.1586/17474124.2014.869477

9. Longstreth GF, Thompson WG, Chey WD, et al. Functional bowel disorders. *Gastroenterology* 2006;130(5):1480-91. doi: 10.1053/j.gastro.2005.11.061

10. Jailwala J, Imperiale TF, Kroenke K. Pharmacologic treatment of the irritable bowel syndrome: a systematic review of randomized, controlled trials. *Ann Intern Med* 2000;133(2):136-47.

11. Liu JP, Yang M, Liu YX, et al. Herbal medicines for treatment of irritable bowel syndrome. *Cochrane Database Syst Rev* 2006(1):CD004116. doi: 10.1002/14651858.CD004116.pub2

12. Palsson OS, Drossman DA. Psychiatric and psychological dysfunction in irritable bowel syndrome and the role of psychological treatments. *Gastroenterol Clin North Am* 2005;34(2):281-303. doi: 10.1016/j.gtc.2005.02.004

13. Song DS, Ryu BH, Park DW, et al. Experimental studies on the efficacy of Samryungbaekchulsan. *J Korean Med* 1993;14(1):179-95.

14. Park JK, Choi SY, Koh KW, et al. Combined utilization with herbal products and prescribed drugs: A result from health examinee-based national survey. *Korean J Health Policy Adm* 2006;16(1):1-16.

15. Duan W. Observation of sixty-five cases on Irritable Bowel Syndrome treated by Trimebutine and Shenlingbaizhu-san granule [Chinese]. *Journal of Practical Traditional Chinese Medicine* 2010;26(5):320.

16. Chen Y. Clinical observation of irritable bowel syndrome treated by shenlingbaizhu-san granule and trimebutine [Chinese]. *Journal of Practical Traditional Chinese Medicine* 2011;27(5):318-9.

17. Li Z, Jiang J. Clinical observation of 80 cases on diarrhea - predominant irritable bowel

For peer review only - http://bmjopen.bmj.com/site/about/guidelines.xhtml

syndrome treated by Shenlingbaizhu-san granule [Chinese]. *Guiding Journal of Traditional Chinese Medicine and Pharmacy* 2013;20(5):41-2.

18. Shin B, Lee B. Frequency Analysis of Clinical Prescriptions in the Korean Medicine Hospital, Pusan National University based on Herb Weight Ratio(2) - Focusing on Prescriptions of Herbal Formula Study Textbook[Korean]. *Herbal Formula Science* 2015;23(1):77-89. doi: dx.doi.org/10.14374/HFS.2015.23.1.077

19. Martinez-Cutillas M, Gil V, Gallego D, et al. Mechanisms of action of otilonium bromide (OB) in human cultured smooth muscle cells and rat colonic strips. *Neurogastroenterol Motil* 2013;25(12):e803-12. doi: 10.1111/nmo.12206

20. Traini C, Cipriani G, Evangelista S, et al. Chronic treatment with otilonium bromide induces changes in L-type Ca(2)(+) channel, tachykinins, and nitric oxide synthase expression in rat colon muscle coat. *Neurogastroenterol Motil* 2013;25(11):e728-39. doi: 10.1111/nmo.12197

21. Traini C, Evangelista S, Girod V, et al. Repeated otilonium bromide administration prevents neurotransmitter changes in colon of rats underwent to wrap restraint stress. *J Cell Mol Med* 2017;21(4):735-45. doi: 10.1111/jcmm.13016

22. Chmielewska-Wilkon D, Reggiardo G, Egan CG. Otilonium bromide in irritable bowel syndrome: a dose-ranging randomized double-blind placebo-controlled trial. *World J Gastroenterol* 2014;20(34):12283-91. doi: 10.3748/wjg.v20.i34.12283

23. Clave P, Acalovschi M, Triantafillidis JK, et al. Randomised clinical trial: otilonium bromide improves frequency of abdominal pain, severity of distention and time to relapse in patients with irritable bowel syndrome. *Aliment Pharmacol Ther* 2011;34(4):432-42. doi: 10.1111/j.1365-2036.2011.04730.x

24. Kwon JG, Park KS, Park JH, et al. [Guidelines for the treatment of irritable bowel syndrome]. *Korean J Gastroenterol* 2011;57(2):82-99.

For peer review only - http://bmjopen.bmj.com/site/about/guidelines.xhtml

25. Li C, Zuo M, Wang L, et al. Clinical observation of diarrhea - predominant irritable bowel syndrome treated by Shenlingbaizhu-san and Paroxetine [Chinese]. *Chinese Journal of Traditional Medical Science and Technology* 2010;17(2):154-5.

26. Leung WK, Wu JC, Liang SM, et al. Treatment of diarrhea-predominant irritable bowel syndrome with traditional Chinese herbal medicine: a randomized placebo-controlled trial. *Am J Gastroenterol* 2006;101(7):1574-80. doi: 10.1111/j.1572-0241.2006.00576.x

27. Ko SJ, Han G, Kim SK, et al. Effect of korean herbal medicine combined with a probiotic mixture on diarrhea-dominant irritable bowel syndrome: a double-blind, randomized, placebo-controlled trial. *Evid Based Complement Alternat Med* 2013;2013:824605. doi: 10.1155/2013/824605

28. Whitehead AL, Julious SA, Cooper CL, et al. Estimating the sample size for a pilot randomised trial to minimise the overall trial sample size for the external pilot and main trial for a continuous outcome variable. *Stat Methods Med Res* 2016;25(3):1057-73. doi: 10.1177/0962280215588241

29. Cha BK, Jung MS, Choi CH, et al. The effect of a multispecies probiotic mixture on the symptoms and fecal microbiota in diarrhea-dominant irritable bowel syndrome: a randomized, double-blind, placebo-controlled trial. *J Clin Gastroenterol* 2012;46(3):220-7. doi: 10.1097/MCG.0b013e31823712b1

30. Kim HJ, Vazquez Roque MI, Camilleri M, et al. A randomized controlled trial of a probiotic combination VSL# 3 and placebo in irritable bowel syndrome with bloating. *Neurogastroenterol Motil* 2005;17(5):687-96. doi: 10.1111/j.1365-2982.2005.00695.x

31. Drossman DA, Patrick DL, Whitehead WE, et al. Further validation of the IBS-QOL: a disease-specific quality-of-life questionnaire. *Am J Gastroenterol* 2000;95(4):999-1007. doi: 10.1111/j.1572-0241.2000.01941.x

For peer review only - http://bmjopen.bmj.com/site/about/guidelines.xhtml

32. Lewis SJ, Heaton KW. Stool form scale as a useful guide to intestinal transit time. *Scand J Gastroenterol* 1997;32(9):920-4. doi: 10.3109/00365529709011203

33. Sugaya N, Izawa S, Saito K, et al. Effect of prolonged stress on the adrenal hormones of individuals with irritable bowel syndrome. *Biopsychosoc Med* 2015;9(1):4. doi: 10.1186/s13030-015-0031-7

34. Fadgyas-Stanculete M, Buga AM, Popa-Wagner A, et al. The relationship between irritable bowel syndrome and psychiatric disorders: from molecular changes to clinical manifestations. *J Mol Psychiatry* 2014;2(1):4. doi: 10.1186/2049-9256-2-4

35. Shi J, Tong Y, Shen JG, et al. Effectiveness and safety of herbal medicines in the treatment of irritable bowel syndrome: a systematic review. *World J Gastroenterol* 2008;14(3):454-62.

36. Zhong Y, Wu Y. Forty cases of diarrhea - predominant irritable bowel syndrome treated by Shenlingbaizhu-san granule and Montmorillonite powder [Chinese]. *Jiangxi Journal of Traditional Chinese Medicine* 2013;44(7):45-6.

37. Xiong B. Thirty cases of irritable bowel syndrome treated by Shenlingbaizhu-san granule and Probiotics [Chinese]. *Jiangxi Journal of Traditional Chinese Medicine* 2011;42(5):22-3.

38. Battaglia G, Morselli-Labate AM, Camarri E, et al. Otilonium bromide in irritable bowel syndrome: a double-blind, placebo-controlled, 15-week study. *Aliment Pharmacol Ther* 1998;12(10):1003-10.

39. Evangelista S. Otilonium bromide: a selective spasmolytic for the gastrointestinal tract. *J Int Med Res* 1999;27(5):207-22. doi: 10.1177/030006059902700501

40. Giachetti A. Pharmacological studies on otilonium bromide. *Ital J Gastroenterol* 1991;23(8 Suppl 1):56-9.

For peer review only

For peer review only - http://bmjopen.bmj.com/site/about/guidelines.xhtml

Figure legend

Figure 1. Flow chart of Trial. GIIM: Gastrointestinal internal medicine, FM: Family medicine, D-IBS: diarrhea-irritable bowel syndrome, P: placebo, OB: otilonium bromide, SRS: Samryungbaekchulsan

For peer review only

For peer review only - http://bmjopen.bmj.com/site/about/guidelines.xhtml

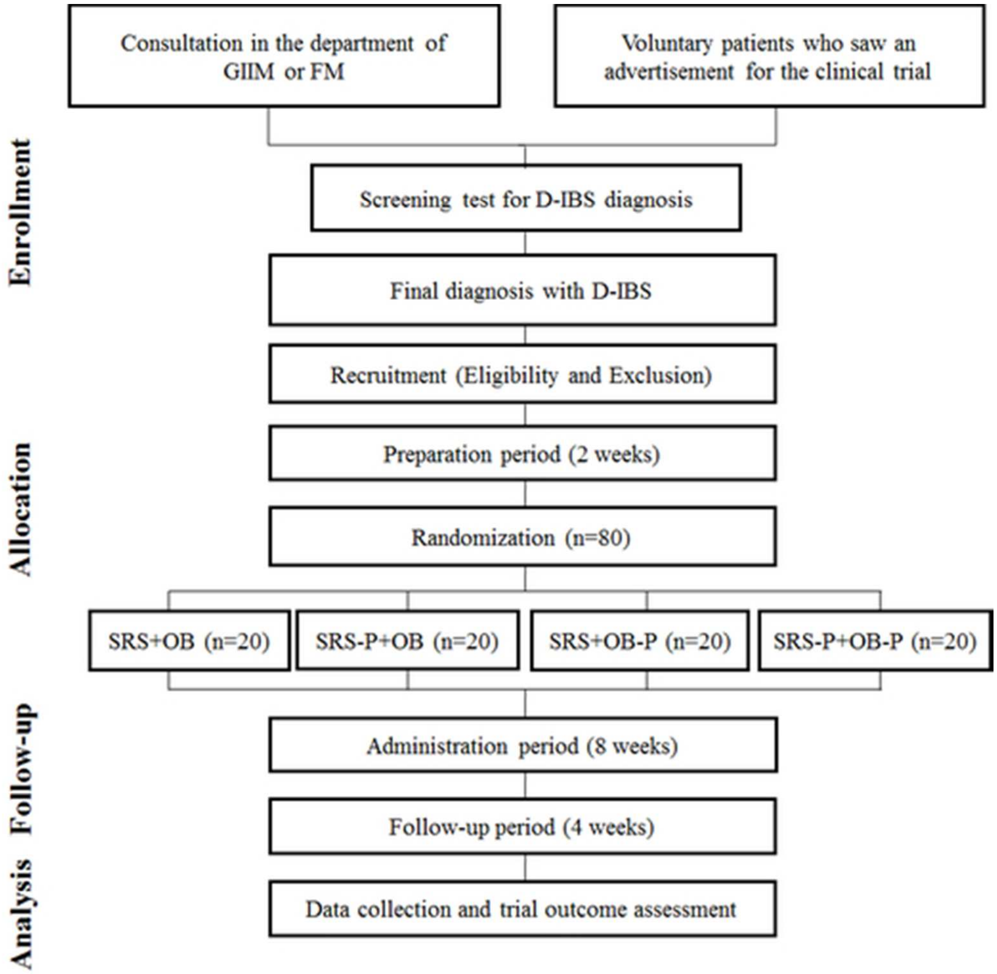

Figure 1. Flow chart of Trial. GIIM: Gastrointestinal internal medicine, FM: Family medicine, D-IBS: diarrhea-irritable bowel syndrome, P: placebo, OB: otilonium bromide, SRS: Samryungbaekchulsan

44x43mm (300 x 300 DPI)

SPIRIT

STANDARD PROTOCOL ITEMS: RECOMMENDATIONS FOR INTERVENTIONAL TRIALS

SPIRIT 2013 Checklist: Recommended items to address in a clinical trial protocol and related documents*

Section/item	Item No	Description	Addressed on page number
**Administrative information**			
Title	1	Descriptive title identifying the study design, population, interventions, and, if applicable, trial acronym	P1
Trial registration	2a	Trial identifier and registry name. If not yet registered, name of intended registry	P4
	2b	All items from the World Health Organization Trial Registration Data Set	Attachment
Protocol version	3	Date and version identifier	P4
Funding	4	Sources and types of financial, material, and other support	P1, P24
Roles and responsibilities	5a	Names, affiliations, and roles of protocol contributors	P1, P23
	5b	Name and contact information for the trial sponsor	P1, P24
	5c	Role of study sponsor and funders, if any, in study design; collection, management, analysis, and interpretation of data; writing of the report; and the decision to submit the report for publication, including whether they will have ultimate authority over any of these activities	N/A
	5d	Composition, roles, and responsibilities of the coordinating centre, steering committee, endpoint adjudication committee, data management team, and other individuals or groups overseeing the trial, if applicable (see Item 21a for data monitoring committee)	P8-9, P11-13, P17-19

For peer review only - http://bmjopen.bmj.com/site/about/guidelines.xhtml

For peer review only

**Introduction**			
Background and rationale	6a	Description of research question and justification for undertaking the trial, including summary of relevant studies (published and unpublished) examining benefits and harms for each intervention	P5-6
	6b	Explanation for choice of comparators	P6
Objectives	7	Specific objectives or hypotheses	P7-8
Trial design	8	Description of trial design including type of trial (eg, parallel group, crossover, factorial, single group), allocation ratio, and framework (eg, superiority, equivalence, noninferiority, exploratory)	P8-9
**Methods: Participants, interventions, and outcomes**			
Study setting	9	Description of study settings (eg, community clinic, academic hospital) and list of countries where data will be collected. Reference to where list of study sites can be obtained	P8
Eligibility criteria	10	Inclusion and exclusion criteria for participants. If applicable, eligibility criteria for study centres and individuals who will perform the interventions (eg, surgeons, psychotherapists)	P9-12
Interventions	11a	Interventions for each group with sufficient detail to allow replication, including how and when they will be administered	P12-14
	11b	Criteria for discontinuing or modifying allocated interventions for a given trial participant (eg, drug dose change in response to harms, participant request, or improving/worsening disease)	P14-17
	11c	Strategies to improve adherence to intervention protocols, and any procedures for monitoring adherence (eg, drug tablet return, laboratory tests)	P14-17,19
	11d	Relevant concomitant care and interventions that are permitted or prohibited during the trial	P14
Outcomes	12	Primary, secondary, and other outcomes, including the specific measurement variable (eg, systolic blood pressure), analysis metric (eg, change from baseline, final value, time to event), method of aggregation (eg, median, proportion), and time point for each outcome. Explanation of the clinical relevance of chosen efficacy and harm outcomes is strongly recommended	P14-16
Participant timeline	13	Time schedule of enrolment, interventions (including any run-ins and washouts), assessments, and visits for participants. A schematic diagram is highly recommended (see Figure)	P8-9, Figure1

For peer review only - http://bmjopen.bmj.com/site/about/guidelines.xhtml

Sample size	14	Estimated number of participants needed to achieve study objectives and how it was determined, including clinical and statistical assumptions supporting any sample size calculations	__P9__________
Recruitment	15	Strategies for achieving adequate participant enrolment to reach target sample size	__P11-12_______

Methods: Assignment of interventions (for controlled trials)

Allocation:

Sequence generation	16a	Method of generating the allocation sequence (eg, computer-generated random numbers), and list of any factors for stratification. To reduce predictability of a random sequence, details of any planned restriction (eg, blocking) should be provided in a separate document that is unavailable to those who enrol participants or assign interventions	__P11-12_______
Allocation concealment mechanism	16b	Mechanism of implementing the allocation sequence (eg, central telephone; sequentially numbered, opaque, sealed envelopes), describing any steps to conceal the sequence until interventions are assigned	__P12__________
Implementation	16c	Who will generate the allocation sequence, who will enrol participants, and who will assign participants to interventions	__P11-12_______
Blinding (masking)	17a	Who will be blinded after assignment to interventions (eg, trial participants, care providers, outcome assessors, data analysts), and how	__P11-12_______
	17b	If blinded, circumstances under which unblinding is permissible, and procedure for revealing a participant's allocated intervention during the trial	__P11-12_______

Methods: Data collection, management, and analysis

Data collection methods	18a	Plans for assessment and collection of outcome, baseline, and other trial data, including any related processes to promote data quality (eg, duplicate measurements, training of assessors) and a description of study instruments (eg, questionnaires, laboratory tests) along with their reliability and validity, if known. Reference to where data collection forms can be found, if not in the protocol	__P17-19_______
	18b	Plans to promote participant retention and complete follow-up, including list of any outcome data to be collected for participants who discontinue or deviate from intervention protocols	__P8-9_________

For peer review only - http://bmjopen.bmj.com/site/about/guidelines.xhtml

Data management	19	Plans for data entry, coding, security, and storage, including any related processes to promote data quality (eg, double data entry; range checks for data values). Reference to where details of data management procedures can be found, if not in the protocol	P17,19
Statistical methods	20a	Statistical methods for analysing primary and secondary outcomes. Reference to where other details of the statistical analysis plan can be found, if not in the protocol	P17-19
	20b	Methods for any additional analyses (eg, subgroup and adjusted analyses)	P17-19
	20c	Definition of analysis population relating to protocol non-adherence (eg, as randomised analysis), and any statistical methods to handle missing data (eg, multiple imputation)	P17-19
**Methods: Monitoring**			
Data monitoring	21a	Composition of data monitoring committee (DMC); summary of its role and reporting structure; statement of whether it is independent from the sponsor and competing interests; and reference to where further details about its charter can be found, if not in the protocol. Alternatively, an explanation of why a DMC is not needed	P19
	21b	Description of any interim analyses and stopping guidelines, including who will have access to these interim results and make the final decision to terminate the trial	P17,19
Harms	22	Plans for collecting, assessing, reporting, and managing solicited and spontaneously reported adverse events and other unintended effects of trial interventions or trial conduct	P15-17
Auditing	23	Frequency and procedures for auditing trial conduct, if any, and whether the process will be independent from investigators and the sponsor	P19
**Ethics and dissemination**			
Research ethics approval	24	Plans for seeking research ethics committee/institutional review board (REC/IRB) approval	P3, P22
Protocol amendments	25	Plans for communicating important protocol modifications (eg, changes to eligibility criteria, outcomes, analyses) to relevant parties (eg, investigators, REC/IRBs, trial participants, trial registries, journals, regulators)	P22

For peer review only - http://bmjopen.bmj.com/site/about/guidelines.xhtml

Consent or assent	26a	Who will obtain informed consent or assent from potential trial participants or authorised surrogates, and how (see Item 32)	__P22-23_____
	26b	Additional consent provisions for collection and use of participant data and biological specimens in ancillary studies, if applicable	__N/A_________
Confidentiality	27	How personal information about potential and enrolled participants will be collected, shared, and maintained in order to protect confidentiality before, during, and after the trial	__P23_________
Declaration of interests	28	Financial and other competing interests for principal investigators for the overall trial and each study site	__P24_________
Access to data	29	Statement of who will have access to the final trial dataset, and disclosure of contractual agreements that limit such access for investigators	__P17_________
Ancillary and post-trial care	30	Provisions, if any, for ancillary and post-trial care, and for compensation to those who suffer harm from trial participation	__P23_________
Dissemination policy	31a	Plans for investigators and sponsor to communicate trial results to participants, healthcare professionals, the public, and other relevant groups (eg, via publication, reporting in results databases, or other data sharing arrangements), including any publication restrictions	__P3, 23_______
	31b	Authorship eligibility guidelines and any intended use of professional writers	__N/A_________
	31c	Plans, if any, for granting public access to the full protocol, participant-level dataset, and statistical code	__N/A_________
**Appendices**			
Informed consent materials	32	Model consent form and other related documentation given to participants and authorised surrogates	__Attachment__
Biological specimens	33	Plans for collection, laboratory evaluation, and storage of biological specimens for genetic or molecular analysis in the current trial and for future use in ancillary studies, if applicable	__P15-16_______

*It is strongly recommended that this checklist be read in conjunction with the SPIRIT 2013 Explanation & Elaboration for important clarification on the items. Amendments to the protocol should be tracked and dated. The SPIRIT checklist is copyrighted by the SPIRIT Group under the Creative Commons "Attribution-NonCommercial-NoDerivs 3.0 Unported" license.

For peer review only

For peer review only - http://bmjopen.bmj.com/site/about/guidelines.xhtml

Effect of herbal extract granules combined with otilonium bromide on irritable bowel syndrome with diarrhea: a study protocol for a randomized controlled trial

Journal:	*BMJ Open*
Manuscript ID	bmjopen-2017-018362.R3
Article Type:	Protocol
Date Submitted by the Author:	02-Nov-2017
Complete List of Authors:	Kim, Joong Il; Catholic Kwandong University International Saint Mary's Hospital, Institute for Integrative Medicine;<br>Kim, Pumsoo; Catholic Kwandong University International Saint Mary's Hospital, Institute for Integrative Medicine<br>Lee, Jin-Hyun; Wonkwang University College of Korean Medicine, Department of Korean Rehabilitation Medicine<br>Kim, Yoo-Jin; Catholic Kwandong University International Saint Mary's Hospital, Institute for Integrative Medicine<br>Yang, Na-rae; Catholic Kwandong University International Saint Mary's Hospital, Institute for Integrative Medicine<br>Baeg, Myong Ki; Catholic Kwandong University International Saint Mary's Hospital, Institute for Integrative Medicine<br>Choi, Ja Sung; Catholic Kwandong University International Saint Mary's Hospital, Institute for Integrative Medicine<br>Kim, Hye-Jung; Catholic Kwandong University International Saint Mary's Hospital, Institute for Integrative Medicine<br>Kim, Ja-Young; Catholic Kwandong University International Saint Mary's Hospital, Institute for Integrative Medicine<br>Sunwoo, Yun-Young; Catholic Kwandong University International Saint Mary's Hospital, Institute for Integrative Medicine<br>Lee, Jung-Han; Won-Kwang University, Department of Rehabilitation Medicine, College of Korean Medicine<br>Ha, Hyekyung; Korea Institute of Oriental Medicine, K-herb Research Center<br>Park, Tae-Yong; Catholic Kwandong University International Saint Mary's Hospital, Institute for Integrative Medicine
**Primary Subject Heading**:	Complementary medicine
Secondary Subject Heading:	Gastroenterology and hepatology
Keywords:	Samryungbaekchulsan, otilonium bromide, irritable bowel syndrome, randomized controlled study, clinical trial protocol

SCHOLARONE™
Manuscripts

For peer review only

Effect of herbal extract granules combined with otilonium bromide on irritable bowel syndrome with diarrhea: a study protocol for a randomized controlled trial

Joong Il Kim[1*], Pumsoo Kim[1*], Jin-Hyun Lee[2], Yoo-Jin Kim[1], Na-rae Yang[1], Myong Ki Baeg[1], Ja Sung Choi[1], Hye-Jung Kim[1], Ja-Young Kim[1], Yun-Young Sunwoo[1], Jung-Han Lee[2], Hyekyung Ha[3], Tae-Yong Park[1]

[1] *Institute for Integrative Medicine, Catholic Kwandong University International St. Mary's Hospital, Incheon, 22711, South Korea*

[2] *Department of Rehabilitation Medicine, College of Korean Medicine, Wonkwang University, Iksan, 54383, South Korea*

[3] *K-herb Research Center, Korea Institute of Oriental Medicine, Daejeon, 34054, South Korea*

Corresponding author

Tae-Yong Park

Address: Institute for Integrative Medicine, Catholic Kwandong University International, St. Mary's Hospital, Simgok-ro 100gil 25, Seo-gu, Incheon, 22711, Republic of Korea

E-mail: parktae9822@gmail.com

Telephone: +82-32-290-3319

*** These authors contributed equally to the study**

Funding: This work was supported by a "Construction of Scientific Evidences for Herbal Medicine Formulas (K15250)" grant from the Korea Institute of Oriental Medicine (KIOM). (Telephone: +82-42-868-9513)

Word Count: 5898

For peer review only

Keywords: Samryungbaekchulsan, otilonium bromide, irritable bowel syndrome, randomized controlled study, clinical trial protocol

For peer review only

For peer review only - http://bmjopen.bmj.com/site/about/guidelines.xhtml

ABSTRACT

Introduction

Irritable bowel syndrome (IBS), known as a functional and organic gastrointestinal (GI) disorder, is a collection of symptoms that occur together and generally include pain or discomfort in the abdomen and changes in bowel movement patterns. Due to the limitations of conventional treatments, alternative IBS treatments are used by many patients worldwide. Samryungbaekchulsan (SRS), a herbal formula, has long been used for alleviating diarrhea-predominant IBS (D-IBS) in traditional Korean medicine. Otilonium bromide (OB) is an antimuscarinic compound used to relieve spasmodic pain in the gut, especially in IBS. Although herbal formulas and western drugs are commonly co-administered for various diseases in Korea, few clinical studies have been conducted regarding the synergic effects of these treatments for any disease, including D-IBS.

Methods and Analysis

This trial is a randomized, double-blinded, placebo-controlled, double-dummy, four-arm, parallel study. After a 2-week preparation period, 80 patients with D-IBS will be randomly assigned to one of four treatment groups consisting of SRS (water extract granules, 5 g/pack, 3 times a day) with OB (tablet form, 1 capsule 3 times a day) or their placebos, with treatment lasting for 8 weeks. Post-treatment follow-up will be conducted 4 weeks after the end of treatment. The primary outcome is the finding obtained using the Subject's Global Assessment of Relief method. The secondary outcomes are the severity of symptoms related to D-IBS, determined using a 10-point scale, and the change in symptoms.

Ethics and dissemination

This trial has full ethical approval of the ethics committee of Catholic Kwandong University International St. Mary's Hospital (IS15MISV0033) and the Korean Ministry of Food and Drug Safety (30769). The results of the study will be disseminated through a peer-reviewed journal and/or conference presentations.

For peer review only - http://bmjopen.bmj.com/site/about/guidelines.xhtml

Trial protocol version: IS15MISV0033 Version 4.0 (July 25, 2016)

Trial registration number: KCT0001621 (Approval date: Aug 10, 2015)

STRENGTHS AND LIMITATIONS OF THIS STUDY

■This randomized controlled trial is the first Korean clinical trial approved by the Korean Ministry of Food and Drug Safety (KMFDS) to investigate the efficacy and safety of Samryungbaekchulsan (SRS; herbal formula) and otilonium bromide (OB; western drug) in diarrhea-predominant irritable bowel syndrome (D-IBS).

■To evaluate the therapeutic effect and safety of the co-administration of SRS and OB, this study is proposed as a double-blind, double-dummy, four-arm, parallel-group randomized controlled trial.

■The primary outcome is the improvement in the patient's symptoms according to the Subject's Global Assessment of Relief and the secondary outcome is the severity of D-IBS symptoms.

■This study is conducted with a small sample size (four arms, 20 participants per group), so further clinical trials with powerful sample sizes will be required to more adequately determine the effectiveness of SRS and/or OB for D-IBS.

For peer review only - http://bmjopen.bmj.com/site/about/guidelines.xhtml

INTRODUCTION

Irritable bowel syndrome (IBS), a chronic gastrointestinal (GI) disease and functional bowel disorder, is characterized by abnormal bowel habits and abdominal pain without structural, morphological, and histological abnormalities. It affects approximately 20% of the population worldwide, according to population-based studies[1-6]. In addition, recent evidence suggests that IBS is not only a functional disease, but also an organic disease with a complex of symptoms, including infection, immune activation, serotonin dysregulation, bacterial overgrowth, central dysregulation, brain-gut interaction, and genetics[4-8]. Based on their predominant bowel habits, patients with IBS can be divided into diarrhea-predominant IBS (D-IBS), constipation-predominant IBS, or mixed IBS[2 6 9]. Recently, smooth-muscle relaxants, bulking agents, and anti-diarrheal agents have been used in the treatment of IBS[10]. However, these methods are often not effective and many IBS patients also use complementary treatments, such as herbal medicine, acupuncture, psychological treatment, and lifestyle correction[2 11 12].

Samryungbaekchulsan (SRS; Shenlingbaizhu-san in Traditional Chinese Medicine; Jinryobyakujutsu-san in Kampo Medicine), which was introduced in the famous classical compendium of herbal formulas "Formulary of Peaceful Benevolent Dispensary," consists of 10 herbs (Atractylodes rhizoma Alba, Poria sclerotium, Dioscoreae rhizoma, Glycyrrhizae radix et rhizoma, Coicis semen, Nelumbinis semen, Platycodonis radix, Dolichoris semen, Amomi fructus, and Ginseng radix). In traditional Korean and Chinese medicine, this herbal formula has been used to treat GI disease, and various studies have been published regarding the treatment of IBS with SRS[13-18]. SRS has been shown to have anti-acetylcholine and anti-barium chloride effects and to suppress gastric secretion and small intestine motility[13]. However, no clinical trials have evaluated the efficacy of SRS for IBS when co-administered with a conventional drug.

For peer review only - http://bmjopen.bmj.com/site/about/guidelines.xhtml

Otilonium bromide (OB) is widely used for the prevention and reduction of symptoms in patients with IBS[7 8]. OB has been shown to inhibit L- and T-type calcium channels, muscarinic receptors, and tachykininergic responses in human cultured smooth muscle cells and rat colon strips[19-21]. According to a recent randomized controlled trial (RCT), OB (40 and 80 mg) is more effective for treating IBS than placebo, not only for the reduction of bloating and abdominal pain, but also for protection from relapse owing to its long-lasting effect[22 23].

According to the guidelines of the Korean Society of Gastroenterology, antispasmodic agents are recommended for the treatment of D-IBS (Grade 1B),[24] and SRS is the preferred drug for diarrhea treatment in Korean medicine[18]. Recently, several studies have been conducted to evaluate the therapeutic effect of the co-administration of herbal formulas and conventional drugs for IBS in China[25 26]. However, there has been no such clinical study in Korea, even though both herbal medicine and conventional drugs are widely used for IBS treatment in the clinical field. Therefore, there is a need to evaluate the efficacy and safety of SRS, OB, and the co-administration of both drugs.

In this study, we expect that the combined administration of OB and SRS will result in synergistic effects that improve D-IBS symptoms, including abdominal pain, discomfort (the main effect of OB), and diarrhea-like stool patterns (the main effect of SRS).

For peer review only - http://bmjopen.bmj.com/site/about/guidelines.xhtml

For peer review only

METHODS AND ANALYSIS

Objectives

The purpose of this study is to evaluate the efficacy and safety of the co-administration of SRS and OB to patients with D-IBS.

Necessity of the trial

Recently, herbal medicines and western drugs have been co-administered for various medical therapies. This is particularly true in Korea, where approximately 76% of patients have used the combination of herbal medicines and western drugs[14]. However, only a few studies have evaluated the interaction between western and herbal medicines. Despite the need for combined treatment in patients with various diseases, there are no standard clinical guidelines or scientific evidence regarding co-administration because of a lack of research and data.

D-IBS is a disease that is difficult to cure and many patients require treatment with both western and herbal drugs to experience an improvement in their symptoms. Therefore, it is very important to conduct a clinical study regarding the efficacy and safety of the combined administration of SRS and OB in patients with D-IBS.

Basis of drug selection

According to guidelines for IBS treatment, the OB recommendation level is high (i.e., the level of evidence is moderate and many experts recommend using it) and it is the most reasonable treatment option[25]. SRS is the herbal formula used most often by clinical Korean and Chinese medical doctors for abdominal pain, abdominal discomfort, and chronic diarrhea, which are typical symptoms of D-IBS[15-17 24]. Based on these studies and clinical applications, OB and SRS were selected for this trial.

For peer review only - http://bmjopen.bmj.com/site/about/guidelines.xhtml

Hypothesis

We hypothesize the following: (1) that co-administration of SRS and OB will have a more positive effect on D-IBS symptoms than either SRS or OB alone; (2) that after 8 weeks of treatment with SRS, OB, or both drugs, the Subject's Global Assessment of Relief and severity of D-IBS symptoms will be more improved than after treatment with placebo; and (3) that co-administration of SRS and OB will be safe for patients with D-IBS.

Design

This study is a placebo-controlled, double-blind trial. Eighty patients will be enrolled and randomly assigned to four separate arms of the trial. The trial will be conducted at Catholic Kwandong University International St. Mary's Hospital, Incheon, South Korea.

The study subjects will be required to participate in a 2-week preparation period (weeks -2 to 0) and an 8-week drug administration period (weeks 0 to 8). The patients will visit four times, on weeks 0, 4, 8, and 12. The primary outcome measurement will be evaluated at weeks 2, 4, 6, 8, and 12, and the secondary outcome measurement will be evaluated at weeks 0, 2, 4, 6, 8, and 12. Participants will be assessed for efficacy and safety of the treatment through visits (0, 4, 8, 12 weeks) and telephone questionnaires (2, 6 weeks). In addition, lab tests will be conducted at 0, 4, and 8 weeks. According to the intervention protocols, the clinical research coordinator (CRC) will encourage patients and participants to complete follow-up at every visit.

During the drug administration period, SRS or its placebo will be given as 1 pack of water extract granules 3 times a day, 30 min before each meal. OB or its placebo will be given as 1 tablet 3 times a day, 30 min before each meal.

Participants will be divided into 4 groups: (1) SRS and OB group; (2) SRS and placebo-

For peer review only - http://bmjopen.bmj.com/site/about/guidelines.xhtml

OB group; (3) placebo-SRS and OB group; and (4) placebo-SRS and placebo-OB group. The entire flow chart of the study is shown in Figure 1.

The protocol of this trial has been approved by the institutional review board (IRB) and ethics committee of Catholic Kwandong University International St. Mary's Hospital. The IRB permission number is IS15MISV0033 and CRIS (Clinical Research Information Service, www.cris.nih.go.kr) protocol identification number is KCT0001621. In addition, this study received the first approval given by the Korean Ministry of Food and Drug Safety (KMFDS) for a study of the co-administration of a conventional drug and a Korean traditional herbal medicine (approval number: 30769).

Potential participants will be provided with sufficient information about the study and only those who agree to the study protocol and sign the consent form will be enrolled. The study will be implemented according to Good Clinical Practice and the revised version of the Declaration of Helsinki.

Sample size calculation

This trial is a study for a new therapeutic regimen of SRS and OB combination treatment. In a similar study conducted previously by using co-administration of a probiotics mixture and an herbal medicine (placebo-controlled, double-blind, and randomly 4-arm), each arm consisted of at least 15 patients and the total sample size was more than 60 participants[27]. In other similar studies, clinical trials have been conducted with a total of 60–64 participants, consisting of 12–30 patients per group[28-30]. Based on these previous studies, the minimal clinical significance of the trial can be obtained when only 16 participants complete the trial. Because the drop-out rate is expected to be 20%, each trial group will be made up of at least 20 participants. Therefore, the total sample size must be more than 80 participants.

For peer review only - http://bmjopen.bmj.com/site/about/guidelines.xhtml

Inclusion criteria

The following patients would be included: (1) patients aged 18–75 years; (2) patients satisfying the Rome III criteria of IBS (recurrent abdominal pain or discomfort that began at least 6 months before the clinical trial, with a rating of more than 3 on a 10-point scale, frequency of at least 3 days/month in the last 3 months, and associated with two or more of the following: improvement associated with defecation, onset associated with a change in frequency of stools, and onset associated with a change in form of stool)[1 9]; (3) patients satisfying the Rome III criteria of IBS, whereby D-IBS type is defined as loose/mushy or watery stools—Bristol Stool Form (BSF) Scale 6-7 ≥ 2 times/week[1 30]; (4) patients with a negative urine pregnancy test (in women of a childbearing age, within 7 days before the clinical trial) and who consent to using contraception during the administration period; (5) patients with the ability to read the symptom questionnaire and understand it; and (6) patients who agree with the clinical plan and voluntarily sign the IRB-approved documents.

Exclusion criteria

The exclusion criteria are as follows: (1) chronic liver disease (cirrhosis, chronic hepatitis B or C); (2) chronic renal failure or renal impairment (serum creatinine ≥ 2 times the upper limit of normal); (3) liver dysfunction (AST/ALT ≥ 3 times the upper limit of normal); (4) diabetes (HbA1c > 8% or not controlled by diet or medication), hypertension (≥ 160/100 mmHg), thyroid dysfunction (exceptions in the following cases: if the disease is controlled by drug administration with a stable dose for 12 weeks before the screening, and the drug dose remains constant during the trial period), clinically significant hematologic, cardiac, pulmonary, neurological disorders, or other severe systemic disorders; (5) abnormal findings on colonoscopy or colonography within the last 5 years; (6) history of surgery that affects gastrointestinal motility (i.e., gastrectomy, colonic resection, hysterectomy, except for

For peer review only - http://bmjopen.bmj.com/site/about/guidelines.xhtml

appendectomy); (7) GI disease characterized by the following symptoms within 6 months before the clinical trial: inflammation of or ulcer in the esophagus, stomach, or duodenum; gastroesophageal reflux disease (at the discretion of the examiner, subjects may enroll if the disease is chronic and not acute); gastrointestinal bleeding; gastrointestinal stenosis or closure; infectious diarrhea; inflammatory bowel disease (i.e., Crohn's disease, diverticulitis, ulcerative colitis, infectious enteritis, ischemic colitis); pancreatic insufficiency; biliary abscess; (8) mental illness or an addiction to drugs or alcohol; (9) severe systemic organ diseases such as cancer, autoimmune disease, stroke (although patients in remission for more than 5 years from a cancer unrelated to the GI tract can participate in the trial); (10) pregnancy, breastfeeding, and unwillingness to use contraception during the trial; (11) a history of taking the following drugs within 2 weeks before the trial: antibiotics, analgesics, antidepressants, anti-anxiety agents, anti-inflammatory agents, anti-ulcer agents, anti-gastric secretion inhibitors, laxatives, antispasmodics, antacid agents, gastrointestinal stimulants, prostaglandin agents, and corticosteroids; (12) use of medications that do not match the intent of this trial or have a clinical interaction with SRS or OB; (13) lactose intolerance (not controlled by food); (14) glaucoma; (15) participation in other clinical studies within 30 days prior to clinical screening and have received other clinical trial medications (including placebo); (16) hypersensitivity to clinical trial medicines; and (17) other reasons considered inappropriate for participation in clinical trials.

Recruitment, randomization, blinding, and unblinding

Advertisements for the study will be posted at the Catholic Kwandong University International St. Mary’s Hospital using banner ads and posters. We also plan to post the advertisements on the hospital homepage, in the monthly hospital magazine, at the internet café, and on the subway at least five times. All these advertisements will be done with the

For peer review only - http://bmjopen.bmj.com/site/about/guidelines.xhtml

approval of the IRB.

After recruitment, patients will be randomized to one of the four trial arms. Randomization will be performed using a list of block randomizations made by statisticians, independent of this clinical trial. A researcher who is not involved in process of drug prescription and evaluation will execute the randomization process.

During the treatment period, the patients and all researchers (investigators and clinical research coordinators) will be blinded, except the researchers who conducted the randomization procedure. In addition, the researchers will not know the kind of medication that is being administered to the patient or any other information that could lead to bias.

Allocation concealment will be performed during the process of medication administration. The management pharmacist, who will not be involved in analyzing the results of the trial, will use the randomization table to label the medication with the assignment number and provide the medication. The patients will take the drugs contained in the opaque envelope, which will be labeled with the assignment number.

Unblinding will be performed only in the following situations: (1) if the trial is terminated and there is a need for statistical analysis; (2) if a serious medical emergency occurs and information about the medication is needed; and (3) if the chief investigator determines that unblinding is necessary.

The entire process of the study will be directed by the authorized clinical research organization (CRO), Dream CIS Corporation, Seoul, Korea.

Intervention

The herbal formula SRS is commonly used by Korean traditional medical doctors for treatment of diarrhea and related diseases[13-18]. SRS used in this study (Samryungbaekchulsan granule®, Hankook shin yak, Co., Ltd., Nonsan, Korea) is extracted with water and mixed

For peer review only - http://bmjopen.bmj.com/site/about/guidelines.xhtml

with starch and lactose in accordance with Korean Good Manufacturing Practice (K-GMP). Production of SRS is regulated and allowed by the KMFDS. SRS contains 10 herbs, listed in Table 1. Placebo-SRS is made from cornstarch and has the same taste, shape, color, and similar scent as SRS. SRS and placebo-SRS will be sealed in identical aluminum bags with the same labeling. These herbal drug packages will be distributed by an independent pharmacist in an isolated room. According to the recommended dosing methods of KMFDS, after SRS or placebo-SRS is dissolved in boiled water, the subjects will take them 30 min before each meal (5 g/pack, 3 times a day).

OB (Menoctyl Tab®, Dong Hwa Pharm Co., LTD., Seoul, Korea) is a tablet containing otilonium bromide 40 mg. Placebo-OB is a tablet made of Ludipress with the same taste, shape, and color as OB. Patients will be instructed to swallow one tablet of OB or placebo-OB 30 min before each meal over the administration period. The drugs used in the trial (SRS, OB, placebo-SRS, placebo-OB) have been approved by the KMFDS. Patients will be asked at the end of the study whether the drugs that they have been taking are either real or placebo, to measure the success of blinding. Compliance will be confirmed by counting returned SRS packets or OB tablets. Every patient will be instructed to write down any adverse events during the administration period and these records will be evaluated at the follow-up. At the patient visits (at 0, 4, and 8 weeks), we will perform a check of vital signs, physical examination, electrocardiogram, and laboratory tests to evaluate safety for the patients. All side effects will be reported to the investigator.

For peer review only

For peer review only - http://bmjopen.bmj.com/site/about/guidelines.xhtml

Table. 1 Ingredients in the herbal formula Samryungbaekchulsan (SRS)

Scientific name	Part used	Grams/day
Atractylodis Rhizoma Alba	Root	3.0
Poria Sclerotium	Dried core	4.0
Rhizoma Dioscoreae	Root	3.0
Glycyrrhizae Radix et Rhizoma	Root	1.5
Semen Coicis	Seed	8.0
Semen Nelumbinis	Fruit	4.0
Platycodonis Radix	Root	2.5
Dolichoris Semen	Seed	4.0
Amomi Fructus	Fruit	2.0
Ginseng Radix	Root	3.0

Rescue therapy and concomitant medications

Participants are prohibited from taking any medication that could affect D-IBS during the entire course of the trial. However, if serious side effects occur or symptoms worsen, investigators will provide quick and appropriate treatment (including drug administration), based on their medical judgement. In this case, the direct investigators will record the treatment or drug administration and report to the IRB within 24 h to determine whether the trial should be continued or discontinued.

Outcome Measurements

Primary outcome

The primary outcome will be measured via a questionnaire that confirms subjective

For peer review only - http://bmjopen.bmj.com/site/about/guidelines.xhtml

symptom improvement. Participants will respond a total of five times during and after the administration period (at 2 and 6 weeks via telephone, and at 4, 8, and 12 weeks during direct visits) to the following question: Subject's Global Assessment of Relief; "How much do you think the symptoms of D-IBS have improved compared to before the clinical trial?" According to the answers given by the patients to this survey, the improvement is given a score of 0, 1, 2, 3, or 4.

Secondary outcomes

At 2, 4, 6, 8, and 12 weeks from the start of the treatment period, patients will be assessed for severity of D-IBS symptoms (abdominal pain, abdominal discomfort, satisfaction of defecation, frequency of abdominal pain, quality of life) using a Likert scale ranging from 0 to 10[31]. Patients will also be instructed to record the number of defecations per day, BSF, and the degree of force used in bowel movements at 0, 2, 4, 6, 8, and 12 weeks[32].

Safety assessments

The investigator will record all adverse events that occur during the trial along with any concomitant medications in the case reports. When an adverse event occurs, the investigator will record the symptoms and signs of the adverse reaction, the duration (start and end date), severity, course, outcome, significance, causality by the trial drug, and any action taken in relation to the adverse event. In the case of concomitant medicines, composition, dosage, duration of administration, and reason for medication will be recorded in detail. Symptoms that existed before the start of the clinical trial will not be recorded as adverse events.

Laboratory tests

The purpose of laboratory tests in this study is to evaluate the safety and pharmacokinetic

For peer review only - http://bmjopen.bmj.com/site/about/guidelines.xhtml

profiles of SRS and OB. Because of the small number of patients assigned to this study and the different baseline values for each individual patient, we will not perform a detailed statistical analysis. However, if significant abnormal results are found in the laboratory tests, the clinical laboratory results will be described and the connection to the drug will be considered.

Blood tests will be conducted at 0, 2, 4, and 8 weeks and urine tests will be performed at 0 and 4 weeks during the clinical trial. In the case of a participant who drops out or the early termination of the clinical trial, blood and urine tests will be performed on the last day of the trial.

Using the test results (comprehensive verification of vital signs, laboratory tests, electrocardiograms, and physical examinations) for each individual patient, the investigator will determine adverse reactions caused by the drugs and statistical tests will be conducted as needed on clinically significant parameters.

Test of human derivatives

IBS is known to be closely related to stress[12]. Therefore, we plan to quantify factors associated with stress or that have been reported to worsen IBS symptoms as follows: 1) cortisol; 2) corticotropin-releasing hormone (CRH); 3) serotonin; 4) group I cytokines (10 species); 5) group II cytokines (14 species); and 6) growth factors (3 species)[33 34]. Serum will be separated from blood specimens collected at 0, 4, 8 weeks, stored frozen, and transferred to the Korea Institute of Oriental Medicine (KIOM) for examination.

Early termination or dropout

The criteria for early termination or dropout are as follows: 1) administration of other drugs that are expected to affect the safety and efficacy of the clinical trial drugs; 2) request

For peer review only - http://bmjopen.bmj.com/site/about/guidelines.xhtml

of participants to discontinue the clinical trial, or withdrawal of the trial agreement; 3) occurrence of significant adverse drug reactions or events that preclude continuing the trial, according to the judgment of the clinical investigator; 4) new discovery of violations of significant clinical trial protocol during clinical trial; 5) less than 70% trial drug compliance; and 6) any other reason that the clinical trial should be discontinued at the discretion of the clinical trial manager or investigator.

Data collection, access, and management

The data of this trial will be managed according to the standard work instructions of Catholic Kwandong University International, St. Mary's Hospital. Other contents not specified in the trial protocol shall follow the standards of The International Conference on Harmonization of Technical Requirements for Registration of Pharmaceuticals for Human Use (ICH) Guideline for Good Clinical Practice (GCP) and Korean Good Clinical Practice (KGCP) guideline.

The source document is recorded immediately when the data are collected. After the source document input is completed, it will be recorded in the case record form (CRF). All the documents will be kept safe, so that the data can be verified by the relevant government agencies and the IRB. Only research colleagues and others who have been approved by the principle investigator will have access to all data obtained from this trial.

Statistical analysis

The statistical analysis will be conducted by blinded professional statisticians. To evaluate the efficacy of the study, both an intent-to-treat (ITT) analysis (primary analytical method) and a per-protocol (PP) analysis (secondary analytical method) will be performed. The ITT analysis will be conducted for all patients who were followed up at least once after

For peer review only - http://bmjopen.bmj.com/site/about/guidelines.xhtml

randomization and will be able to be evaluated for effectiveness. The PP analysis will be performed for patients who complete the 8-week treatment course and meet the inclusion and exclusion criteria.

To analyze the continuous variables in the demographic information and pre-treatment characteristics between groups, a one-way analysis of variance (ANOVA) and Kruskal-Wallis test will be conducted. An ANOVA and Kruskal-Wallis test will also be used to test for symptom improvement compared to baseline (0 week) and the variables for analysis that include the following: (1) subjective symptom improvement (primary outcomes); and (2) severity of symptoms related to D-IBS (abdominal pain, abdominal discomfort, satisfaction of defecation, frequency of abdominal pain, quality of life). All values will be shown as mean ± standard error of the mean (SEM). Comparisons of outcome assessments between the four groups will be performed using the paired t-test or Wilcoxon signed-rank test.

If the Subject's Global Assessment of Relief on the Likert scale is "0" or "1" for more than 2 weeks between weeks 8 and 12, then the symptoms will be considered to have improved sufficiently. Based on this, we will calculate the symptom relief rate by dividing the number of participants who exhibited sufficient symptom relief by the total number of participants in the trial and present descriptive statistics. The CATMOD procedure will be used to evaluate the relief rate improvement of symptoms compared with baseline. Comparisons between the four groups will be conducted using the chi-square test or Fisher's exact test. These statistical analyses will also be performed at 2, 4, 6, 8, and 10 weeks from baseline. The Cochran-Mantel-Haenszel (CMH) test will be used to confirm the difference between the intervention groups at each time-point.

A statistical analysis will also be conducted to evaluate safety. The incidence of adverse events and abnormality of experimental results will be analyzed statistically according to the groups and non-parametric methods will be applied as necessary using a paired t-test for

For peer review only - http://bmjopen.bmj.com/site/about/guidelines.xhtml

continuous data, McNemar's test for categorical data, Fisher's exact test for adverse events, and a generalized estimating equation for clinically significant changes between groups.

All statistical analysis will be conducted using SAS software, version 9.1.3 (SAS institute, Cary, NC). A P-value < 0.05 will be considered statistically significant.

Quality control and data monitoring

To ensure the results and quality of the clinical trial, assessments and monitoring will be performed by the CRO, DreamCIS Corporation (Seoul, Korea), which is independent from the sponsor and competing interests. During the trial, the CRO will regularly monitor whether the study is proceeding in accordance with the protocol through the use of all related documentation, including trial master files, case report forms, informed consent forms, and adverse event reports.

DISCUSSION

Herbal medicine is a recognized effective treatment method based on clinical experience accumulated over thousands of years and more recently gained attention as an alternative therapy for treating various diseases that are difficult to treat or have unknown causes, such as IBS[5 7 10 26 35]. SRS is frequently used for abdominal pain and diarrhea in Korean and Chinese traditional medicine. According to experimental research, SRS has an anti-cholinergic effect that improves intestinal digestion and absorption and inhibits intestinal movement, resulting in the prevention of diarrhea and abdominal pain[13]. In addition, clinical studies have shown that the use of SRS in patients with D-IBS is effective for improving diarrhea[36 37]. Some studies have reported that co-administration with SRS and trimebutine maleate or SRS and paroxetine is more effective than trimebutine maleate alone for the improvement in IBS symptoms[15 16 25]. Other studies have reported that SRS exerted more

For peer review only - http://bmjopen.bmj.com/site/about/guidelines.xhtml

beneficial effects on IBS patients compared with Smecta or probiotics[17 37].

However, although there is a growing interest in the use of herbal medicines such as SRS in IBS and related studies are continuously being published, there have been only a few well-designed RCTs[22 23 26]. To overcome the limitations of the previous studies, this trial was designed as a double-blind, double-dummy, four-arm, parallel-group RCT. The efficacy of SRS will be evaluated via the Subject's Global Assessment of Relief and the improvement in symptoms associated with bowel movements and abdominal pain. The results of these assessments are expected to provide distinct clinical evidence for the use of SRS in D-IBS treatment.

OB is a representative conventional medicine used for D-IBS treatment[8 21 23 38 39]. It is a spasmolytic agent that exerts its activity primarily in the distal GI tract through the inhibition of Ca++ flux and direct activation of contractile proteins in the smooth muscle[20 21]. OB has also been shown to reduce hyper-motility and modulate visceral sensation, the effect of which is to improve D-IBS symptoms[39 40]. RCTs using OB for the treatment of D-IBS have been conducted. In these studies, treatment with OB resulted in a greater improvement in symptoms related to D-IBS (defecation frequency, regular intestinal habits, reductions in frequency of diarrhea, abdominal pain and discomfort, severity of abdominal bloating) and better protection from symptom relapse compared to placebo[22 23 38].

IBS is a disease with a high rate of progression and frequent recurrence. For this reason, patients with IBS often require a variety of treatment methods[2 6 10]. Among these methods, the combination of herbal and conventional drugs is widely used and is more effective for improving IBS symptoms than conventional drugs alone[2 35]. However, there are several limitations to the co-administration of herbal and conventional drugs in IBS. Specifically, there is a high risk of bias in related studies and there are insufficient clinical guidelines regarding the interaction of herbal and conventional drugs in D-IBS, including SRS and OB.

For peer review only - http://bmjopen.bmj.com/site/about/guidelines.xhtml

Therefore, it is necessary to perform well-designed RCTs to evaluate the co-administration of herbal and conventional drugs.

Because only a few studies have evaluated the combination of herbal and conventional drugs, our proposed study will significantly contribute to overcoming the limitations of previous studies and provide basic medical evidence for the co-administration of SRS and OB for D-IBS treatment. Therefore, this RCT has several characteristic features. First, this trial will be the first to investigate the efficacy of SRS and OB combination therapy for D-IBS. The results of previous studies suggested that the combination of OB and SRS will improve two major D-IBS symptoms at the same time: abdominal pain and discomfort (major effect of OB) and diarrhea (main effect of SRS). This study will assess this hypothesis and provide a basis for the use of SRS and OB combination therapy for D-IBS. Second, the participants in this trial will be grouped into four arms, making it possible to compare the efficacy of combined treatment with the efficacy of each individual treatment. Third, this trial will evaluate the safety of the subjects using several assessments, including a periodic assessment of subjective symptoms, physical measurements, and laboratory testing. The results of the safety analysis may provide evidence for the safety of treatment with the combination of herbal and conventional drugs in D-IBS. Fourth, this trial was the first clinical study related to combination treatment with western and traditional herbal medicine in Korea to receive approval from the KMFDS. Through the systematic management of the trial under the auspices of the KMFDS, a high quality trial will be performed and the results of this study will provide information for use in further clinical applications.

Although our trial is designed with a small sample size, it has a creative and systematic research design and the results will be useful basic clinical evidence in the field of integrated herbal and conventional drugs for the management of D-IBS.

For peer review only - http://bmjopen.bmj.com/site/about/guidelines.xhtml

Trial status

The research plan and design began in April 2015.

The study received the IRB's final approval after two deliberations. The first submission date was July 01, 2015; the result was a request for revision and supplementation, which was received on July 13, 2015. We filed an application for re-review on August 06, 2015, supplemented with the relevant information, and received the IRB's final approval on August 10, 2015. We also requested that the KMFDS review the trial plan on September 14, 2015 and received approval for the clinical research on December 21, 2015.

The first participant was randomized on March 29, 2016. Recruitment for the study is ongoing. The primary completion date for this trial is anticipated to be December 31, 2017, and the study completion date is expected to be February 28, 2018.

Ethics

Research ethics approval

This trial was approved by the IRB and ethics committee of Catholic Kwandong University International St. Mary's Hospital (approval number IS15MISV0033) and the KMFDS (approval number 30769).

Protocol amendments

An investigator who wishes to change the protocol of the clinical trial plan should first discuss it with the trial director. Thereafter, the examiner should obtain prior approval from the IRB for changes to the protocol (except to prevent immediate injury to the patient, in which case it will be reported to the IRB later). However, when a dangerous situation occurs and immediate treatment is needed, the investigator may report the protocol change to the IRB at a later time.

For peer review only - http://bmjopen.bmj.com/site/about/guidelines.xhtml

Consent

Patients recruited through the announcement will receive a full explanation of the details of the trial in an easy-to-understand manner. The patients and investigators will have a mutual question and answer time before the patient is asked to sign the clinical trial consent. Through these processes, if the patient agrees to participate in the trial, a signed consent form will be obtained.

Confidentiality

All patient information will be anonymized with initials or symbols and all related investigators will keep the trial results confidential. The trial director will keep the signed consent forms and prepare a list that will be used to confirm the patient identities.

Post-trial care

If the patients experience unexpected accidents or injuries, appropriate compensation will be made by the Federal Insurance Company Korea (12th Floor Ferrum Tower 66 Suha-Dong Jung-gu Seoul, 100210, South Korea), according to the patient compensation rules of the trial. Additionally, in the case of an emergency, the patients will receive appropriate medical care at the Catholic Kwandong University International, St. Mary's Hospital.

Dissemination

The results of the trial will be disseminated through a peer-reviewed journal and/or conference presentations.

Contributors

For peer review only - http://bmjopen.bmj.com/site/about/guidelines.xhtml

Joong Il Kim, Pumsoo Kim, Jin-Hyun Lee, and Jung-Han Lee contributed to the protocol design and writing of the manuscript. Hyekyung Ha is responsible for monitoring the process of the trial. Na-rae Yang, Yoo-Jin Kim, Myong Ki Baeg, Ja Sung Choi, Hye-Jung Kim, Ja-Young Kim and Yun-Young Sunwoo provide advice on the process of the trial. Tae-Yong Park is responsible for writing the manuscript and also managing and supervising the clinical research. All authors approved the final manuscript.

Funding

This work was supported by a "Construction of Scientific Evidences for Herbal Medicine Formulas (K15250)" grant from the Korea Institute of Oriental Medicine (KIOM).

(Telephone: +82-42-868-9513)

Competing interests

The authors have no competing interests to declare.

Ethics approval

This trial has been approved by the IRB and ethics committee of Catholic Kwandong University International St. Mary's Hospital (approval number IS15MISV0033).

Provenance and peer review

Not commissioned; externally peer reviewed.

Open Access

This is an Open Access article distributed in accordance with the Creative Commons Attribution Non-Commercial (CC BY-NC 4.0) license, which permits others to distribute,

For peer review only

For peer review only - http://bmjopen.bmj.com/site/about/guidelines.xhtml

remix, adapt, build upon this work non-commercially, and license their derivative works on different terms, provided the original work is properly cited and the use is non-commercial. See: http://creativecommons.org/licenses/by-nc/4.0/

© Article author(s) (or their employer(s) unless otherwise stated in the text of the article) 2017. All rights reserved. No commercial use is permitted unless otherwise expressly granted.

REFERENCES

1. Drossman DA. Introduction. The Rome Foundation and Rome III. *Neurogastroenterol Motil* 2007;19(10):783-6. doi: 10.1111/j.1365-2982.2007.01001.x

2. Grundmann O, Yoon SL. Irritable bowel syndrome: epidemiology, diagnosis and treatment: an update for health-care practitioners. *J Gastroenterol Hepatol* 2010;25(4):691-9. doi: 10.1111/j.1440-1746.2009.06120.x

3. Maxion-Bergemann S, Thielecke F, Abel F, et al. Costs of irritable bowel syndrome in the UK and US. *Pharmacoeconomics* 2006;24(1):21-37.

4. Soares RL. Irritable bowel syndrome: a clinical review. *World J Gastroenterol* 2014;20(34):12144-60. doi: 10.3748/wjg.v20.i34.12144

5. Saha L. Irritable bowel syndrome: pathogenesis, diagnosis, treatment, and evidence-based medicine. *World J Gastroenterol* 2014;20(22):6759-73. doi: 10.3748/wjg.v20.i22.6759

6. Chey WD, Kurlander J, Eswaran S. Irritable bowel syndrome: a clinical review. *JAMA* 2015;313(9):949-58. doi: 10.1001/jama.2015.0954

7. Boeckxstaens G, Corazziari ES, Mearin F, et al. IBS and the role of otilonium bromide. *Int J Colorectal Dis* 2013;28(3):295-304. doi: 10.1007/s00384-012-1598-0

8. Boeckxstaens G, Clave P, Corazziari ES, et al. Irritable bowel syndrome: focus on

For peer review only - http://bmjopen.bmj.com/site/about/guidelines.xhtml

otilonium bromide. *Expert Rev Gastroenterol Hepatol* 2014;8(2):131-7. doi: 10.1586/17474124.2014.869477

9. Longstreth GF, Thompson WG, Chey WD, et al. Functional bowel disorders. *Gastroenterology* 2006;130(5):1480-91. doi: 10.1053/j.gastro.2005.11.061

10. Jailwala J, Imperiale TF, Kroenke K. Pharmacologic treatment of the irritable bowel syndrome: a systematic review of randomized, controlled trials. *Ann Intern Med* 2000;133(2):136-47.

11. Liu JP, Yang M, Liu YX, et al. Herbal medicines for treatment of irritable bowel syndrome. *Cochrane Database Syst Rev* 2006(1):CD004116. doi: 10.1002/14651858.CD004116.pub2

12. Palsson OS, Drossman DA. Psychiatric and psychological dysfunction in irritable bowel syndrome and the role of psychological treatments. *Gastroenterol Clin North Am* 2005;34(2):281-303. doi: 10.1016/j.gtc.2005.02.004

13. Song DS, Ryu BH, Park DW, et al. Experimental studies on the efficacy of Samryungbaekchulsan. *J Korean Med* 1993;14(1):179-95.

14. Park JK, Choi SY, Koh KW, et al. Combined utilization with herbal products and prescribed drugs: A result from health examinee-based national survey. *Korean J Health Policy Adm* 2006;16(1):1-16.

15. Duan W. Observation of sixty-five cases on Irritable Bowel Syndrome treated by Trimebutine and Shenlingbaizhu-san granule [Chinese]. *Journal of Practical Traditional Chinese Medicine* 2010;26(5):320.

16. Chen Y. Clinical observation of irritable bowel syndrome treated by shenlingbaizhu-san granule and trimebutine [Chinese]. *Journal of Practical Traditional Chinese Medicine* 2011;27(5):318-9.

17. Li Z, Jiang J. Clinical observation of 80 cases on diarrhea - predominant irritable bowel

For peer review only - http://bmjopen.bmj.com/site/about/guidelines.xhtml

syndrome treated by Shenlingbaizhu-san granule [Chinese]. *Guiding Journal of Traditional Chinese Medicine and Pharmacy* 2013;20(5):41-2.

18. Shin B, Lee B. Frequency Analysis of Clinical Prescriptions in the Korean Medicine Hospital, Pusan National University based on Herb Weight Ratio(2) - Focusing on Prescriptions of Herbal Formula Study Textbook[Korean]. *Herbal Formula Science* 2015;23(1):77-89. doi: dx.doi.org/10.14374/HFS.2015.23.1.077

19. Martinez-Cutillas M, Gil V, Gallego D, et al. Mechanisms of action of otilonium bromide (OB) in human cultured smooth muscle cells and rat colonic strips. *Neurogastroenterol Motil* 2013;25(12):e803-12. doi: 10.1111/nmo.12206

20. Traini C, Cipriani G, Evangelista S, et al. Chronic treatment with otilonium bromide induces changes in L-type Ca(2)(+) channel, tachykinins, and nitric oxide synthase expression in rat colon muscle coat. *Neurogastroenterol Motil* 2013;25(11):e728-39. doi: 10.1111/nmo.12197

21. Traini C, Evangelista S, Girod V, et al. Repeated otilonium bromide administration prevents neurotransmitter changes in colon of rats underwent to wrap restraint stress. *J Cell Mol Med* 2017;21(4):735-45. doi: 10.1111/jcmm.13016

22. Chmielewska-Wilkon D, Reggiardo G, Egan CG. Otilonium bromide in irritable bowel syndrome: a dose-ranging randomized double-blind placebo-controlled trial. *World J Gastroenterol* 2014;20(34):12283-91. doi: 10.3748/wjg.v20.i34.12283

23. Clave P, Acalovschi M, Triantafillidis JK, et al. Randomised clinical trial: otilonium bromide improves frequency of abdominal pain, severity of distention and time to relapse in patients with irritable bowel syndrome. *Aliment Pharmacol Ther* 2011;34(4):432-42. doi: 10.1111/j.1365-2036.2011.04730.x

24. Kwon JG, Park KS, Park JH, et al. [Guidelines for the treatment of irritable bowel syndrome]. *Korean J Gastroenterol* 2011;57(2):82-99.

For peer review only - http://bmjopen.bmj.com/site/about/guidelines.xhtml

25. Li C, Zuo M, Wang L, et al. Clinical observation of diarrhea - predominant irritable bowel syndrome treated by Shenlingbaizhu-san and Paroxetine [Chinese]. *Chinese Journal of Traditional Medical Science and Technology* 2010;17(2):154-5.

26. Leung WK, Wu JC, Liang SM, et al. Treatment of diarrhea-predominant irritable bowel syndrome with traditional Chinese herbal medicine: a randomized placebo-controlled trial. *Am J Gastroenterol* 2006;101(7):1574-80. doi: 10.1111/j.1572-0241.2006.00576.x

27. Ko SJ, Han G, Kim SK, et al. Effect of korean herbal medicine combined with a probiotic mixture on diarrhea-dominant irritable bowel syndrome: a double-blind, randomized, placebo-controlled trial. *Evid Based Complement Alternat Med* 2013;2013:824605. doi: 10.1155/2013/824605

28. Whitehead AL, Julious SA, Cooper CL, et al. Estimating the sample size for a pilot randomised trial to minimise the overall trial sample size for the external pilot and main trial for a continuous outcome variable. *Stat Methods Med Res* 2016;25(3):1057-73. doi: 10.1177/0962280215588241

29. Cha BK, Jung MS, Choi CH, et al. The effect of a multispecies probiotic mixture on the symptoms and fecal microbiota in diarrhea-dominant irritable bowel syndrome: a randomized, double-blind, placebo-controlled trial. *J Clin Gastroenterol* 2012;46(3):220-7. doi: 10.1097/MCG.0b013e31823712b1

30. Kim HJ, Vazquez Roque MI, Camilleri M, et al. A randomized controlled trial of a probiotic combination VSL# 3 and placebo in irritable bowel syndrome with bloating. *Neurogastroenterol Motil* 2005;17(5):687-96. doi: 10.1111/j.1365-2982.2005.00695.x

31. Drossman DA, Patrick DL, Whitehead WE, et al. Further validation of the IBS-QOL: a disease-specific quality-of-life questionnaire. *Am J Gastroenterol* 2000;95(4):999-1007. doi: 10.1111/j.1572-0241.2000.01941.x

For peer review only - http://bmjopen.bmj.com/site/about/guidelines.xhtml

32. Lewis SJ, Heaton KW. Stool form scale as a useful guide to intestinal transit time. *Scand J Gastroenterol* 1997;32(9):920-4. doi: 10.3109/00365529709011203

33. Sugaya N, Izawa S, Saito K, et al. Effect of prolonged stress on the adrenal hormones of individuals with irritable bowel syndrome. *Biopsychosoc Med* 2015;9(1):4. doi: 10.1186/s13030-015-0031-7

34. Fadgyas-Stanculete M, Buga AM, Popa-Wagner A, et al. The relationship between irritable bowel syndrome and psychiatric disorders: from molecular changes to clinical manifestations. *J Mol Psychiatry* 2014;2(1):4. doi: 10.1186/2049-9256-2-4

35. Shi J, Tong Y, Shen JG, et al. Effectiveness and safety of herbal medicines in the treatment of irritable bowel syndrome: a systematic review. *World J Gastroenterol* 2008;14(3):454-62.

36. Zhong Y, Wu Y. Forty cases of diarrhea - predominant irritable bowel syndrome treated by Shenlingbaizhu-san granule and Montmorillonite powder [Chinese]. *Jiangxi Journal of Traditional Chinese Medicine* 2013;44(7):45-6.

37. Xiong B. Thirty cases of irritable bowel syndrome treated by Shenlingbaizhu-san granule and Probiotics [Chinese]. *Jiangxi Journal of Traditional Chinese Medicine* 2011;42(5):22-3.

38. Battaglia G, Morselli-Labate AM, Camarri E, et al. Otilonium bromide in irritable bowel syndrome: a double-blind, placebo-controlled, 15-week study. *Aliment Pharmacol Ther* 1998;12(10):1003-10.

39. Evangelista S. Otilonium bromide: a selective spasmolytic for the gastrointestinal tract. *J Int Med Res* 1999;27(5):207-22. doi: 10.1177/030006059902700501

40. Giachetti A. Pharmacological studies on otilonium bromide. *Ital J Gastroenterol* 1991;23(8 Suppl 1):56-9.

For peer review only

For peer review only - http://bmjopen.bmj.com/site/about/guidelines.xhtml

Figure legend

Figure 1. Flow chart of Trial. GIIM: Gastrointestinal internal medicine, FM: Family medicine, D-IBS: diarrhea-irritable bowel syndrome, P: placebo, OB: otilonium bromide, SRS: Samryungbaekchulsan

For peer review only

For peer review only - http://bmjopen.bmj.com/site/about/guidelines.xhtml

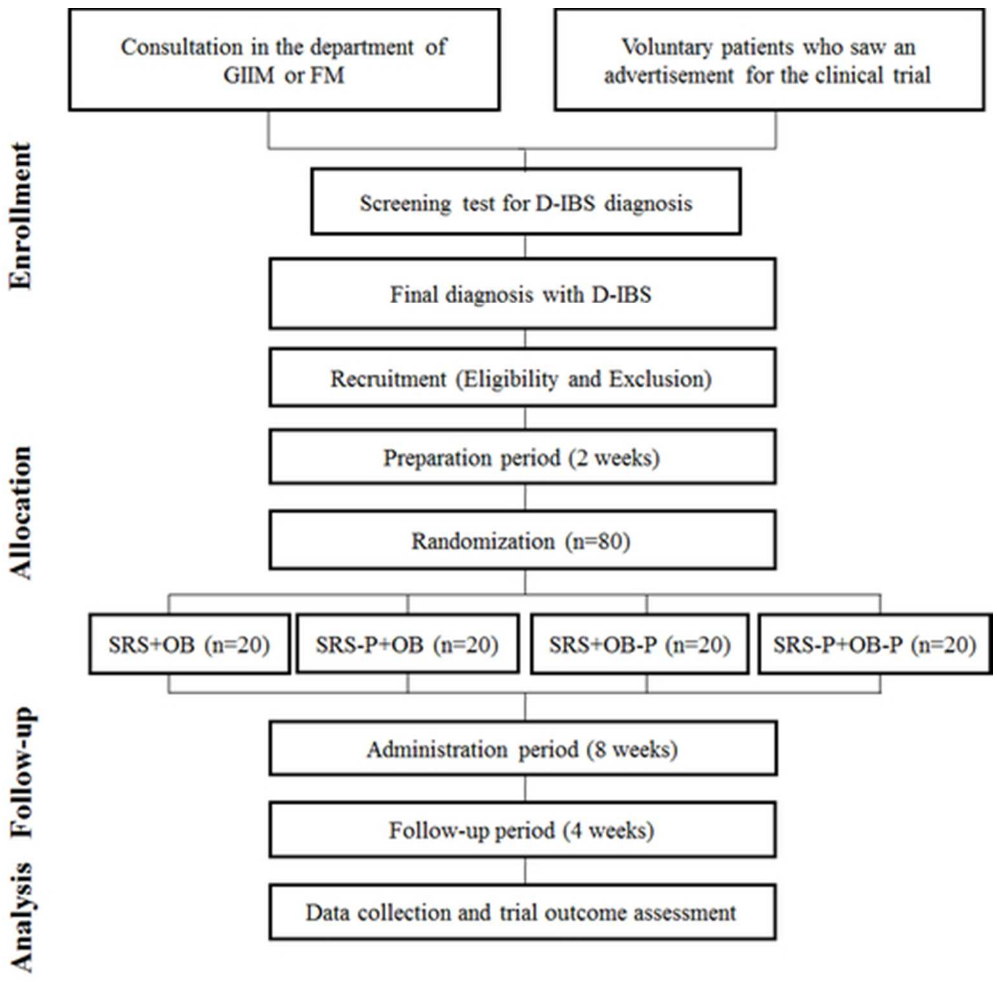

Figure 1. Flow chart of Trial. GIIM: Gastrointestinal internal medicine, FM: Family medicine, D-IBS: diarrhea-irritable bowel syndrome, P: placebo, OB: otilonium bromide, SRS: Samryungbaekchulsan

44x43mm (300 x 300 DPI)

STANDARD PROTOCOL ITEMS: RECOMMENDATIONS FOR INTERVENTIONAL TRIALS

SPIRIT 2013 Checklist: Recommended items to address in a clinical trial protocol and related documents*

Section/item	Item No	Description	Addressed on page number
**Administrative information**			
Title	1	Descriptive title identifying the study design, population, interventions, and, if applicable, trial acronym	P1
Trial registration	2a	Trial identifier and registry name. If not yet registered, name of intended registry	P4
	2b	All items from the World Health Organization Trial Registration Data Set	Attachment
Protocol version	3	Date and version identifier	P4
Funding	4	Sources and types of financial, material, and other support	P1, P24
Roles and responsibilities	5a	Names, affiliations, and roles of protocol contributors	P1, P23
	5b	Name and contact information for the trial sponsor	P1, P24
	5c	Role of study sponsor and funders, if any, in study design; collection, management, analysis, and interpretation of data; writing of the report; and the decision to submit the report for publication, including whether they will have ultimate authority over any of these activities	N/A
	5d	Composition, roles, and responsibilities of the coordinating centre, steering committee, endpoint adjudication committee, data management team, and other individuals or groups overseeing the trial, if applicable (see Item 21a for data monitoring committee)	P8-9, P11-13, P17-19

For peer review only

**Introduction**			
Background and rationale	6a	Description of research question and justification for undertaking the trial, including summary of relevant studies (published and unpublished) examining benefits and harms for each intervention	P5-6
	6b	Explanation for choice of comparators	P6
Objectives	7	Specific objectives or hypotheses	P7-8
Trial design	8	Description of trial design including type of trial (eg, parallel group, crossover, factorial, single group), allocation ratio, and framework (eg, superiority, equivalence, noninferiority, exploratory)	P8-9
**Methods: Participants, interventions, and outcomes**			
Study setting	9	Description of study settings (eg, community clinic, academic hospital) and list of countries where data will be collected. Reference to where list of study sites can be obtained	P8
Eligibility criteria	10	Inclusion and exclusion criteria for participants. If applicable, eligibility criteria for study centres and individuals who will perform the interventions (eg, surgeons, psychotherapists)	P9-12
Interventions	11a	Interventions for each group with sufficient detail to allow replication, including how and when they will be administered	P12-14
	11b	Criteria for discontinuing or modifying allocated interventions for a given trial participant (eg, drug dose change in response to harms, participant request, or improving/worsening disease)	P14-17
	11c	Strategies to improve adherence to intervention protocols, and any procedures for monitoring adherence (eg, drug tablet return, laboratory tests)	P14-17,19
	11d	Relevant concomitant care and interventions that are permitted or prohibited during the trial	P14
Outcomes	12	Primary, secondary, and other outcomes, including the specific measurement variable (eg, systolic blood pressure), analysis metric (eg, change from baseline, final value, time to event), method of aggregation (eg, median, proportion), and time point for each outcome. Explanation of the clinical relevance of chosen efficacy and harm outcomes is strongly recommended	P14-16
Participant timeline	13	Time schedule of enrolment, interventions (including any run-ins and washouts), assessments, and visits for participants. A schematic diagram is highly recommended (see Figure)	P8-9, Figure1

For peer review only - http://bmjopen.bmj.com/site/about/guidelines.xhtml

Sample size	14	Estimated number of participants needed to achieve study objectives and how it was determined, including clinical and statistical assumptions supporting any sample size calculations	__P9__________
Recruitment	15	Strategies for achieving adequate participant enrolment to reach target sample size	__P11-12______

Methods: Assignment of interventions (for controlled trials)

Allocation:

Sequence generation	16a	Method of generating the allocation sequence (eg, computer-generated random numbers), and list of any factors for stratification. To reduce predictability of a random sequence, details of any planned restriction (eg, blocking) should be provided in a separate document that is unavailable to those who enrol participants or assign interventions	__P11-12______
Allocation concealment mechanism	16b	Mechanism of implementing the allocation sequence (eg, central telephone; sequentially numbered, opaque, sealed envelopes), describing any steps to conceal the sequence until interventions are assigned	__P12_________
Implementation	16c	Who will generate the allocation sequence, who will enrol participants, and who will assign participants to interventions	__P11-12______
Blinding (masking)	17a	Who will be blinded after assignment to interventions (eg, trial participants, care providers, outcome assessors, data analysts), and how	__P11-12______
	17b	If blinded, circumstances under which unblinding is permissible, and procedure for revealing a participant's allocated intervention during the trial	__P11-12______

Methods: Data collection, management, and analysis

Data collection methods	18a	Plans for assessment and collection of outcome, baseline, and other trial data, including any related processes to promote data quality (eg, duplicate measurements, training of assessors) and a description of study instruments (eg, questionnaires, laboratory tests) along with their reliability and validity, if known. Reference to where data collection forms can be found, if not in the protocol	__P17-19______
	18b	Plans to promote participant retention and complete follow-up, including list of any outcome data to be collected for participants who discontinue or deviate from intervention protocols	__P8-9________

For peer review only - http://bmjopen.bmj.com/site/about/guidelines.xhtml

Data management	19	Plans for data entry, coding, security, and storage, including any related processes to promote data quality (eg, double data entry; range checks for data values). Reference to where details of data management procedures can be found, if not in the protocol	P17,19
Statistical methods	20a	Statistical methods for analysing primary and secondary outcomes. Reference to where other details of the statistical analysis plan can be found, if not in the protocol	P17-19
	20b	Methods for any additional analyses (eg, subgroup and adjusted analyses)	P17-19
	20c	Definition of analysis population relating to protocol non-adherence (eg, as randomised analysis), and any statistical methods to handle missing data (eg, multiple imputation)	P17-19
**Methods: Monitoring**			
Data monitoring	21a	Composition of data monitoring committee (DMC); summary of its role and reporting structure; statement of whether it is independent from the sponsor and competing interests; and reference to where further details about its charter can be found, if not in the protocol. Alternatively, an explanation of why a DMC is not needed	P19
	21b	Description of any interim analyses and stopping guidelines, including who will have access to these interim results and make the final decision to terminate the trial	P17,19
Harms	22	Plans for collecting, assessing, reporting, and managing solicited and spontaneously reported adverse events and other unintended effects of trial interventions or trial conduct	P15-17
Auditing	23	Frequency and procedures for auditing trial conduct, if any, and whether the process will be independent from investigators and the sponsor	P19
**Ethics and dissemination**			
Research ethics approval	24	Plans for seeking research ethics committee/institutional review board (REC/IRB) approval	P3, P22
Protocol amendments	25	Plans for communicating important protocol modifications (eg, changes to eligibility criteria, outcomes, analyses) to relevant parties (eg, investigators, REC/IRBs, trial participants, trial registries, journals, regulators)	P22

For peer review only - http://bmjopen.bmj.com/site/about/guidelines.xhtml

Consent or assent	26a	Who will obtain informed consent or assent from potential trial participants or authorised surrogates, and how (see Item 32)	P22-23
	26b	Additional consent provisions for collection and use of participant data and biological specimens in ancillary studies, if applicable	N/A
Confidentiality	27	How personal information about potential and enrolled participants will be collected, shared, and maintained in order to protect confidentiality before, during, and after the trial	P23
Declaration of interests	28	Financial and other competing interests for principal investigators for the overall trial and each study site	P24
Access to data	29	Statement of who will have access to the final trial dataset, and disclosure of contractual agreements that limit such access for investigators	P17
Ancillary and post-trial care	30	Provisions, if any, for ancillary and post-trial care, and for compensation to those who suffer harm from trial participation	P23
Dissemination policy	31a	Plans for investigators and sponsor to communicate trial results to participants, healthcare professionals, the public, and other relevant groups (eg, via publication, reporting in results databases, or other data sharing arrangements), including any publication restrictions	P3, 23
	31b	Authorship eligibility guidelines and any intended use of professional writers	N/A
	31c	Plans, if any, for granting public access to the full protocol, participant-level dataset, and statistical code	N/A
**Appendices**			
Informed consent materials	32	Model consent form and other related documentation given to participants and authorised surrogates	Attachment
Biological specimens	33	Plans for collection, laboratory evaluation, and storage of biological specimens for genetic or molecular analysis in the current trial and for future use in ancillary studies, if applicable	P15-16

*It is strongly recommended that this checklist be read in conjunction with the SPIRIT 2013 Explanation & Elaboration for important clarification on the items. Amendments to the protocol should be tracked and dated. The SPIRIT checklist is copyrighted by the SPIRIT Group under the Creative Commons "Attribution-NonCommercial-NoDerivs 3.0 Unported" license.

For peer review only - http://bmjopen.bmj.com/site/about/guidelines.xhtml
